# Evaluation of the Synthetic Scope and the Reaction Pathways of Proton‐Coupled Electron Transfer with Redox‐Active Guanidines in C−H Activation Processes

**DOI:** 10.1002/chem.202003424

**Published:** 2020-11-03

**Authors:** Ute Wild, Petra Walter, Olaf Hübner, Elisabeth Kaifer, Hans‐Jörg Himmel

**Affiliations:** ^1^ Institut für Anorganische Chemie Ruprecht-Karls-Universität Heidelberg Im Neuenheimer Feld 270 69120 Heidelberg Germany

**Keywords:** C−H activation, electron transfer, oxidation, oxidative coupling, redox chemistry

## Abstract

Proton‐coupled electron transfer (PCET) is currently intensively studied because of its importance in synthetic chemistry and biology. In recent years it was shown that redox‐active guanidines are capable PCET reagents for the selective oxidation of organic molecules. In this work, the scope of their PCET reactivity regarding reactions that involve C−H activation is explored and kinetic studies carried out to disclose the reaction mechanisms. Organic molecules with potential up to 1.2 V vs. ferrocenium/ferrocene are efficiently oxidized. Reactions are initiated by electron transfer, followed by slow proton transfer from an electron‐transfer equilibrium.

## Graphical abstract

**There's a good PECT**! Redox‐active guanidines are used in proton‐coupled electron transfer (PCET) reactions that involve C−H bond cleavage. The study reveals the scope and the mechanism of PCET reactions with and without addition of a strong acid. Substrates with potentials of up to 1.2 V versus ferrocenium/ferrocene could be oxidized in stepwise PCET processes initiated by electron transfer. Experiments with guanidine derivatives show the possibility of tuning the PCET reactivity.

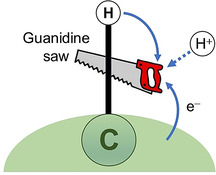

## Introduction

Proton‐coupled electron transfer is of key importance for the progress in modern synthetic chemistry[^1^, ^2^] because it allows, for example, selective and green oxidation of organic substrates, including C−H activation processes, carbon dioxide conversion and water splitting. In the last decades, important advancements were made concerning theory, concepts and synthetic applications of PCET, helping to decode biological processes and paving the way for new applications in synthetic chemistry.[^1^, ^2^, ^3^, ^4^, ^5^, ^6^, ^7^, ^8^] Systematic explorations have been undertaken on how the levelling effect, diminishing the effect of derivatisations (by which the redox potential or the p*K*_a_ value could be tuned) on the PCET reactivity, could be circumvented by the design of bidirectional PCET reactions, in which the electron and proton are transferred to different molecules or different sites of the same molecule.[^9^, ^10^] Applications of bidirectional PCET in synthesis were comprehensively reviewed by Knowles.[^11^, ^12^, ^13^] Detailed studies showed how environmental effects (e.g., hydrogen‐bonding, the solvent polarity or the presence of acids) could be used to enable or speed up PCET reactions. The accumulation of charges in photoredox catalytic systems could be avoided by PCET, allowing the accumulation of oxidative and reductive equivalents instead of charges.[^14^, ^15^] Conceptual work demonstrated how electron transfer could initiate proton movement over large distances. Hence, first proton shuttles were designed in which the transfer of several protons within hydrogen‐bonds is triggered by electron transfer.[^16^, ^17^] Hydrogen‐bonding triggered by redox processes could be employed in sensor devices, and in hydrogen‐bonded molecular shuttles that might be adopted as a basis for artificial molecular machines.

Hence, in theory, an extensive, rational and in some areas biomimetic use of organic PCET reagents in synthetic chemistry is now possible. However, progress does not only depend on a detailed knowledge of the mechanisms and the development of advanced concepts. Due to the various areas of use of PCET reactions in modern synthetic chemistry, the availability of a larger number of PCET compound classes is required. Many applications still suffer from the liability of the known organic PCET reagents to side reactions, prohibiting the formation or diminishing the yield of the desired product. In stoichiometric reactions, an excess of the PCET reagent is often required to obtain good yields. High catalyst loadings are necessary in a number of catalytic reactions, making them unattractive for large‐scale processes. Some reactions (e.g., Scholl‐type aryl–aryl coupling reactions) need to be carried out in highly acidic media, which could initiate degradation of the PCET reagent or the organic substrate. Photochemical applications are still limited because of the relatively small number of available photoactive PCET reagents. Finally, frequently employed quinones such as 2,3‐dichloro‐5,6‐dicyano‐1,4‐benzoquinone (DDQ) and tetrachloro‐1,4‐benzoquinone (chloranil, CA), are highly toxic. Therefore, it is essential to develop new classes of PCET reagents to overcome limitations concerning stability, reactivity, and toxicity of traditionally applied compounds. Numerous fields in synthetic chemistry could profit immensely from the development of such new PCET reagents.

**Figure 1:**
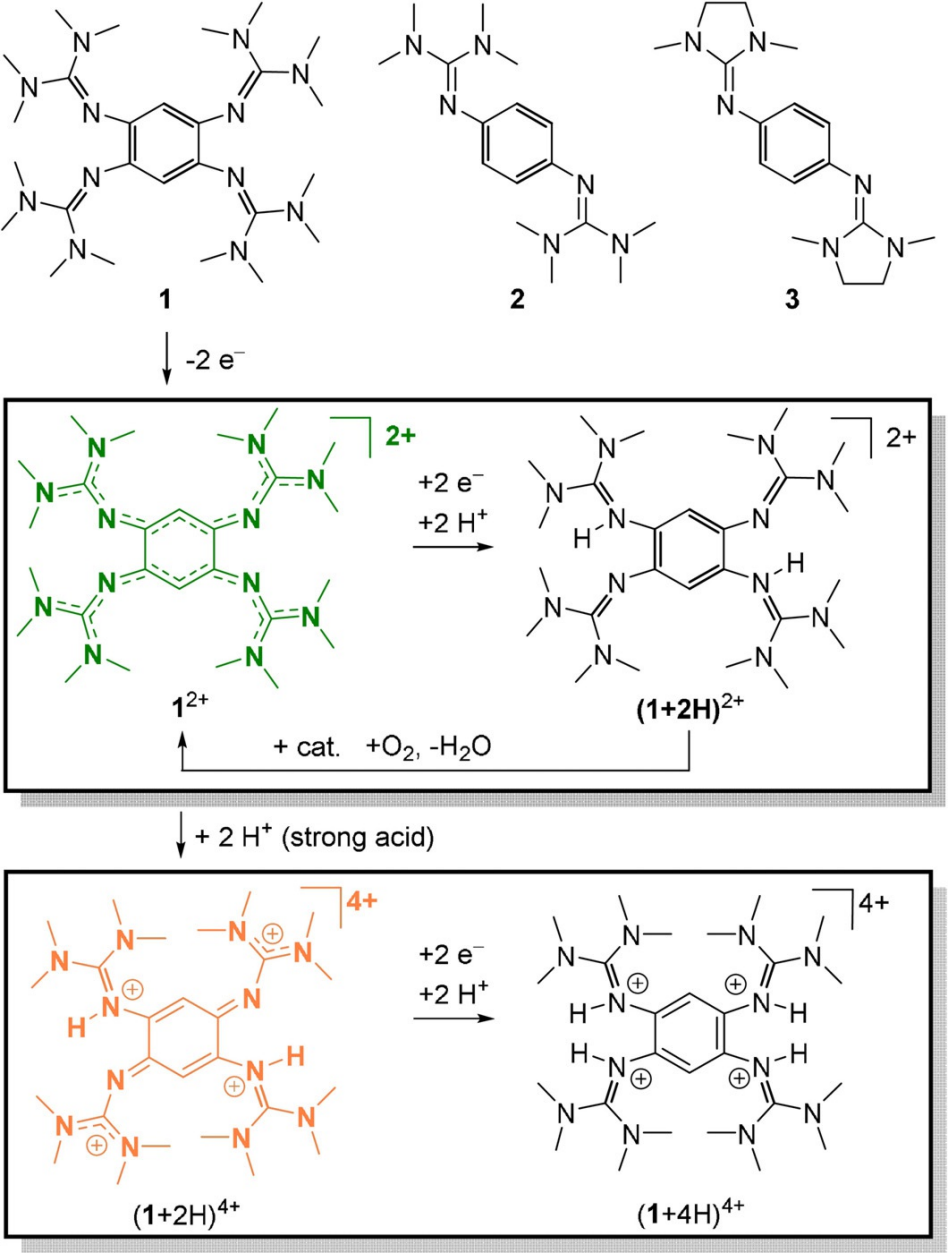
The three redox‐active guanidines studied in this work and equations to illustrate the PCET reactivity of **1** ^2+^.

In the last years, we developed redox‐active guanidines as a new class of capable PCET reagents.^[18]^ We already studied to some extent the PCET chemistry of 1,2,4,5‐tetrakis(tetramethylguanidino)‐benzene (**1**). It is readily accessible from commercially available 1,2,4,5‐tetrakisamino‐benzene‐tetrahydrochloride,^[19]^ and a relatively strong electron donor with a redox potential (*E*_1/2_ value) of −0.73 V vs. ferrocenium/ferrocene for the redox couple **1** ^2+^/**1** in CH_3_CN solution.^[20]^ The compound looses two electrons at the same potential. The free radical monocation **1** ^⋅+^ is unstable towards disproportionation into **1** and **1** ^2+^. Hence, it is not formed in mixtures of **1** and **1** ^2+^. On the other hand, the radical monocationic form is stable as a bridging ligand in several dinuclear late‐transition‐metal complexes.[^21^, ^22^] A number of salts of **1** ^2+^ were fully characterized, including **1**(PF_6_)_2_ ^[23]^ and **1**(BF_4_)_2_ ^[24]^ used in this work, and **1**(I_3_)_2_.^[19]^ Also, the nitrogen‐rich **1**[N(CN)_2_]_2_ was prepared,^[25]^ that melts above 200 °C and decomposes smoothly at 220 °C, demonstrating its excellent thermal stability. In the dication **1** ^2+^, the bond length between the carbons in 1‐ and 2‐positions and that between the carbons in 4‐ and 5‐positions (all directly bound to guanidino groups) are considerably elongated, in line with the Lewis structure in Figure 1. The twofold protonated, reduced form (**1**+2H)^2+^ could be re‐oxidized to the dication **1** ^2+^ by dioxygen under mild conditions with a copper or cobalt catalyst,[^26^, ^27^] opening up the possibility to use **1** as redox catalyst for the green aerobic oxidation of a variety of organic molecules.^[27]^ Protonated redox‐active guanidines were also integrated as sacrificial reductant in tin and lead iodate materials to prohibit metal oxidation by dioxygen diffusing into the material.^[28]^ The scope for stoichiometric PCET reactions with **1** ^2+^ could be greatly extended in the presence of strong acids,^[29]^ leading in the first place to di‐protonation of **1** ^2+^ to give the tetracation (**1**+2H)^4+^ (see Lewis structure in Figure 1)^[24]^ with ca. 0.7 V higher oxidation potential.

In this work we elaborate on the PCET chemistry of the three redox‐active guanidines 1,2,4,5‐tetrakis(tetramethylguanidino)‐benzene (**1**), 1,4‐bis(tetramethylguanidino)‐benzene (**2**), and the new compound 1,4‐bis(*N*,*N*′‐dimethylethylene)guanidino‐benzene (**3**) shown in Figure 1, in PCET reactions that involve C−H activation. The conversion was followed by NMR or UV/Vis spectroscopy, and the yields were estimated by NMR signal integration. Please note that the equations shown in the following do not account for protonation equilibria that are observed for the reduced, protonated guanidines arising as products in these reactions (see the Supporting Information for NMR spectra of the protonated compounds). Also, in the presence of strong acids all guanidino groups become protonated.

## Results and Discussion

### Expansion of the scope of PCET chemistry with 1^2+^

**Scheme 1:**
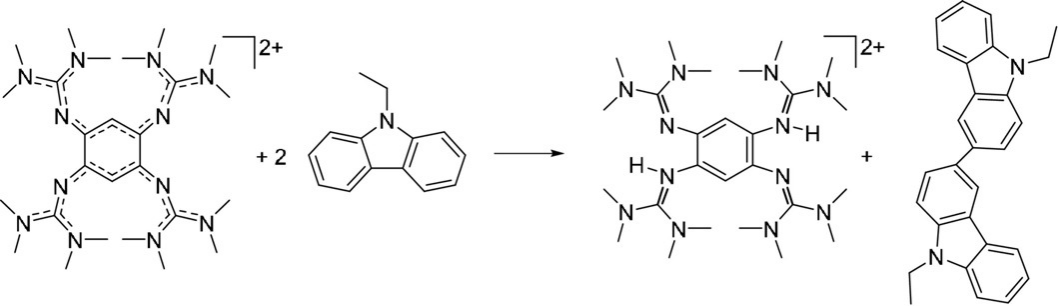
Oxidative intermolecular aryl‐aryl coupling of *N*‐ethylcarbazole to *N*,*N*′‐diethyl‐3,3′‐bicarbazole. Reagents and conditions: acetonitrile, **1**(ClO_4_)_2_ (1 equiv), HBF_4_⋅OEt_2_ (16 equiv), 15 min at 0 °C, 45 min at r.t., 95 % yield.

We decided to test first the oxidative coupling of *N*‐ethylcarbazole to *N*,*N*′‐diethyl‐3,3′‐bicarbazole (Scheme 1). *N*‐Ethylcarbazole exhibits an *E*_ox_ value of 1.12 V vs. SCE,^[30]^ translating into a value of 0.66 V vs. Fc^+^/Fc.^[31]^ Since previous experiments showed that substrates with a redox potential of up to 0.77 V vs. Fc^+^/Fc could be oxidized,^[28]^ it was clear that the potential is not too high for a reaction with initial electron transfer. In our experiments, one equivalent of **1**(BF_4_)_2_ or **1**(ClO_4_)_2_ (see the Supporting Information for synthesis and characterization of this new salt) was reacted with *N*‐ethylcarbazole in the presence of 16 equivalents of HBF_4_⋅OEt_2_. Acetonitrile was chosen as solvent, since the protonated, oxidized guanidine (**1**+2H)^4+^ that forms immediately in the presence of a strong acid, is not soluble in nonpolar solvents such as CH_2_Cl_2_. Indeed, near quantitative conversion (94 % and 95 %, respectively) was obtained within 1 h reaction time. Recently, Venkatakrishnan et al. showed that reaction of DDQ or CA with *N*‐ethylcarbazole gives quantitative yield (>99 %) of the bicarbazole coupling product in very short time when carried out in CH_2_Cl_2_ solution with an excess of methanesulfonic acid.[^32^, ^33^] However, two equivalents of the quinone (fourfold excess) had to be used; the yield decreased to 79 % for CA and 87 % for DDQ when only one equivalent was used (like in our experiments). Moreover, the yield decreased to 83 % when the reaction was carried out in CH_3_CN instead of CH_2_Cl_2_. Hence the results demonstrate that salts of **1** ^2+^ are valuable alternatives to toxic CA or DDQ in aryl‐aryl coupling reactions.

**Scheme 2:**
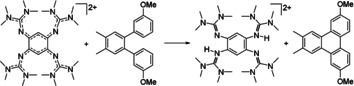
Intramolecular oxidative coupling of 3,3′′‐dimethoxy‐3′,4′‐dimethyl‐*o*‐terphenyl to the corresponding dimethoxy‐triphenylene. Reagents and conditions: acetonitrile, **1**(BF_4_)_2_ (1.5 equiv), HBF_4_⋅OEt_2_ (20 equiv), 10 min at 0 °C, 120 min at r.t., 76 % yield.

We then inspected the oxidative coupling of 3,3′′‐dimethoxy‐3′,4′‐dimethyl‐*o*‐terphenyl to 3,10‐dimethoxy‐6,7‐dimethyltriphenylene with salts of **1** ^2+^, again in the presence of excess (ca. 20 equivalents) of HBF_4_⋅OEt_2_ (Scheme 2). With **1**(BF_4_)_2_, a yield of 76 % was obtained after 130 min reaction time; use of **1**(PF_6_)_2_ resulted in a slightly lower yield of 71 %. This reaction demonstrates that salts of **1** ^2+^ could oxidize compounds with redox potentials of 1.2 V vs. Fc^+^/Fc, thus being at least similar in their oxidizing capability to DDQ or CA. The strong acid leads to double‐protonation of **1** ^2+^ to (**1**+2H)^4+^,^[24]^ which is the oxidant in this reaction. Previous quantum‐chemical calculations indicate that (**1**+2H)^4+^ is an even stronger oxidant in PCET reactions than DDQ or CA.^[28]^ The reaction requires the use of 1.5 equivalents of **1** ^2+^. This can be explained by the considerably lower redox potential of the triphenylene product (0.72 V vs. Fc^+^/Fc in CH_2_Cl_2_) compared with the reactant (1.22 V vs. Fc^+^/Fc in CH_2_Cl_2_ for 3,3′′‐dimethoxy‐3′,4′‐dimethyl‐*o*‐terphenyl), leading to preferred oxidation of the product by (**1**+2H)^4+^ to the radical monocation. The formation of this radical cation leads to broad signals in the NMR spectra, and therefore the reaction was quenched before analysis. Oxidative coupling of 3,3′′‐dimethoxy‐3′,4′‐dimethyl‐*o*‐terphenyl could also be carried out with 1.5 equivalent of DDQ and an excess of MeSO_3_H in CH_2_Cl_2_, giving the triphenylene coupling product quantitatively in short time.[^34^, ^35^] A detailed analysis showed that this reaction follows a cation radical (electron transfer) mechanism rather than an arenium ion (proton transfer) mechanism.^[35]^ Similarly, electron transfer between (**1**+2H)^4+^ and 3,3′′‐dimethoxy‐3′,4′‐dimethyl‐*o*‐terphenyl is assumed to occur before proton transfer (see discussion in the section “Comparison between the PCET reactivity of **1^2+^** and **2^2+^**”). Notably, the guanidine PCET reagent could be recycled from the reaction mixture for both reactions. For this purpose it was first separated from the aryl‐aryl coupling product by addition of ether (in which only the coupling product is soluble) and filtration. The reduced, protonated (**1**+2H)^2+^ was then reoxidized to **1** ^2+^ by catalytic oxidation with dioxygen (see the Supporting Information for details).

### Further C−H bond‐cleavage reactions with salts of 1^2+^

**Scheme 3:**
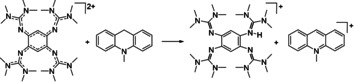
Oxidation of 10‐methyl‐9,10‐dihydroacridane (AcrH_2_). Reagents and conditions: acetonitrile, **1**(PF_6_)_2_ (1 equiv), HBF_4_⋅OEt_2_ (7 equiv), 6.5 h at r.t., 90 % yield.

Having demonstrated the scope of oxidative aryl‐aryl coupling reactions for substrates with potentials of up to 1.2 V vs. Fc^+^/Fc, we next focussed on the oxidation of 1‐benzyl‐1,4‐dihydronicotinamide (BNAH), 10‐methyl‐9,10‐dihydroacridane (AcrH_2_) and 9,10‐dihydroanthracene (AnH_2_) to systematically study PCET reactions without and with addition of a strong acid. 10‐Methyl‐9,10‐dihydroacridane (AcrH_2_) exhibits a bond dissociation energy (BDE) of 308 kJ mol^−1^ in CH_3_CN solution,^[36]^ and an *E*_ox_ value of 0.492 V vs. Fc^+^/Fc in CH_3_CN. With a reduction potential (*E*_red_) of −0.77 V vs. Fc^+^/Fc for **1** ^2+^,^[20]^ the energy gap Δ*G*_el_ for electron transfer between AcrH_2_ and **1** ^2+^ is 1.26 V or 122 kJ mol^−1^, clearly exceeding the assumed limit for conventional electron transfer at standard conditions of ca. 1 V (96.5 kJ mol^−1^).^[37]^ Hence, **1** ^2+^ is too weak to oxidize AcrH_2_. Since electron transfer is supposed to be the first step of the reaction (see discussion in section “Kinetic measurements and mechanistic considerations”), the expectation is that no reaction takes place. Indeed, only traces of *N*‐methylacridinium (AcrH^+^) are observed if the reaction is carried out in the absence of an acid. On the other hand, a high product yield (90 %) in 6.5 h reaction time is obtained upon addition of 7 equivalents of HBF_4_⋅OEt_2_ (Scheme 3). Again, the acid instantly protonates the guanidine to give the strong oxidant (**1**+2H)^4+^ (see Figure 1),^[24]^ decreasing the energy gap between the AcrH_2_ electron donor and the acceptor to ca. 0.5 V, enabling electron‐transfer as initial reaction step. The further excess of acid leads to fast conversion. A small but significant isotope effect was observed when AcrH_2_ was replaced with AcrD_2_ (see the Supporting Information), implying that the rate of proton transfer enters into the overall reaction rate (see discussion below).

**Scheme 4:**
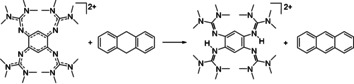
Oxidation of 9,10‐dihydroanthracene. Reagents and conditions: acetonitrile, **1**(PF_6_)_2_ (1 equiv), HBF_4_⋅OEt_2_ (5 equiv), 3 h at r.t., 78 % yield.

The BDE value of 9,10‐dihydroanthracene (ca. 326 kJ mol^−1^) is higher than that of AcrH_2_.^[36]^ Although quantum‐chemical calculations (B3LYP/def2‐TZVP) predict the reaction of **1** ^2+^ with 9,10‐dihydroanthracene (AnH_2_) to give anthracene (An) to be still exothermic (Gibbs free reaction energy of −104.5 kJ mol^−1^ at *ϵ*_r_=38 and −87.7 kJ mol^−1^ at *ϵ*_r_=1), a massive barrier is expected for the unfavoured initial electron transfer step. Therefore, also reaction of **1** ^2+^ with 9,10‐dihydroanthracene requires the presence of an excess of HBF_4_⋅OEt_2_. With 5 equivalents of HBF_4_⋅OEt_2_ and using the salt **1**(PF_6_)_2_, a yield of 78 % anthracene is obtained within 3 h at room temperature (Scheme 4). The guanidine PCET reagent could be recycled (see the Supporting Information). When more HBF_4_⋅OEt_2_ was applied, the rate increased, but the An yield decreased (e.g., 65 % yield after 1.5 h with 9 equivalents of HBF_4_⋅OEt_2_). Interestingly, an induction period with low rate was visible in the conversion versus time plot (see the Supporting Information). A possible explanation is an autocatalytic effect by formation of the anthracene radical. Indeed, NMR spectra recorded after 2 h reaction time displayed broad signals, indicating the presence of radicals in the reaction mixture. By contrast, the signals in the NMR spectra recorded after 3 h were again sharp, arguing for complete conversion of the radical intermediates. For comparison, kinetic measurements for the reaction between anthracene (An) and 9,10‐dihydrophenanthrene (PhenH_2_) to give eventually AnH_2_ and Phen^[38]^ showed that fast reaction between the produced AnH_2_ and the reactant An generates the reactive radical AnH^**⋅**^.

**Scheme 5:**
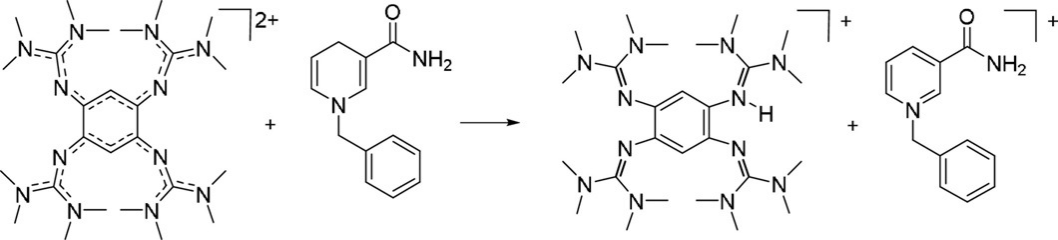
Oxidation of 1‐benzyl‐1,4‐dihydronicotinamide (BNAH). Reagents and conditions: acetonitrile, **1**(PF_6_)_2_ (1 equiv), 24 h at 60 °C, 27 % yield.

Finally, the reaction of **1** ^2+^ with 1‐benzyl‐1,4‐dihydronicotinamide (BNAH) was tested. The addition of a strong acid is not possible in this reaction due to acid‐catalysed hydration of BNAH.^[39]^ The BDE value of BNAH is 284 kJ mol^−1^,^[36]^ and the *E*_ox_ value is 0.259 V vs. Fc^+^/Fc in CH_3_CN. The energy gap Δ*G*_el_ for electron transfer between **1** ^2+^ (*E*_red_=−0.77 V vs. Fc^+^/Fc) and BNAH of 1.0 V is just at the assumed limit value for conventional electron transfer at standard conditions. Hence, reaction between **1** ^2+^ and BNAH might be possible at higher temperatures. Indeed, a slow reaction was observed at a temperature of 60 °C, yielding 27 % of the pyridinium salt after 24 h (Scheme 5). Prolonged reaction times (2 d) lead to degradation of BNAH.

### Comparison between the PCET reactivity of 1^2+^ and 2^2+^

The results assembled in the previous section demonstrate that salts of **1** ^2+^ are capable PCET reagents. However, oxidation of higher potential substrates requires the addition of a strong acid. Since some substrates, for example, BNAH, degrade in the presence of acids, we thought of ways to increase the oxidation power in the absence of acids. We previously showed that the redox potential of 1,4‐bis(tetramethylguanidino)‐benzene (**2**) is significantly higher than that of **1** (*E*_1/2_=−0.21 V vs. Fc^+^/Fc in CH_3_CN for **2** ^[40]^ and −0.73 V for **1** ^[20]^). Although **2** ^2+^ is therefore a stronger oxidant in electron‐transfer reactions, it is not necessarily a stronger oxidant in PCET reactions, since the thermodynamics of PCET reactions depends not only on the potentials, but also on the p*K*_a_ values of the reduced, protonated species. Generally, an increase of the potential is accompanied by a decrease of the p*K*_a_ value of the protonated, reduced form. This “levelling effect” limits the possibility of tuning the PCET reactivity by derivatization. In this section we compare the PCET reactivity of **2** ^2+^ with that of **1** ^2+^ by direct reaction between the oxidized form of one compound with the reduced, doubly protonated form of the other compound.

**Scheme 6:**
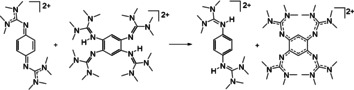
Oxidation of (**1**+2H)^2+^. Reagents and conditions: acetonitrile, **2**(PF_6_)_2_ (1 equiv), 5 min at r.t., >99 % yield.

Our experiments show that **2** ^2+^ reacts fast with (**1**+2H)^2+^ to give (**2**+2H)^2+^ and **1** ^2+^. In an acetonitrile solution with concentrations of 1.25×10^−2^ mol/L for both reactants, quantitative conversion (>99 %) is obtained after 5 min at 25 °C (Scheme 6). On the other hand, no reaction takes place when **1** ^2+^ is mixed with (**2**+2H)^2+^. From these experiments one could deduce that **2** ^2+^ is indeed a stronger PCET reagent than **1** ^2+^, at least in the absence of a strong acid. This result is supported by quantum‐chemical calculations predicting a Gibbs free reaction energy of −84.5 kJ mol^−1^ at *ϵ*_r_=38 and −105.7 kJ mol^−1^ at *ϵ*_r_=1.

**Figure 2:**
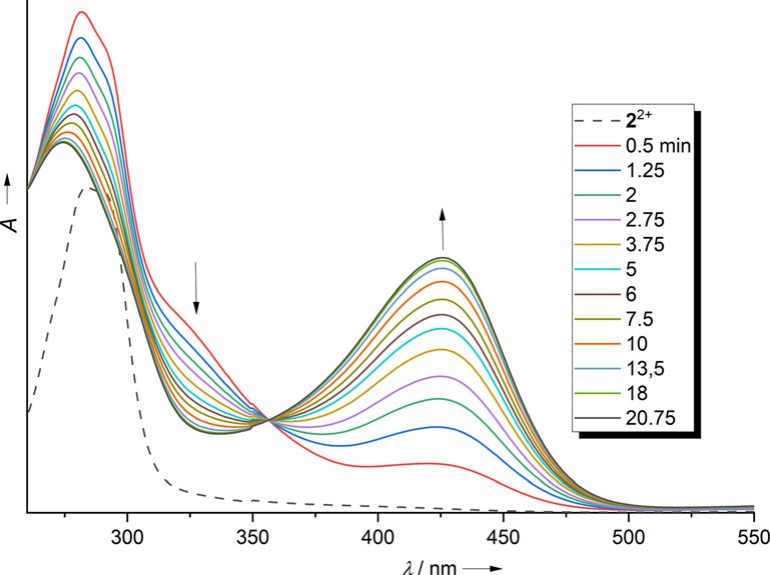
UV/Vis spectra recorded for the 1:1 reaction between **2** ^2+^ and (**1**+2H)^2+^. The strong band at 426 nm signals formation of **1** ^2+^ as one reaction product.

The fast rate of the reaction between **2** ^2+^ and (**1**+2H)^2+^ is evident from the appearance of the green colour characteristic for **1** ^2+^, motivating further analysis by UV/Vis spectroscopy (Figure 2). For equal concentrations (5.6×10^−5^ mol/L) of the two reactants, the reaction is completed after 20 min. The growth of a strong band at 425 nm signals the formation of **1** ^2+^. The presence of an isosbestic point at ca. 357 nm indicates clean conversion, in line with the NMR experiments at higher concentrations. In further experiments, we added 0.25 equivalents of HBF_4_⋅OEt_2_ to establish whether the reaction rate is influenced by the presence of acid (see the Supporting Information). A decrease of the reaction rate upon acid addition was observed. This could be explained by the protonation of the (**1**+2H)^2+^ reactant to give (**1**+4H)^4+^, which is less oxidizable. On the other hand, additional NMR experiments showed that the reaction could not be reversed by addition of larger quantities of HBF_4_⋅OEt_2_. Also, addition of HBF_4_⋅OEt_2_ to a **1** ^2+^/ (**2**+2H)^2+^ mixture only leads to the protonated, oxidized guanidine (**1**+2H)^4+^, but not to PCET.

To analyse the kinetics in more detail, stopped‐flow measurements were conducted in which **2** ^2+^ was applied in excess (concentrations of 3.94×10^−4^ mol/L (ratio **2** ^2+^/(**1**+2H)^2+^=10:1), 7.87 10^−4^ mol/L (20:1), and 1.58 10^−3^ mol/L (40:1)). The results are in line with a pseudo‐first‐order reaction only within the first seconds, hampering the unambiguous identification of the reaction order. On the other hand, the estimated pseudo‐first‐order rate constants increase linearly with the concentration of **2** ^2+^. Hence, the results might be in line with a first‐order reaction in both **2** ^2+^ and (**1**+2H)^2+^ in the initial period (see the Supporting Information for details), and the analysis yielded a second‐order rate constant *k*_H_=257±27 m ^−1^ s^−1^ at room temperature.

The results in this section clearly show that **2** ^2+^ is a stronger PCET oxidant than **1** ^2+^. Additional NMR experiments indicated degradation of **2** ^2+^ to so far unidentified products in the presence of excess HBF_4_⋅OEt_2_ (see the Supporting Information). Therefore most reactions reported in the following were carried out in the absence of acid.

### PCET reactions with 2^2+^

**Scheme 7:**
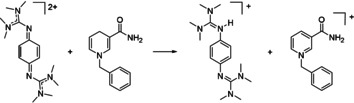
Oxidation of BNAH. Reagents and conditions: acetonitrile, **2**(PF_6_)_2_ (1 equiv), r.t., instantly, >99 % yield.

The reactions of **2** ^2+^ with 1‐benzyl‐1,4‐dihydronicotinamide (BNAH), 10‐methyl‐9,10‐dihydroacridane (AcrH_2_), and 9,10‐dihydroanthracene (AnH_2_) were carried out in the absence of a strong acid. Salts of **2** ^2+^ react very fast with BNAH; quantitative yield (>99 %) was obtained instantly upon mixing BNAH with **2**(PF_6_)_2_ together at room temperature in acetonitrile solution (Scheme 7). With *E*_red_=−0.24 V for **2** ^2+^, the energy for electron transfer, Δ*G*_el_, is 0.50 V (48 kJ mol^−1^) and therefore well below 1 V. Hence the observation of fast reaction is in line with electron transfer in the first step.

**Scheme 8:**
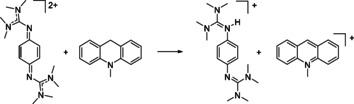
Oxidation of *N*‐methylacridane (AcrH_2_). Reagents and conditions: acetonitrile, **2**(PF_6_)_2_ (2 equiv), 160 min at r.t., 92 % yield.

As expected given its higher potential, reaction of **2** ^2+^ (applied as **2**(PF_6_)_2_ salt) with 10‐methyl‐9,10‐dihydroacridane is slower. NMR experiments showed that a yield of ca. 92 % is obtained after 2.5 h at room temperature using 2 equivalents of **2**(PF_6_)_2_ (Scheme 8). The reaction is further slowed down if only one equivalent of **2**(PF_6_)_2_ is applied, yielding 73 % ArcH^+^ in 3 h at room temperature. Again, this result is in line with electron transfer in the first step, since Δ*G*_el_ is 0.73 V (70 kJ mol^−1^).

**Scheme 9:**
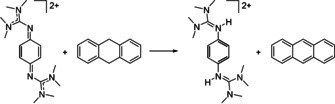
Oxidation of 9,10‐dihydroanthracene. Reagents and conditions: acetonitrile, **2**(PF_6_)_2_ (1 equiv), NH_4_PF_6_ (2 equiv), 50 h at 60 °C, 29 % yield.

The reaction of **2** ^2+^ with 9,10‐dihydroanthracene is again slower. A yield of only 6 % of anthracene is obtained after 50 h at 60 °C. The yield could be increased to 29 % by addition of ca. 2 equivalents of NH_4_PF_6_ (Scheme 9). In contrast to the results obtained for the reaction of **1** ^2+^ with 9,10‐dihydroanthracene in the presence of acid, there was no induction period, and the conversion increased almost linearly with time. Quantum chemical calculations (B3LYP/def2‐TZVP) predict the reaction of **2** ^2+^ with 9,10‐dihydroanthracene to be significantly exothermic (Gibbs free reaction energy of −189.0 kJ mol^−1^ at *ϵ*_r_=38, and −193.4 kJ mol^−1^ at *ϵ*_r_=1). The slow reaction rate could be explained by a substantial barrier for the initial electron‐transfer step. A similar strong correlation between the potential (*E*_ox_ or *E*_1/2_ value) of the PCET reagent and the reaction yield was recently reported for oxidative C−H amination reactions of 9,10‐dihydro‐9‐heteroanthracenes with quinones as PCET reagents.^[41]^

**Scheme 10:**
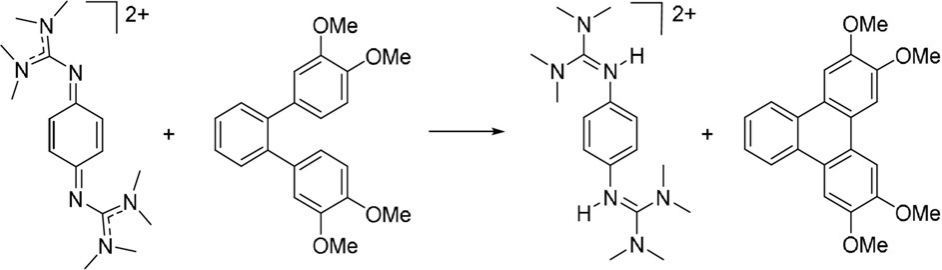
Oxidation of 3,3′′‐4,4′′‐tetramethoxy‐*o*‐terphenyl. Reagents and conditions: acetonitrile, **2**(PF_6_)_2_ (1 equiv), HBF_4_⋅OEt_2_ (12.5 mol %), 10 min at 60 °C, 95 % yield.

Next, we tested the performance of **2** ^2+^ in an aryl‐aryl coupling reaction. Reaction of **2** ^2+^ with 3,3′′‐4,4′′‐tetramethoxy‐*o*‐terphenyl (TMTP) at room temperature did not produce the product in the absence of an acid. The oxidation potential of TMTP is 0.74 V, and hence the energy required for electron transfer, Δ*G*_el_, is 0.98 V, is close to the assumed limit value of 1.0 V. On the other hand, only small amounts of HBF_4_⋅OEt_2_ are required to initiate fast and clean reaction. With 12.5 mol % of HBF_4_⋅OEt_2_, a yield of 95 % is obtained within 10 min reaction time at a temperature of 60 °C (Scheme 10). For comparison, the analogous reaction with **1** ^2+^ in place for **2** ^2+^ required the use of a large excess (21 equiv) of HBF_4_⋅OEt_2_ to reach completion in a relatively short time (45 min at room temperature, 99 % yield).^[28]^ In the case of **2** ^2+^, small amounts of a strong acid could be applied and did not lead to acid‐induced degradation of **2** ^2+^. However, an excess of the acid (e.g., 4 equivalents or more) had to be avoided as it led to quite fast degradation of **2** ^2+^ (see section “Comparison between the PCET reactivity of **1** ^2+^ and **2** ^2+**”**^ and the Supporting Information).

**Scheme 11:**
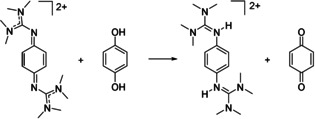
Oxidation of *p*‐dihydrobenzoquinone. Reagents and conditions: acetonitrile, **2**(PF_6_)_2_ (1 equiv), 12 min at 55 °C, >99 % yield.

Finally, we inspected the reaction of **2** ^2+^ with *p*‐dihydro‐ benzoquinone (Scheme 11). NMR experiments (*c*=2.9×10^−2^ mol L^−1^ for both reactants) showed complete conversion (>99 %) within 12 min at 55 °C. At room temperature, 60 min are required (98 % yield). Fits of the conversion versus time curves, obtained for different concentrations of the two reactants, while keeping the 1:1 stoichiometric ratio, and for experiments at different temperatures (see the Supporting Information), gave no satisfactorily results when assuming second‐ or first‐order rate equations. An analysis by UV/Vis spectroscopy is hampered by the close proximity of the absorptions due to reactants and products. Quantum‐chemical calculations (B3LYP/def2‐TZVP) found a Gibbs free reaction energy of −96.1 kJ mol^−1^ at *ϵ*_r_=38 and −107.8 kJ mol^−1^ at *ϵ*_r_=1. For comparison, the analogous reaction with **1** ^2+^ in place for **2** ^2+^ is only slightly exergonic (−11.6 kJ mol^−1^ at *ϵ*_r_=38, calculations with counterions).

### Kinetic measurements and mechanistic considerations

**Figure 3:**
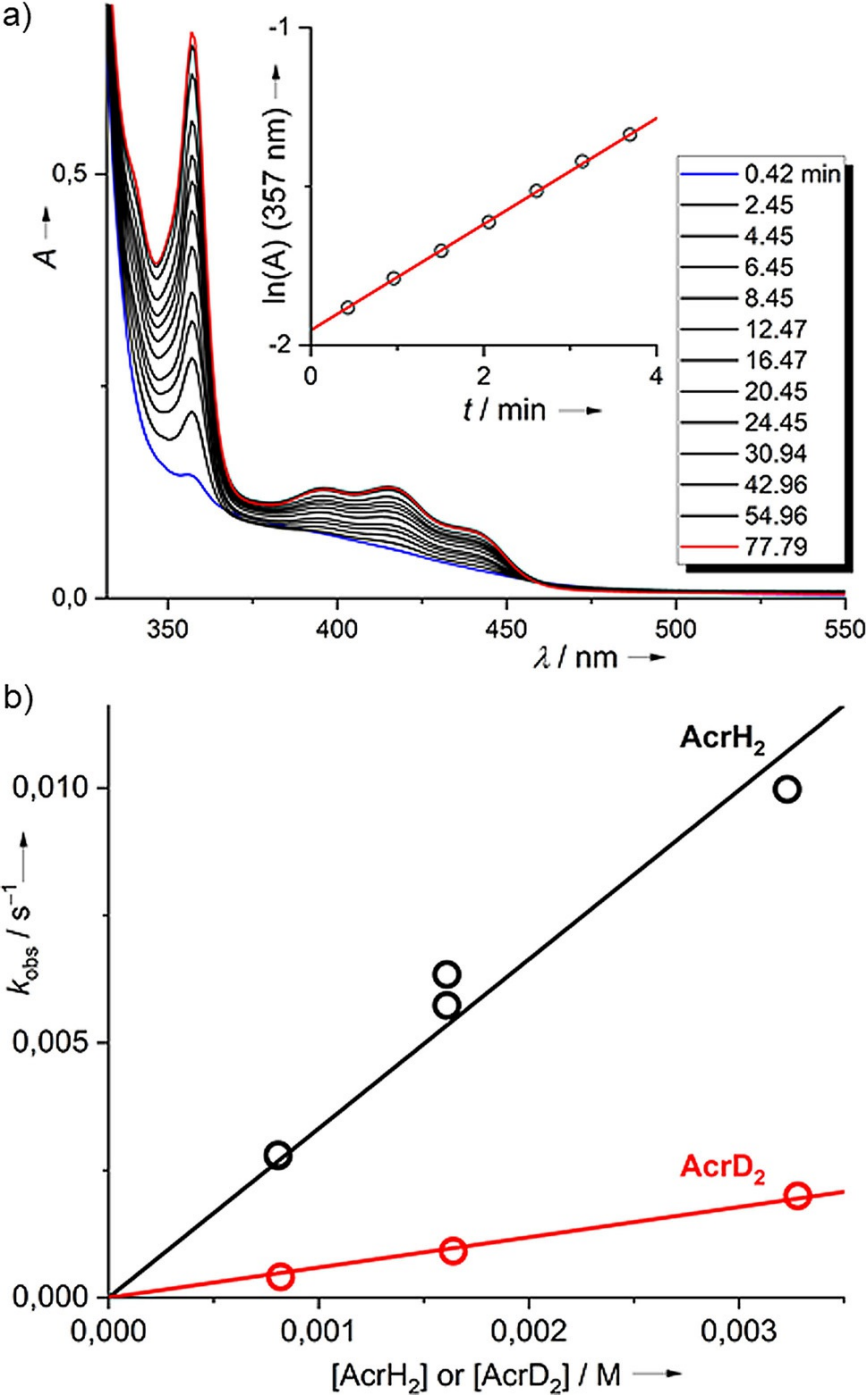
a) Selected UV/Vis spectra for the reaction between **2**(PF_6_)_2_ and AcrH_2_ (10 equiv) in CH_3_CN solution. The inlet shows the ln(*A*) vs. time plot in the first 4 min of the reaction, from which the pseudo‐first‐order rate constant is determined. b) Plot of the pseudo‐first‐order rate constants as a function of the acridane concentration (for an invariant concentration of **2**(PF_6_)_2_ of ca. 8×10^−5^ m). See the Supporting Information for details.

The reaction of **2** ^2+^ with 10‐methyl‐9,10‐dihydroacridane was examined in more detail to derive information about the reaction mechanism. Preliminary NMR experiments point to a slower reaction with ArcD_2_ in place for AcrH_2_ (see the Supporting Information); UV/Vis experiments were conducted for further kinetic analysis. To obtain pseudo‐first‐order conditions, a ten‐, twenty‐ and forty‐fold excess of AcrH_2_ was applied. The formation of *N*‐methylacridinium was clearly visible from the appearance of a typical sharp band at 357 nm and a broader band around 420 nm (with maxima at 395/415/440 nm). The band at 357 nm was chosen for the analysis. Figure 3 a shows the spectra recorded with a tenfold excess of AcrH_2_. In the first 5 min, the reaction nicely follows a pseudo‐first‐order kinetics, as seen from the ln(A) vs. time plot in the inlet. The *k*_obs_ values derived from such fits were plotted as a function of the AcrH_2_ concentration (see Figure 3 b). The slope of this plot gives the second‐order rate constant at room temperature, *k*_H_=3.32±0.16 m ^−1^ s^−1^. For comparison, a ca. 4.5 times larger rate constant (*k*_H_=15 m ^−1^ s^−1^) was previously published for the reaction between chloranil and AcrH_2_ (also in acetonitrile).^[42]^ A similar analysis for AcrD_2_ yielded a second‐order rate constant *k*_D_=0.593±0.021 m ^−1^ s^−1^ (see plot of the *k*_obs_ values as a function of AcrD_2_ concentration in Figure 3 b and the Supporting Information for further details). Thus, a quite large kinetic isotope effect (KIE=*k*_H_/*k*_D_) of 5.6 results, which is nevertheless still smaller than the KIE of 8.8 obtained for the reaction between CA and *N*‐methyl‐acridane (AcrH_2_).^[42]^

**Scheme 12:**
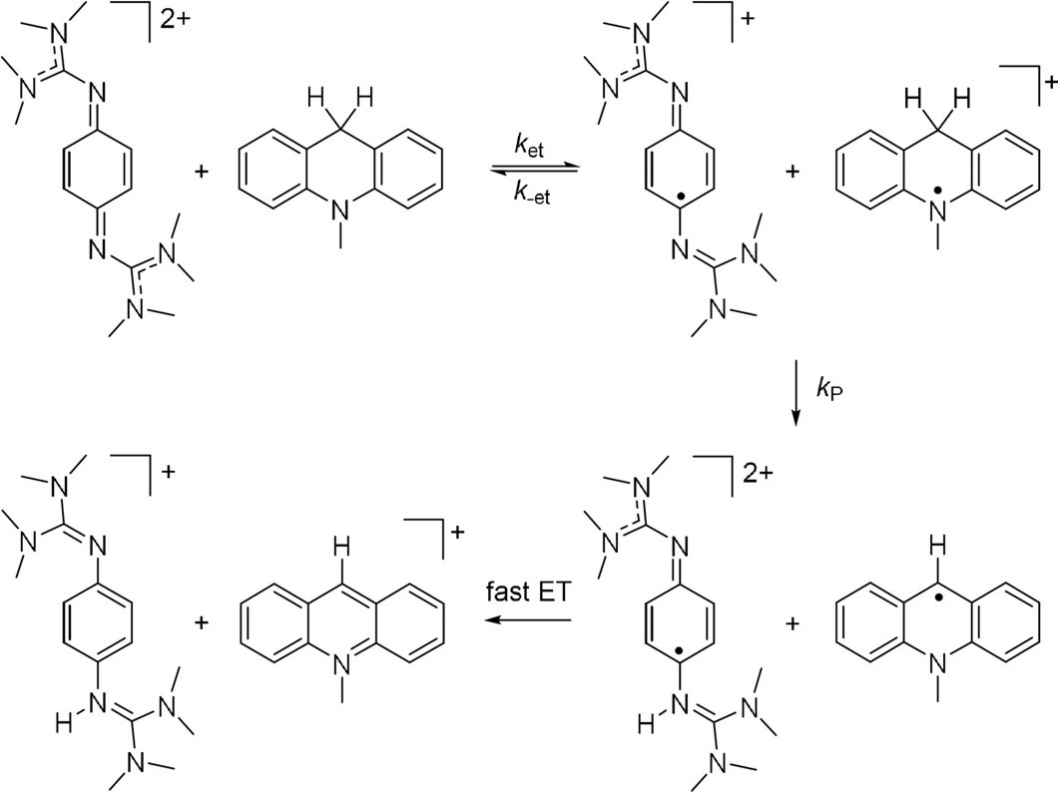
Suggested mechanism for the oxidation of *N*‐methylacridane with **2** ^2+^.

The large KIE clearly shows that the rate constant for the protonation step contributes to the overall rate constant. AcrH_2_ is known to prefer a stepwise e^−^, H^+^, e^−^ pathway for hydride transfer in many reactions. The energy for electron transfer, Δ*G*_el_, for reaction between **2** ^2+^ and AcrH_2_^+^ of 0.73 V (70 kJ mol^−1^) is well within the region of conventional electron transfer under standard conditions, supporting electron transfer in the first step. Therefore a stepwise e^−^, H^+^, e^−^ pathway, as sketched in Scheme 12, is likely to be operative here, too. The last step of the proposed mechanism is a fast second electron transfer, profiting from the low potential of the AcrH^.^ radical (*E*_ox_=−0.46 V vs. SCE, ca. −0.06 V vs. Fc^+^/Fc).^[43]^ Hence, this last step plays no role in the overall rate. According to the formula *k*_H_=*k*_P_ *k*_et_/(*k*_−et_+*k*_P_),^[42]^ the observation of a large KIE value means that *k*_P_ (the rate constant for proton transfer) is much smaller than *k*_−et_ (the rate constant for back electron‐transfer regenerating the reactants).

**Figure 4:**
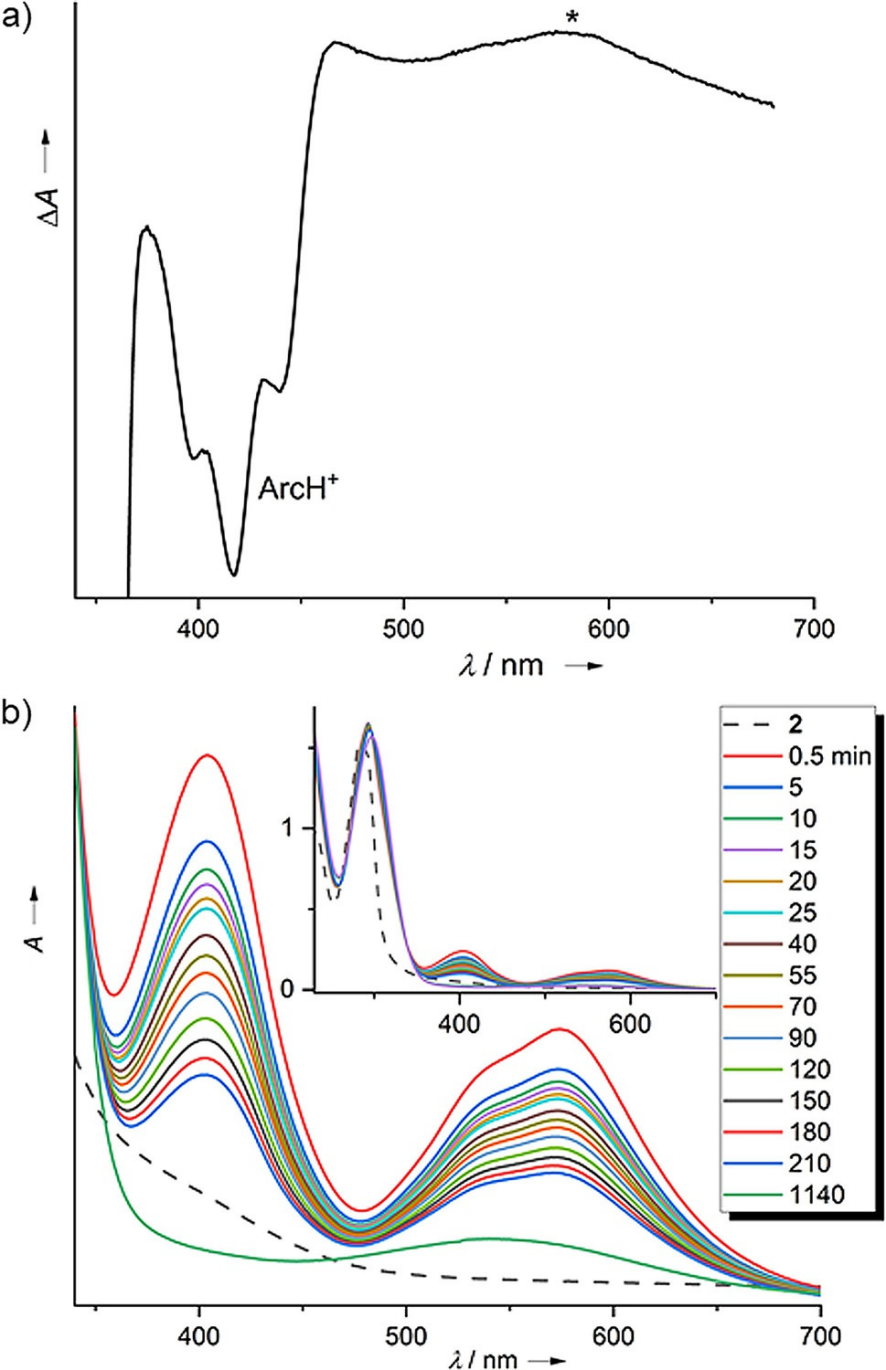
a) Difference spectrum (26 minus 78 min reaction time) for the reaction between **2**(PF_6_)_2_ and AcrH_2_ (10 equivalents) in CH_3_CN solution. The band assigned to **2** ^⋅+^ is highlighted by an asterisk. b) Decay of the absorptions due to the radical monocation **2** ^⋅+^, formed instantly upon mixing **2** and **2** ^2+^ in acetonitrile solution.

In the UV/Vis experiment, a small and broad absorption around 575 nm first increased in intensity, reaching a maximum after ca. 26 min, and then decreased (see difference spectrum in Figure 4 a), belonging to a reaction intermediate. According to the proposed reaction scheme, the obvious candidates for this intermediate are the radicals **2** ^.+^ and AcrH_2_^.+^, formed upon first electron transfer. A broad, weak absorption around 640 nm was previously assigned to the radical cation AcrH_2_^.+^.[^42^, ^43^, ^44^] We did not observe this band, likely because the concentrations of AcrH_2_^.+^ were too low. To obtain more information on the other candidate, **2** ^.+^, we prepared a 1:1 mixture of **2** (colourless) and **2**(PF_6_)_2_ (yellow) in CH_3_CN solution. The reaction turned instantly deep‐purple. In the UV/Vis spectrum, bands at 575 nm (with a shoulder at 542 nm) and 404 nm appeared (Figure 4 b), vanishing within a few hours. TD‐DFT calculations (B3LYP/def2‐TZVP) predicted electronic transitions at 543/501 and 365/346 nm for **2** ^.+^, in good agreement with the experimental values (see the Supporting Information for details). The band at 575 nm could therefore be assigned to the radical **2** ^.+^. The other band of **2** ^.+^ in the visible region at 404 nm is obscured by the strong, broad bands of AcrH^+^ in this region (Figure 4 a). The detection of **2** ^.+^ as a reaction intermediate is valuable additional evidence for the validity of the reaction pathway sketched in Scheme 11.

### Reaction between two bisguanidines

**Figure 5:**
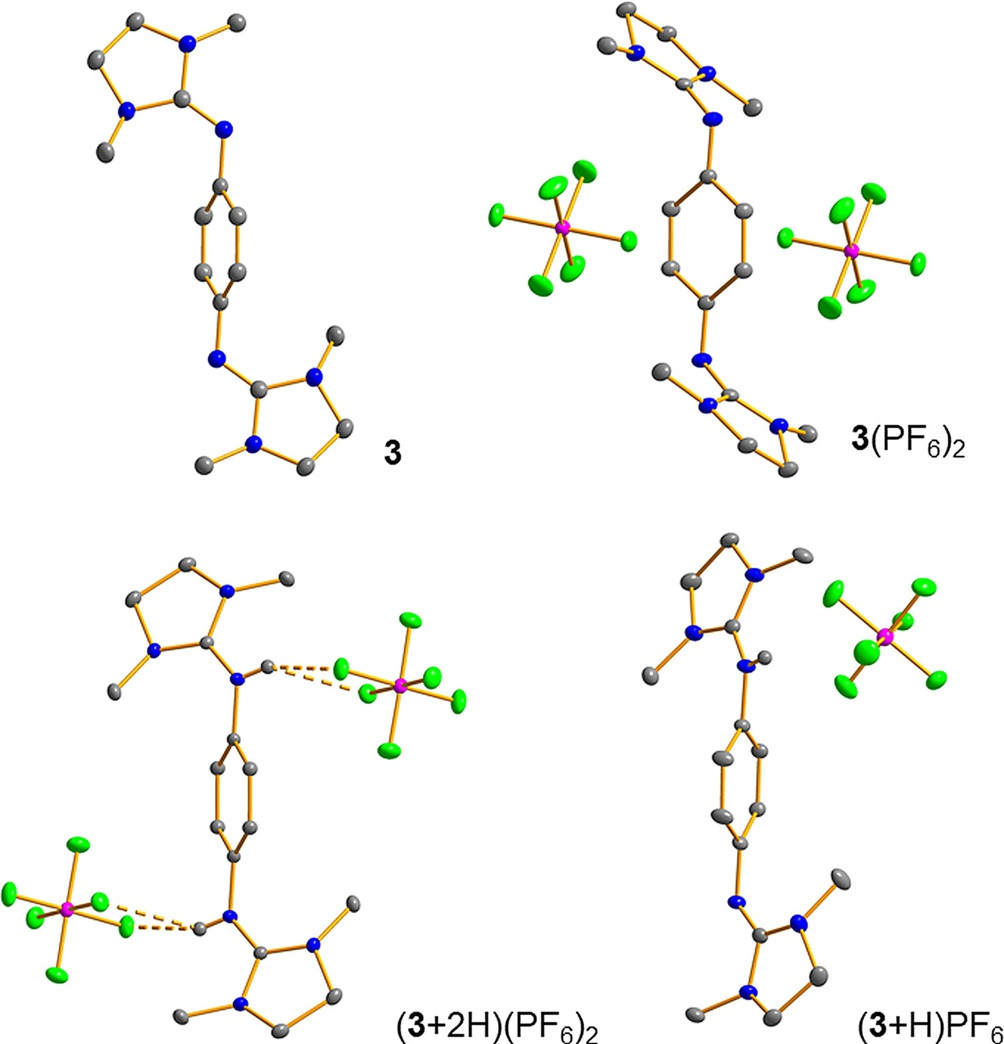
Visualisation of the solid‐state structures of the stable protonation and redox states of **3**. C−H protons omitted. Displacement ellipsoids drawn at the 50 % probability level. Colour code: N blue, C grey, P pink, F green. See the Supporting Information for details.

To test PCET between closely related redox‐active guanidines, we synthesized the new bisguanidine **3** (77 % yield) by reaction of *p*‐phenylenediamine‐dihydrochloride with 2‐chloro‐1,3‐dimethyl‐4,5‐dihydro‐1*H*‐imidazolium chloride (prepared in situ from 1,3‐dimethyl‐2‐imidazolidinone and oxalyl chloride, see the Supporting Information for details). Compound **3** was then oxidized with FcPF_6_ to give the salt **3**(PF_6_)_2_ (48 % isolated yield), and protonated with NH_4_PF_6_ to give (**3**+2 H)(PF_6_)_2_ (72 % isolated yield), and these two states were completely characterized. In addition, (**3**+H)PF_6_ was synthesized from a 1:1 mixture of **3** and (**3**+2 H)(PF_6_)_2_. The structures in the solid state of all relevant states are illustrated in Figure 5. Further experiments showed that **3** ^2+^ does not degrade in the presence of larger amounts of a strong acid, in contrast to **2** ^2+^.

**Figure 6:**
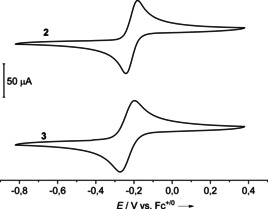
CV curves (CH_3_CN, Ag/AgCl reference electrode, 0.1 m N(*n*Bu)_4_(PF_6_) as supporting electrolyte, scan speed 100 mV s^−1^) for the two compounds **2** and **3** measured in oxidation direction. Potentials measured vs. Fc^+^/Fc.

According to cyclic voltammetry (Figure 6), the redox properties of **3** are very similar to those of **2**. In both cases, two‐electron waves are observed, leading reversibly to the dicationic state. An *E*_1/2_ value of −0.21 V vs. Fc^+^/Fc (*E*_ox_=−0.18 V, *E*_red_=−0.24 V) for **2** compares with an *E*_1/2_ value of −0.24 V vs. Fc^+^/Fc (*E*_ox_=−0.20 V, *E*_red_=−0.27 V) for **3**. Although the radical cation **3^⋅^** ^+^ does not appear in the CV measurements, it could be generated (similar to **2** ^.+^) by mixing neutral **3** and **3**(PF_6_)_2_ in CH_3_CN solutions or by titration of solutions of **3** with **3**(PF_6_)_2_, and displays strong absorptions at 566 and 383 nm (see the Supporting Information). Interestingly, degradation of **3^⋅^** ^+^ is much slower than that of **2^⋅^** ^+^ (see the Supporting Information).

**Scheme 13:**
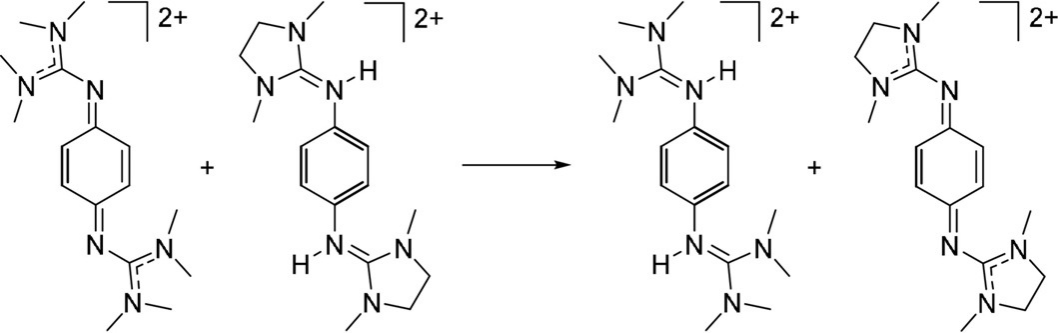
Oxidation of (**3**+2H)^2+^. Reagents and conditions: acetonitrile, **2**(PF_6_)_2_ (1 equiv), 45 min at 55 °C, 99 % yield.

We then directly compared the PCET reactivity of the two compounds. Reaction between **2** ^2+^ and (**3**+2H)^2+^ gave almost quantitative yields of **3** ^2+^. For example, reaction at 55 °C for a period of 45 min gave 99 % yield (Scheme 13). On the other hand, no reaction was observed when **3** ^2+^ and (**2**+2H)^2+^ were mixed together. These results are in line with quantum chemical calculations (B3LYP/def2‐TZVP), predicting the reaction (at *ϵ*_r_=1) in Scheme 12 to exhibit a reaction enthalpy at 0 K of −66.7 kJ mol^−1^ and a Gibbs free energy at 298 K of −58.2 kJ mol^−1^.

**Figure 7:**
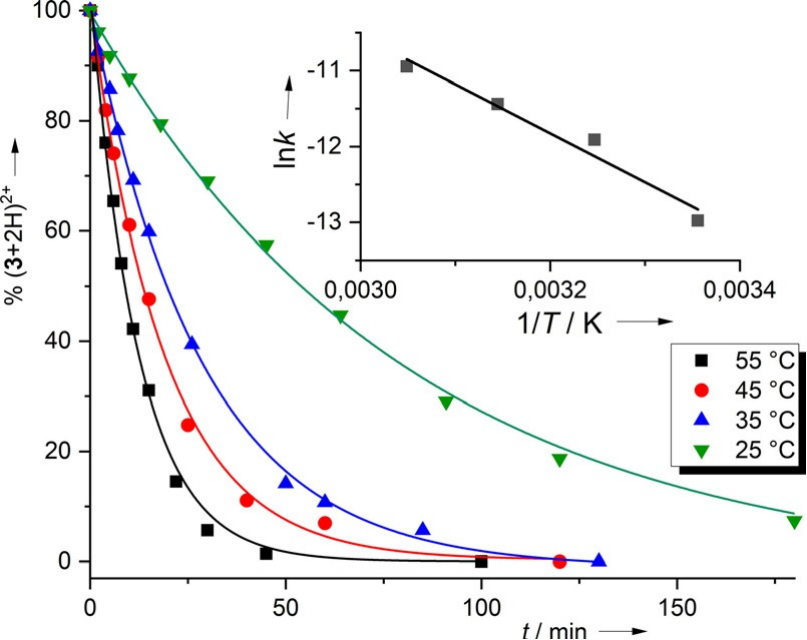
Conversion vs. time plots for the reaction between **2** ^2+^ and (**3**+2H)^2+^ at different temperatures. The second‐order *k* values estimated from each curve were used in the ln *k* vs. 1/*T* plot in the inlet, allowing the Arrhenius activation energy to be estimated.

From the temperature dependence of the conversion versus time plots and assuming a second‐order rate law, the activation energy *E*_A_ could be estimated from an Arrhenius plot (see Figure 7 and the Supporting Information for details). An *E*_A_ value of 54±8 kJ mol^−1^ was obtained in this way. This quite low activation energy might argue for a concerted e^−^, H^+^ transfer; a stepwise e^−^, H^+^ transfer would create a highly unfavourable intermediate (**3**+2H)^3+^, and a stepwise H^+^, e^−^ transfer a similarly unfavourable intermediate (**2**+H)^3+^. The basicity of GFAs with tetramethylguanidino groups is significantly higher than that of GFAs with *N*,*N*′‐dimethylethylene‐guanidino groups.^[45]^ Thus, the redox potential and the p*K*_a_ value, being the two decisive factors for the PCET thermodynamics, seem to operate in the same direction, making **3** ^2+^ a weaker PCET reagent than **2** ^2+^. Hence, the levelling effect observed for quinones, which results from opposite trends of potential and p*K*_a_ value, might not occur for redox‐active guanidines.

## Conclusions

Proton‐coupled electron transfer (PCET) reactions between redox‐active guanidines and a variety of organic compounds in which C−H bonds are cleaved were systematically evaluated. First it is demonstrated that substrates with oxidation potentials up to at least 1.2 V vs. Fc^+/0^ could be oxidized in the presence of a strong acid. Then, the two redox‐active guanidines 1,2,4,5‐tetrakis(tetramethylguanidino)‐benzene (**1**) and 1,4‐bis(tetramethylguanidino)‐benzene (**2**), both applied in their oxidized, dicationic state (**1** ^2+^ or **2** ^2+^) are compared in their PCET reactivity. In the course of our analysis, the reactions with the three substrates 1‐benzyl‐1,4‐dihydronicotinamide (BNAH), 10‐methyl‐9,10‐dihydroacridane (AcrH_2_), and 9,10‐dihydroanthracene (AnH_2_) were studied for both **1** ^2+^ and **2** ^2+^. The results clearly show that **2** ^2+^ is a significantly stronger oxidant in PCET reactions. However, in contrast to **1** ^2+^, it degrades in the presence of larger quantities of a strong acid. In all PCET reactions discussed in this work, slow proton transfer is likely to proceed from an initial electron‐transfer equilibrium. Consequently, a relatively large kinetic isotope effect (KIE) of 5.6 was obtained for the reaction between **2** ^2+^ and AcrH_2_.

Finally, we synthesized a new redox‐active guanidine, 1,4‐bis(*N*,*N*′‐dimethylethylene‐guanidino)‐benzene (**3**) and compared the PCET reactivity of **3** ^2+^ and **2** ^2+^. Compound **3** ^2+^ has a slightly lower reduction potential and is a weaker oxidant in PCET reactions. The comparison between the two closely related bisguanidines indicates that a “levelling effect” (resulting from opposite trends of potential and p*K*_a_) as observed for quinones, might not be an issue for redox‐active guanidines, allowing the tuning of the PCET thermodynamics by derivatisations. However, more work is necessary to confirm this assumption, which would be very helpful for a directed approach to PCET reactions.

The results presented in this work clearly show that redox‐active guanidines are potent PCET reagents and real alternatives to toxic quinones such as chloranil or DDQ.

## Experimental Section

**Crystallographic data**: Deposition numbers 2013656, 2013653, 2013654, 2013652, 2013655, and 2013657 (**1**(ClO_4_)_2_, (**2**+2 H)(PF_6_)_2_, **3**, **3**(PF_6_)_2_, (**3**+H)(PF_6_), and (**3**+2 H)(PF_6_)_2_) contain the supplementary crystallographic data for this paper. These data are provided free of charge by the joint Cambridge Crystallographic Data Centre and Fachinformationszentrum Karlsruhe Access Structures service.

## Conflict of interest

The authors declare no conflict of interest.

## Supporting information

As a service to our authors and readers, this journal provides supporting information supplied by the authors. Such materials are peer reviewed and may be re‐organized for online delivery, but are not copy‐edited or typeset. Technical support issues arising from supporting information (other than missing files) should be addressed to the authors.

Supplementary
