# Supplementary material for: Evaluation of the Synthetic Scope and the Reaction Pathways of Proton‐Coupled Electron Transfer with Redox‐Active Guanidines in C−H Activation Processes
Source: Chemistry. 2020 Nov 3;26(69):16504–13. doi: 10.1002/chem.202003424 (PMC7756729; doi:10.1002/chem.202003424)

Chemistry–A European Journal

Supporting Information

Evaluation of the Synthetic Scope and the Reaction Pathways of Proton-Coupled Electron Transfer with Redox-Active Guanidines in C–H Activation Processes

Ute Wild, Petra Walter, Olaf Hübner, Elisabeth Kaifer, and Hans-Jörg Himmel*[a]

Content

No.	Title	Page
1	General information	2
2	Synthesis of new compounds	4
3	Analytical data for new compounds	7
4	Experimental details for oxidative coupling of *N*-ethylcarbazole to *N,N'*-diethyl-3,3'-bicarbazole	18
5	Experimental details for oxidative coupling of 3,3''-dimethoxy-3',4'-dimethyl-*o*-terphenyl to 3,10-dimethoxy-6,7-dimethyltriphenylen	20
6	Experimental details for reaction of **1**$(PF_6)_2$ with 10-methyl-9,10-dihydroacridine ($AcrH_2$)	22
7	Experimental details for reaction of **1**$(PF_6)_2$ with 10-methyl-[9,9'-$^2H_2$]-acridine ($AcrD_2$)	26
8	Experimental details for reaction of $\mathbf{1}^{2+}$ with 9,10-dihydroanthracene (AnH_2)	31
9	Experimental details for reaction of **1**$(PF_6)_2$ with 1-benzyl-1,4-dihydronicotinamide (BNAH)	34
10	Experimental details for reaction of **2**$(PF_6)_2$ with (**1**+2H)$(PF_6)_2$	37
11	Experimental details for reaction of **2**$(PF_6)_2$ with 1-benzyl-1,4-dihydronicotinamide (BNAH)	44
12	Experimental details for reaction of **2**$(PF_6)_2$ with 10-methyl-9,10-dihydroacridine ($AcrH_2$)	46
13	Experimental details for reaction of **2**$(PF_6)_2$ with 10-methyl-[9,9'-$^2H_2$]-acridine ($AcrD_2$)	49
14	Experimental details for reaction of **2**$(PF_6)_2$ with 9,10-dihydroanthracene (AnH_2)	52
15	Experimental details for reaction of **2**$(PF_6)_2$ with 3,3",4,4"-tetramethoxy-*o*-terphenyl (TMTP) to 2,3,10,11-tetramethoxy-triphenylene	56
16	Experimental details for reaction of 2$(PF_6)_2$ with *p*-dihydro-benzoquinone to *p*-benzoquinone	61
17	Reaction of **2**$(PF_6)_2$ with $AcrH_2$: UV-vis experiments	66
18	Reaction of **2**$(PF_6)_2$ with $AcrD_2$: UV-vis experiments	69
19	Experimental details for reaction of **2**$(PF_6)_2$ with (**3**+2H)$(PF_6)_2$	71
20	Details for the recycling of **1**$(PF_6)_2$	81
21	Details of the quantum-chemical calculations	82

1 General information

All synthetic work was carried out using standard Schlenk techniques under argon atmosphere. Solvents were dried with an MBraun MB-SPS-800 Solvent Purification System and stored over molecular sieves.

The following chemicals were purchased and used as delivered: hexamethylbenzene (99 %, abcr), $HBF_4{\cdot}OEt_2$ (Sigma-Aldrich), *N*-ethylcarbazole (97 %, Sigma-Aldrich), $AgClO_4$ (99 %, abcr), 10-methylacridone (98 %, TCI), $LiAlD_4$ (98 %, strem chemicals), 1-benzyl-1,4-dihydronicotinamide (>95 %, TCI), 9,10 dihydroanthracene (97 %, Sigma-Aldrich), NH_4PF_6 (99 %, acros), ferrocenium hexafluorophosphate (technical grade, Sigma-Aldrich), 1,4-diaminobenzene dihydrochloride (>99%, Sigma-Adrich). The synthesis of 3,3'',4,4''-tetramethoxy-*o*-terphenyl, 3,3''-dimethoxy-3',4'-dimethyl-*o*-terphenyl [1] **1**$(BF_4)_2$,[2] **1**$(PF_6)_2$,[3] **2** and **2**$(PF_6)_2$[4] followed the literature procedures. Elemental analyses were carried out at the Microanalytical Laboratory of the University of Heidelberg. NMR spectra were recorded on a Bruker DPX 200, Bruker DRX 200, Bruker Avance II 400 or Bruker AVANCE III 600 system. Solvent resonances were taken as references for all ^1H NMR spectra. UV-vis spectra were recorded with a Cary 5000 spectrophotometer and cyclic voltammetry (CV) measurements relied on a Metrohm Autolab potentiostate PGSTAT204, $(^nBu_4N)(PF_6)$ (electrochemical grade (>99.0), Fluka) was employed as supporting electrolyte. Stopped flow measurements were made with an "Applied Photophysics SX 18MV-R". MS-ESI measurements relied on a Bruker microTOF II ESI mass spectrometer.

X-ray crystallography

Suitable crystals for single-crystal structure determination were taken directly from the mother liquor, taken up in perfluorinated polyether oil and fixed on a cryo loop. Full shells of intensity data were collected at low temperature with a Nonius Kappa CCD diffractometer (Mo-K_α radiation, sealed X-ray tube, graphite monochromator) for compound (**2**+2H)$(PF_6)_2$, and Bruker D8 Venture, dual source (Mo- or Cu-K_α radiation, microfocus X-ray tube, Photon III detector) for all other compounds. Data were processed with the standard Nonius and Bruker (SAINT, APEX3) software package.[5]

[1] Linyi Zhai, Ruchi Shukla, Shirya H. Wadumethrige, Rajendra Rathore, *J. Org. Chem.* **2010**, *75*, 4748–4760.
[2] U. Wild, O. Hübner, L. Greb, M. Enders, E. Kaifer, H.-J. Himmel, *Eur. J. Org. Chem.* **2018**, 5910–5915.
[3] U. Wild, S. Federle, A. Wagner, E. Kaifer, H.-J. Himmel, *Chem. Eur. J.* **2016**, *22*, 11971–11976.
[4] J. Hornung, O. Hübner, E. Kaifer, H.-J. Himmel*, *RSC Advances* **2016**, *6*, 39323-39329
[5] a) *DENZO-SMN*, Z. Otwinowski & W. Minor, Processing of X-ray Diffraction Data Collected in Oscillation Mode, Methods Enzymol. 1997, 276, Eds C. W. Carter, R. M. Sweet, Academic Press.; b) *SAINT*, Bruker AXS GmbH, Karlsruhe, Germany **2016.**

Multiscan absorption correction was applied using the SADABS program.[6] The structures were solved by intrinsic phasing[7] and refined using the SHELXTL software package (Version 2014/6 and 2018/3).[8] Graphical handling of the structural data during solution and refinement were performed with OLEX2.[9] All non-hydrogen atoms were given anisotropic displacement parameters. Hydrogen atoms bound to carbon were input at calculated positions and refined with a riding model. Hydrogen atoms bound to nitrogen were located in difference Fourier syntheses and refined, either fully or with appropriate distance and/or symmetry. Crystallographic data for the structures reported in this paper have been deposited in the Cambridge Crystallographic Data Centre (CCDC No. 2013656 for **1**(ClO_4)$_2$, 2013653 for (**2**+2H)(PF_6)$_2$, 2013654 for **3**, 2013652 for **3**(PF_6)$_2$, 2013655 for (**3**+H)(PF_6), 2013657 for (**3**+2H)(PF_6)$_2$. These data can be obtained free of charge from The Cambridge Crystallographic Data Centre via www.ccdc.cam.ac.uk/data_request/cif.

---

[6] a) G. M. Sheldrick, SADABS, Bruker AXS GmbH, Karlsruhe, Germany **2004-2014**; b) L. Krause, R. Herbst-Irmer, G. M. Sheldrick, D. Stalke, J. *Appl. Cryst*. **2015**, *48*, 3.

[7] a) G. M. Sheldrick, SHELXT, *Program for Crystal Structure Solution*, University of Göttingen, Germany **2014-2018**; b) G. M. Sheldrick, *Acta Cryst*. **2015**, *A71*, 3.

[8] a) G. M. Sheldrick, *SHELXL-20xx*, University of Göttingen and Bruker AXS GmbH, Karlsruhe,Germany **2012-2018**; b) W. Robinson, G. M. Sheldrick in: N. W. Isaaks, M. R. Taylor (eds.) „*Crystallographic Computing 4*“, Ch. 22, IUCr and Oxford University Press, Oxford, UK, **1988**; c) G. M. Sheldrick, *Acta Cryst*. **2008**, *A64*, 112; (d) G. M. Sheldrick, *Acta Cryst*. **2015**, *C71*, 3.

[9] O. V. Dolomanov, L. J. Bourhis, R. J. Gildea, J. A. K. Howard, H. Puschmann, OLEX2: A complete structure solution, refinement and analysis program, *J. Appl. Cryst.* **2009**, *42*, 339.

2 Synthesis of new compounds

Compound 1$(ClO_4)_2$

130 mg (0.245 mmol) of **1** and 99 mg (0.478 mmol) of $AgClO_4$ were dissolved in 20 ml CH_3CN and the reaction mixture stirred for a period of 45 min at room temperature. Then the reaction is filtrated and the solvent removed in vacuo. The solid residue is washed several times with Et_2O. For further purification the CH_3CN solution was layered by Et_2O. The microcrystalline precipitate, formed over a period of several days, was washed with Et_2O and dried in vacuo to give 150 mg (0.206 mmol, 86% yield) of **1**$(ClO_4)_2$. Elemental analysis calcd. (%) for $C_{26}H_{50}N_{12}Cl_2O_8$ (729.66 g·mol^{-1}): C 42.80, H 6.91, N 23.04; found C 42.64, H 6.97, N 23.76. ^{1}H NMR (600.13 MHz, CD_3CN, 295.0 K): δ = 5.15 (s, 2 H, C*H*), 2.88 (s, 48 H, CH_3) ppm. ^{13}C NMR (150.92 MHz, CD_3CN, 295.1 K): δ = 167.62, 157.26 (C_q), 103.46 (*C*H), 40.96 (*C*H_3) ppm. MS (ESI, CH_3CN): m/z (%) = 265 (100) [**1**]$^{2+}$, 244 (60) [**1**H-N$(CH_3)_2$]$^{2+}$, 629 (39) [**1**(ClO_4)]$^+$. Crystal data: C_{26} H_{50} Cl_2 N_{12} O_8: *M*r = 729.68, 0.12 × 0.113 × 0.107 mm^{-3}, monoclinic, space group P2(1)/n, a = 7.8129(17), b = 17.529(2), c = 13.203(2) Å, β = 93.468(9), V = 1804.9(6) Å^3, Z = 2, d_{calc} = 1.343 g cm^{-3}, Mo-K_α radiation (λ = 0.71073 Å), T = 100 K, θ_{range} 1.933° to 28.495°, Reflections measd. 45272, indep. 4581, R_{int} = 0.0583, Final R indices [$I > 2\sigma(I)$]: R_1 = 0.0429, wR_2 = 0.1091.

NMR experiment: 1 + (1+2H)$(PF_6)_2$

In an NMR tube, compound **1** (3.588 mg, 6.76·10^{-3} mol) and (**1**+2H)$(PF_6)_2$ [1] (5.400 mg, 6.56·10^{-3} mol) were dissolved in 0.45 ml CD_3CN. After 10 min at room temperature, the solution was studied by NMR spectroscopy. ^{1}H NMR (399.89 MHz, CD_3CN, 294.6 K): δ = 5.84 (s, 2 H, CH_{arom}), 2.70 (s, 48 H, CH_3) ppm.

Compound (2+2H)$(PF_6)_2$

A solution of ammonium hexafluorophosphate (158 mg, 0.96 mmol) in CH_3CN (4 ml) is added to a solution of compound **2** (152 mg, 0.50 mmol) in CH_3CN (3 ml). The reaction mixture is stirred for 1 h at 50 °C and additional 18 h at room temperature. The solvent is removed in vacuo and the solid residue re-dissolved in CH_3CN (3 ml). After addition of charcoal the solution is filtered over celite. The solution is concentrated by removal of half of the solvent and then layered with diethyl ether. Compound (**2**+2H)$(PF_6)_2$ is obtained as colorless crystals in a yield of 196 mg (0.33 mmol, 68%), that are structurally characterized by single-crystal X-ray diffraction. Elemental analysis calcd. (%) for $C_{16}H_{30}N_6F_{12}P_2$ (596.38 g·mol^{-1}): C 32.22, H 5.07, N 14.09, found C 32.43, H 5.37, N 14.13. ^{1}H NMR (399.89 MHz, CD_3CN, 295.4 K): δ = 7.08 (m, 4 H, CH_{arom}), 2.95 (s, 24 H, CH_3) 7.96 ppm (2 H, NH). ^{13}C NMR (100.56 MHz, CD_3CN, 295.9 K): δ = 158.87, 135.27, 122.88, 40.38 ppm.

---

[1] A. Peters, E. Kaifer, H.-J. Himmel, *Eur. J. Org. Chem.* **2008**, 5907–5914

NMR experiment: 2 + (2+2H)(PF_6)$_2$

In an NMR tube, compound **2** (0.780 mg, $2.56 \cdot 10^{-6}$ mol) and (**2**+2H)(PF_6)$_2$ (1.536 mg, $2.57 \cdot 10^{-6}$ mol) were dissolved in 0.5 ml CD_3CN. After 10 min at room temperature, the solution was studied by NMR spectroscopy. Attempts to obtain crystals by concentrating the solution to 0.1 ml, layering the solution with diethyl ether and storing it at −21 °C failed. ^1H NMR (399.89 MHz, CD_3CN, 295.3 K): δ = 6.61 (m, 4 H, CH_{arom}), 2.70 ppm (s, 24 H, CH_3).

1,4-Bis-(*N*,*N'*-dimethylethyleneguanidino)-benzene, 3

1,3-dimethyl-2-imidazolidinone (2.9 mL, 25 mmol) is dissolved in chloroform (50 ml) oxalylchloride (10.75 ml, 135 mmol) added while stirring. Subsequently, the reaction mixture is heated to reflux for 18 h. Then, the solvent is removed in vacuo and the remaining solid washed with diethyl ether (2 times with portions of 20 ml). The resulting chloroformamidinium chloride is dissolved in acetonitrile (20 ml). This solution is added to a suspension of *p*-phenylenendiamine·2HCl (1.81 g, 10 mmol) and triethylamine (10 ml, 71.25 mmol) in acetonitrile (30 ml) at -5 °C. After completion of the addition, the reaction mixture is stirred for 5 h at 0 °C. The solvent is removed in vacuo and the remaining solid re-dissolved in aqueous HCl (5%). Once all of the residue is solved, aqueous NaOH (40%) is added. The resulting mixture is extracted with dichloromethane. The organic phases are dried over K_2CO_3 and the solvent is removed in vacuo. The crude product was re-crystallized from hot acetonitrile. Compound **3** is obtained as a beige solid in a yield of 2.32 g (7.72 mmol, 77.2%). Crystals suitable for structural characterization by single crystal X-ray diffraction are grown from acetonitrile layered by petroleum ether. Elemental analysis calcd. (%) for $C_{16}H_{24}N_6$ (300.32 g·mol^{-1}): C 63.97, H 8.05, N 27.98; found C 63.82, H 7.76, N 28.01. ^1H NMR (399.89 MHz, CD_3CN, 295.4 K): δ = 6.56 (m, 4 H, CH_{arom}), 3.19 (s, 8 H, CH_2), 2.54 ppm (s, 12 H, CH_3). ^{13}C NMR (100.56 MHz, CD_3CN, 295.9 K): δ = 155.73, 143.88, 122.65, 48.90, 35.24 ppm.

Compound 3(PF_6)$_2$

Ferrocenium hexafluorophosphate (214 mg, 0.62 mmol) and compound **3** (102 mg, 0.339 mmol) are dissolved in 12 ml dry dichloromethane. The reaction mixture is stirred at room temperature for 3h. The solvent is removed in vacuo and the yellow-brown residue washed three times with diethyl ether. The residue is re-dissolved in acetonitrile and layered with diethylether to obtain 90 mg (0.15 mmol, 48%) of an orange crystalline solid as product. Elemental analysis calcd. (%) for $C_{16}H_{24}N_6F_{12}P_2$ (590.35 g·mol^{-1}): C 32.55, H 4.10, N 14.24; found C 32.23, H 4.35, N 14.17.
^1H NMR (399.89 MHz, CD_3CN, 294.8 K): δ = 7.17 (m, 4 H, CH_{arom}), 3.94 (s, 8 H, CH_2), 2.83 ppm (s, 12 H, CH_3). ^{13}C NMR (100.56 MHz, CD_3CN, 295.9 K): δ = 166.07, 162.39, 49.96, 32.82 ppm.

(3+2H)(PF_6)$_2$

A solution of ammonium hexafluorophosphate (140 mg, 0.85 mmol) in CH_3CN (2 ml) is added to a solution of compound **3** (144 mg, 0.48 mmol) in CH_3CN (3 ml). The reaction mixture is stirred for 1 h at 50 °C and additional 18 h at room temperature. The solvent is removed in vacuo and the solid residue re-dissolved in CH_3CN (3 ml). After addition of charcoal the solution is filtered over celite. The solution is concentrated by removal of Half of the solvent and then layered with diethyl ether. One obtains (**3**+2H)(PF_6)$_2$ as colorless crystals in a yield of 205 mg (0.34 mmol, 72%), that are structurally characterized by single-crystal X-ray diffraction. Elemental analysis calcd. (%) for $C_{16}H_{26}N_6F_{12}P_2$ (592.35 g·mol^{-1}): C 32.44, H 4.42, N 14.19; found C 32.56, H 4.76, N 14.26. ^{1}H NMR (399.89 MHz, CD_3CN, 295.3 K): δ = 7.21 (m, 4 H, CH_{arom}), 3.72 (s, 8 H; CH_2), 2.78 (s, 12 H; CH_3) 7.92 ppm (2 H; NH). ^{13}C NMR (100.56 MHz, CD_3CN, 295.9 K): δ = 157.11, 134.52, 124.88, 49.47, 34.70 ppm.

NMR experiment: 3 + (3+2H)(PF_6)$_2$

In an NMR tube, compound **3** (0.764 mg, 2.54·10^{-6} mol) and (**3**+2H)(PF_6)$_2$ (1.504 mg, 2.54·10^{-6} mol) are dissolved in 0.5 ml CD_3CN. After 10 min at room temperature the solution was studied by NMR spectroscopy. Then the solution is concentrated to 0.1 ml and the solution layered with diethyl ether. One obtains (**3**+H)(PF_6) as colorless crystals that are structurally characterized by single-crystal X-ray diffraction.
^{1}H NMR (399.89 MHz, CD_3CN, 295.3 K): δ = 6.89 (m, 4 H, CH_{arom}), 3.46 (s, 8 H, CH_2), 2.66 (s, 12 H, CH_3) 7.92 ppm (2 H, NH).

3 Analytical data for new compounds

NMR spectra

^{1}H NMR spectrum (399.89 MHz, CD_3CN, 295.4 K) for compound (**2**+2H)$(PF_6)_2$

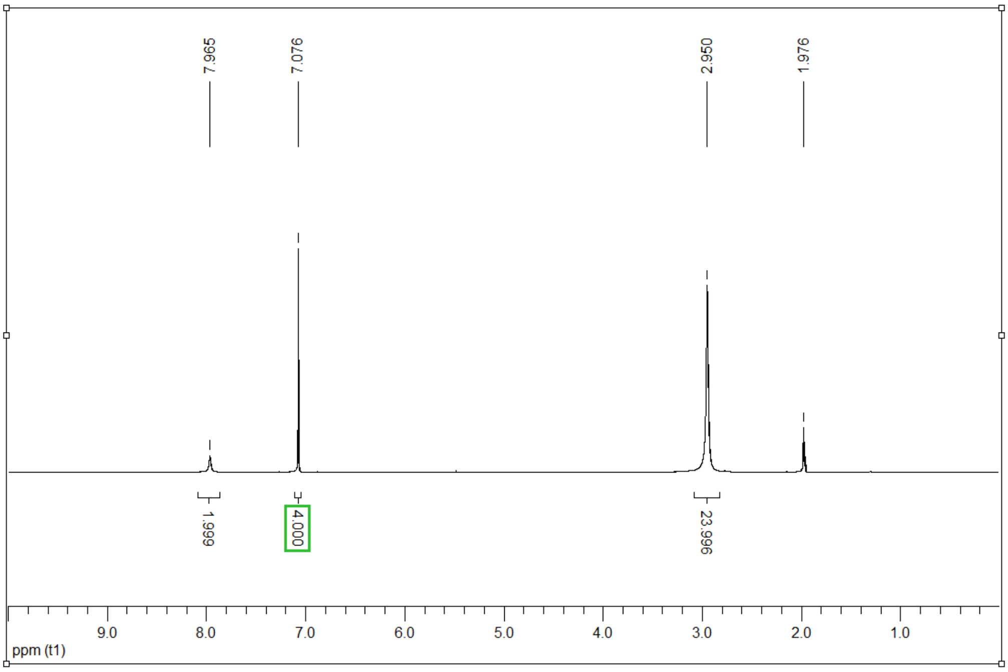

^{13}C NMR spectrum (100.56 MHz, CD_3CN, 295.9 K) for compound (**2**+2H)$(PF_6)_2$

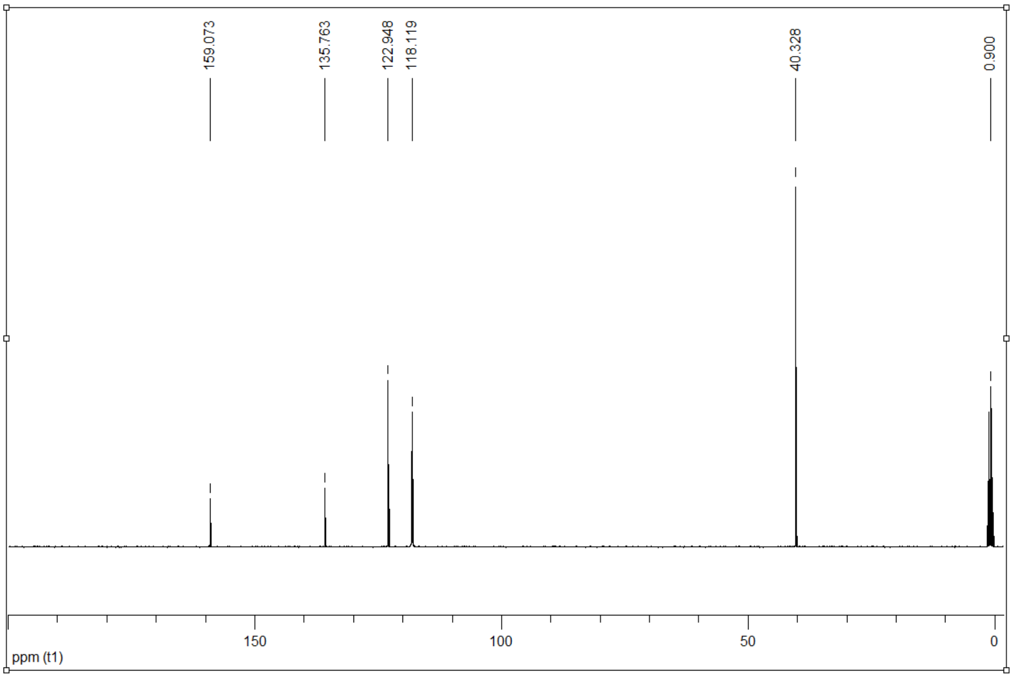

^{1}H NMR spectrum (399.89 MHz, CD_3CN, 295.4 K) of compound **3**

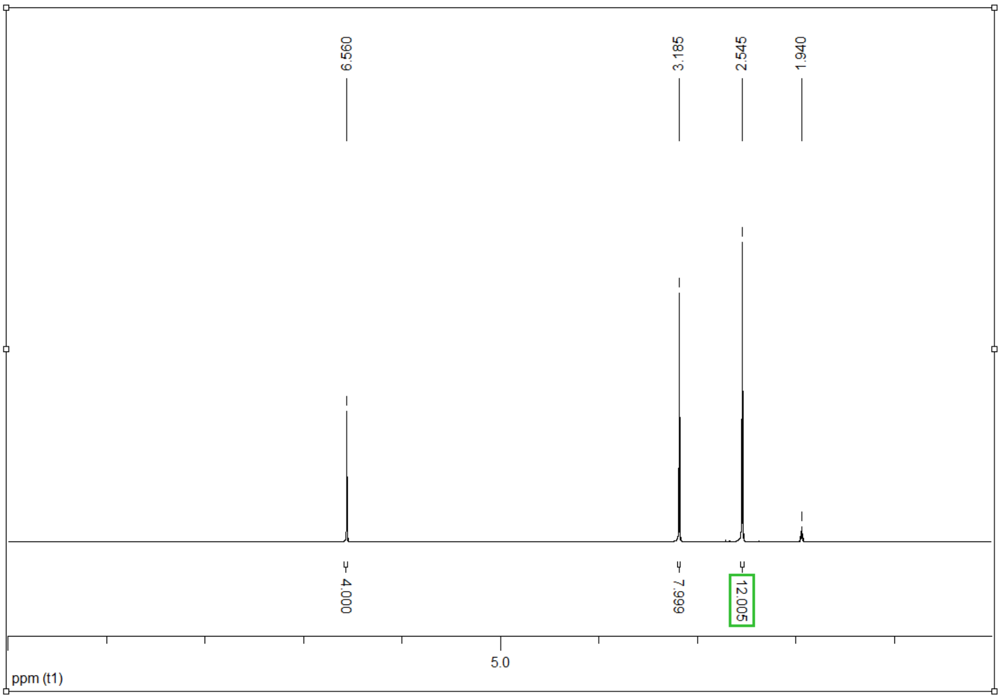

^{13}C NMR spectrum (100.56 MHz, CD_3CN, 295.9 K) of compound **3**

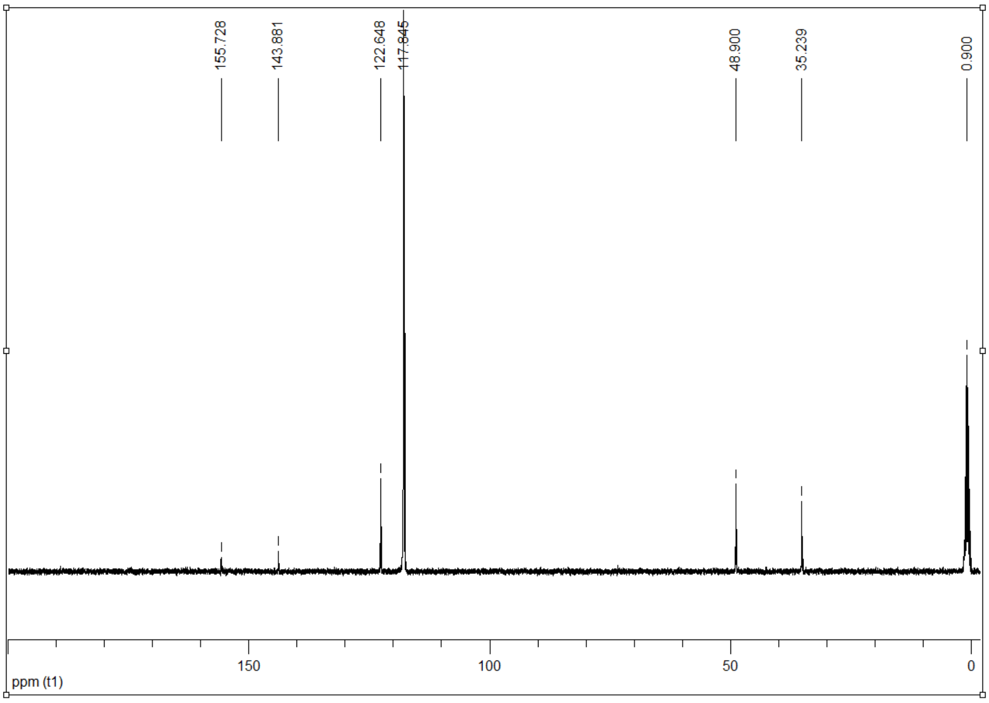

^1H NMR spectrum (399.89 MHz, CD_3CN, 294.8 K) of compound **3**$(PF_6)_2$

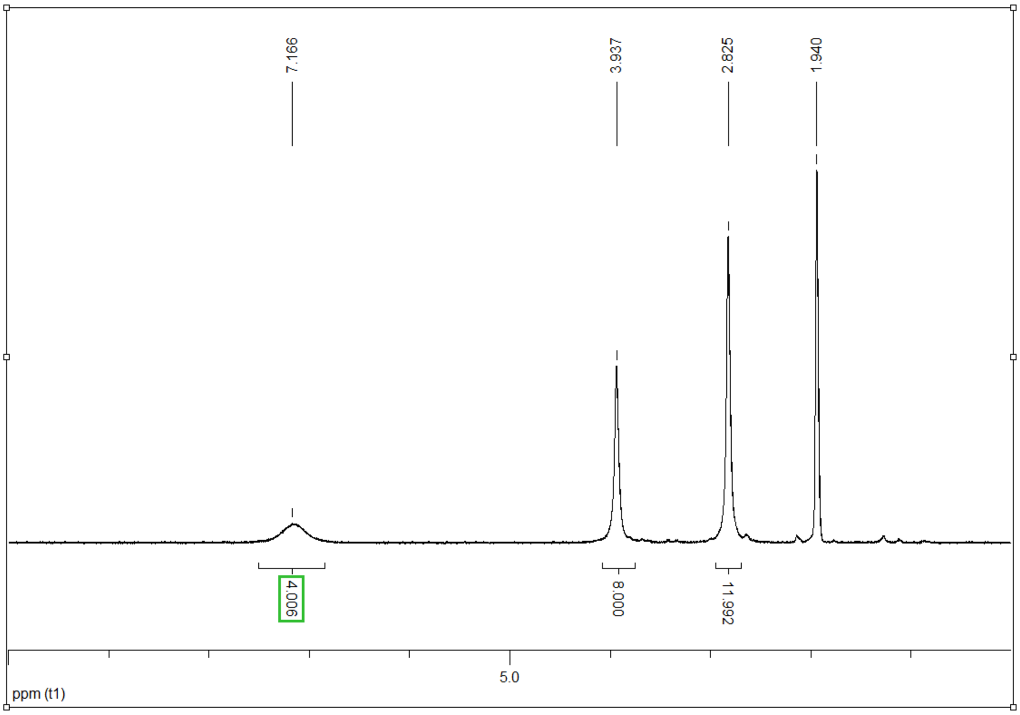

^{13}C NMR spectrum (100.56 MHz, CD_3CN, 295.1 K) of compound **3**$(PF_6)_2$

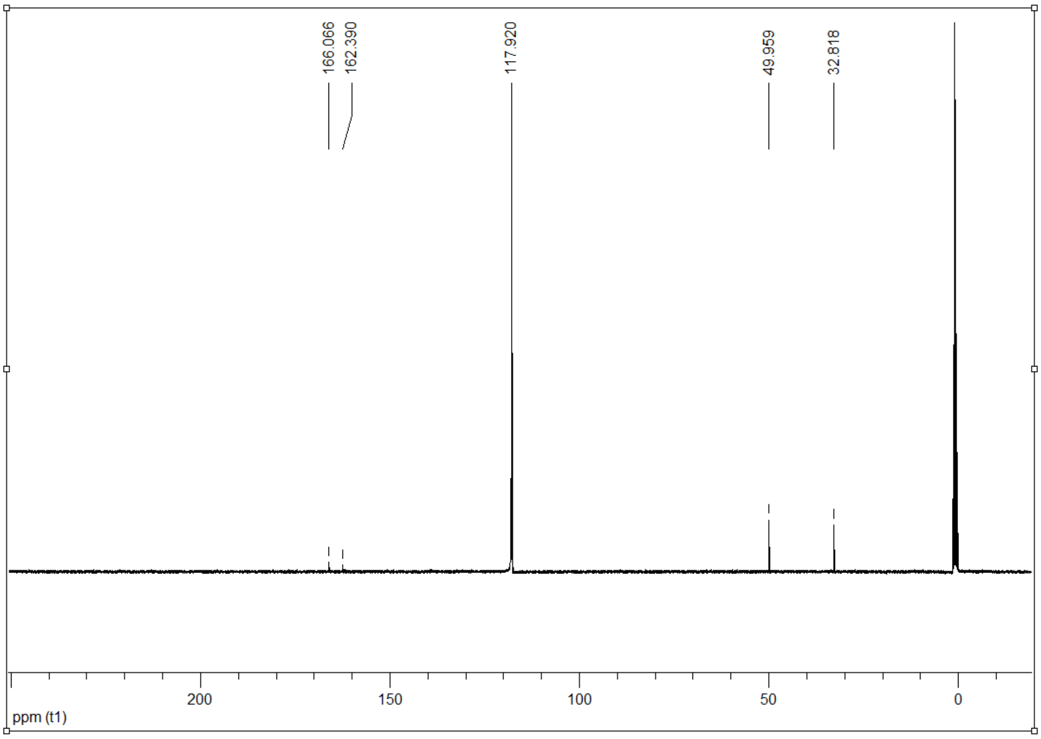

^{1}H NMR spectrum (399.89 MHz, CD_3CN, 295.3 K) of compound (**3**+2H)$(PF_6)_2$

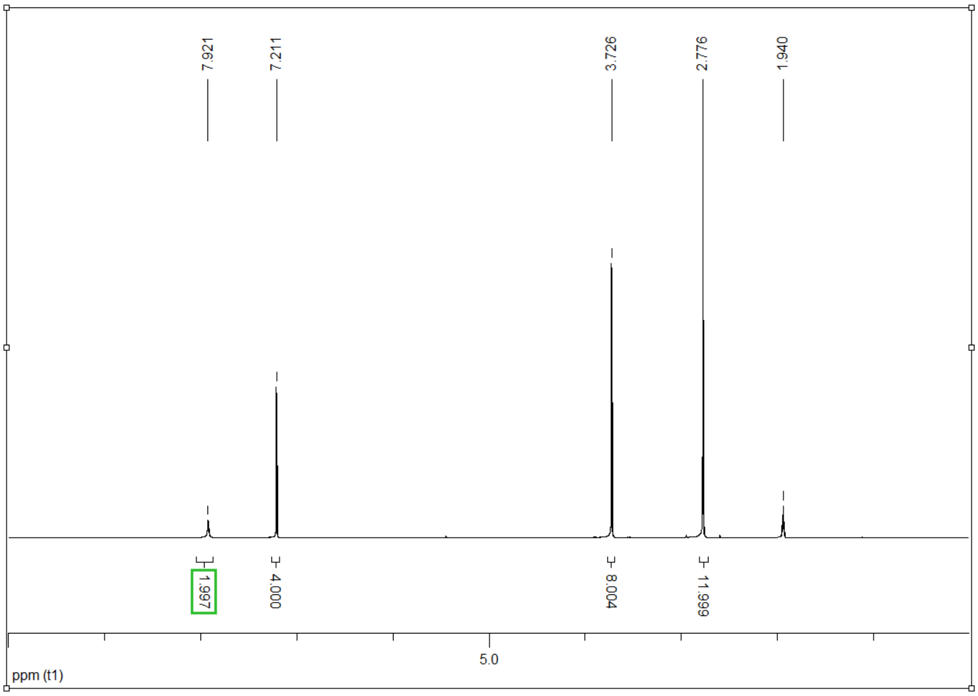

^{13}C NMR spectrum (100.56 MHz, CD_3CN, 295.9 K) for compound (**3**+2H)$(PF_6)_2$

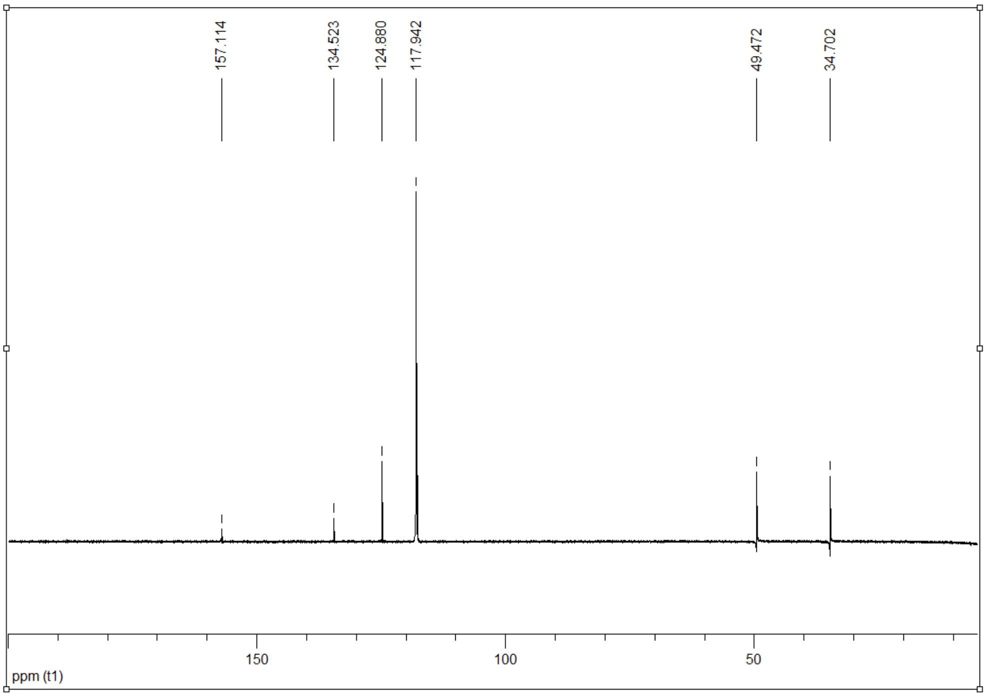

^{1}H NMR spectrum (399.89 MHz, CD_3CN, 298.2 K) for compound (**2**+H)(PF_6) (1:1 mixture of **2** + (**2**+2H)(PF_6)$_2$)

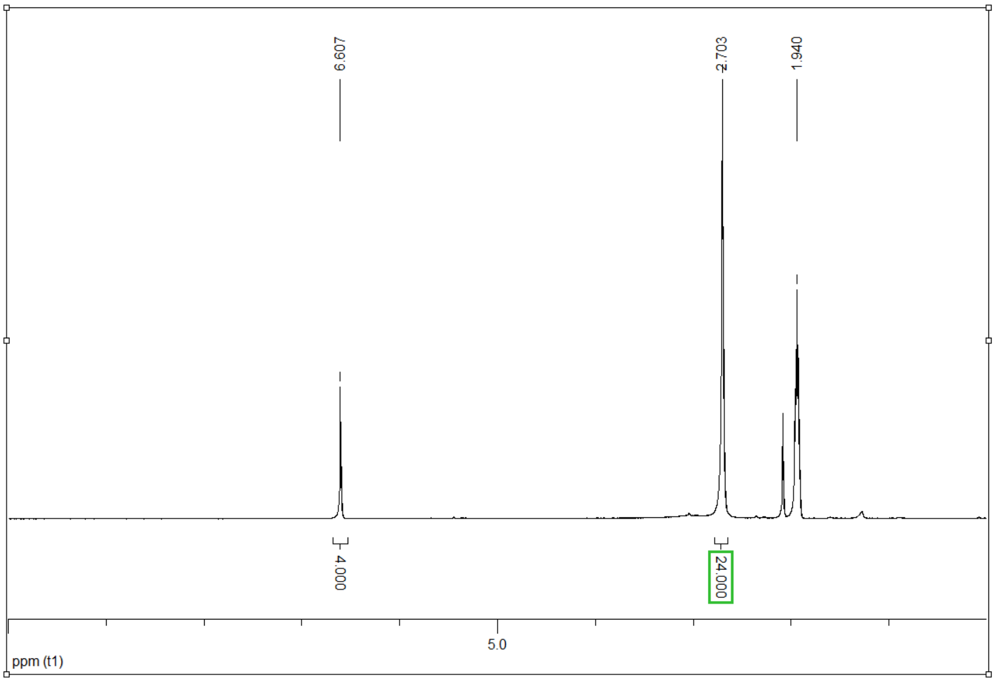

^{13}C NMR spectrum (100.56 MHz, CD_3CN, 295.9 K) for compound (**2**+H)(PF_6) (1:1 mixture of **2** + (**2**+2H)(PF_6)$_2$)

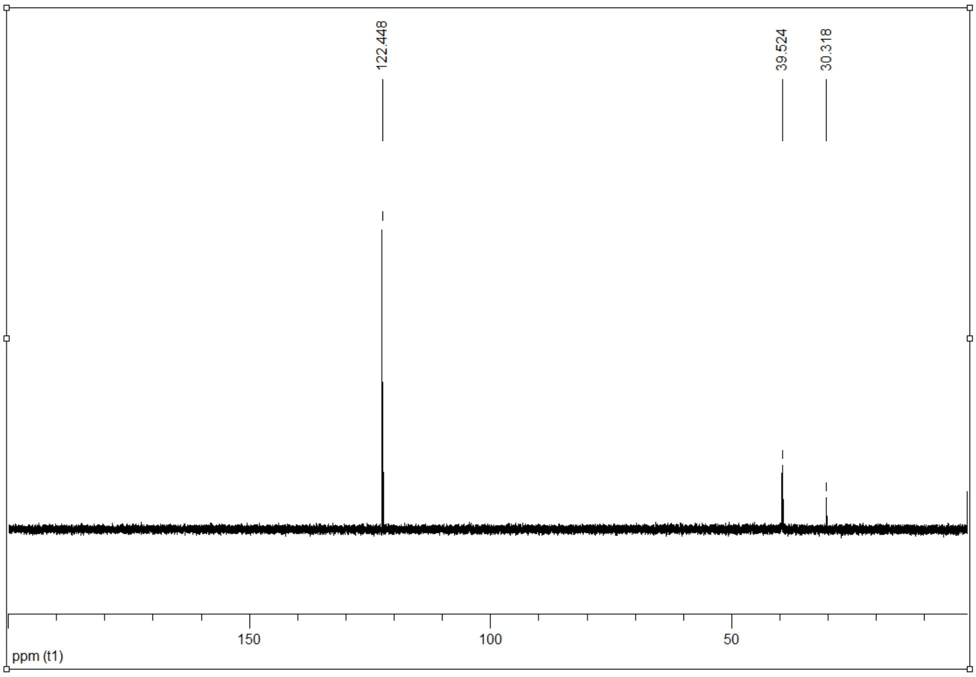

^{1}H NMR spectrum (399.89 MHz, CD_3CN, 298.1 K) for compound (**3**+H)(PF_6)

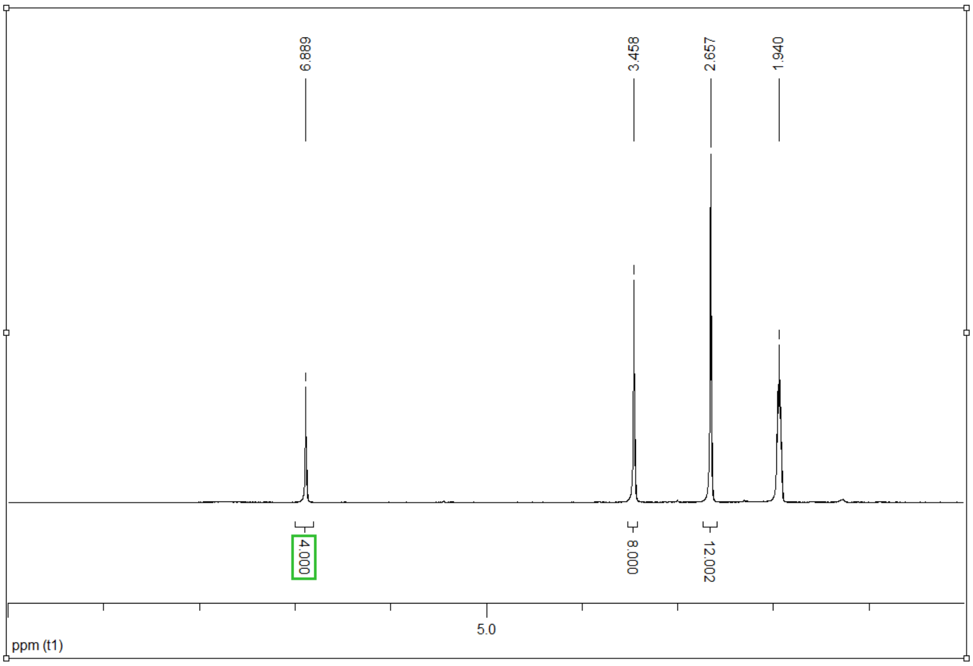

^{1}H NMR spectrum (399.89 MHz, CD_3CN, 294.8 K) for compound **2**(PF_6)$_2$ with 10 eq. HBF_4·OEt_2 after 30 min, 298 K

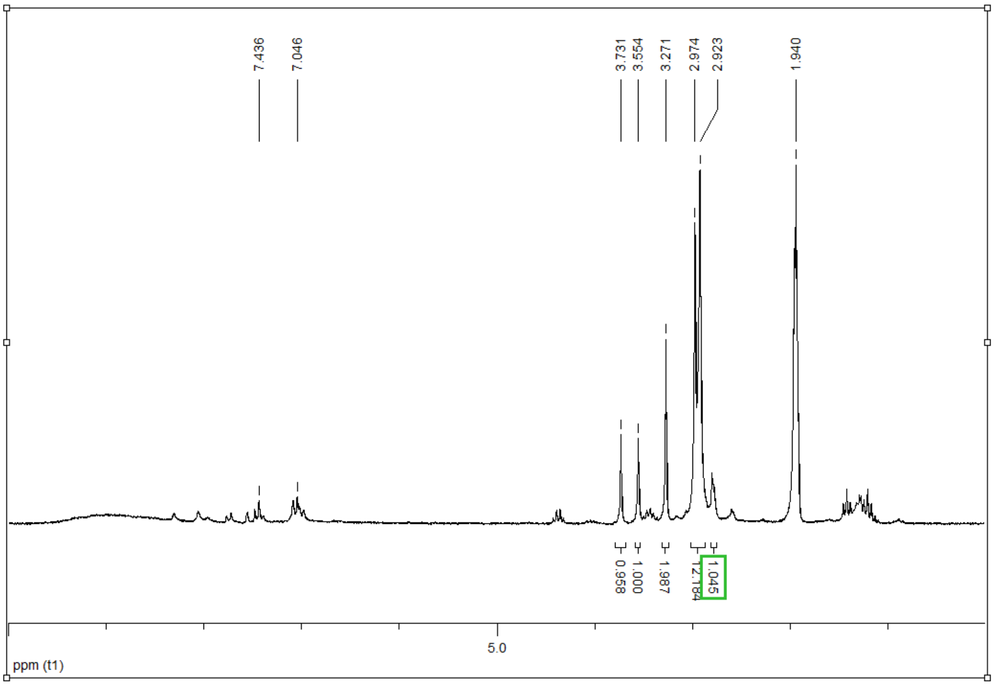

^{1}H NMR spectra (399.89 MHz, CD_3CN, 295.4 K) for compound **1**, (**1**+2H)$(PF_6)_2$, and 1:1 mixtures of **1** + (**1**+H)$(PF_6)_2$.

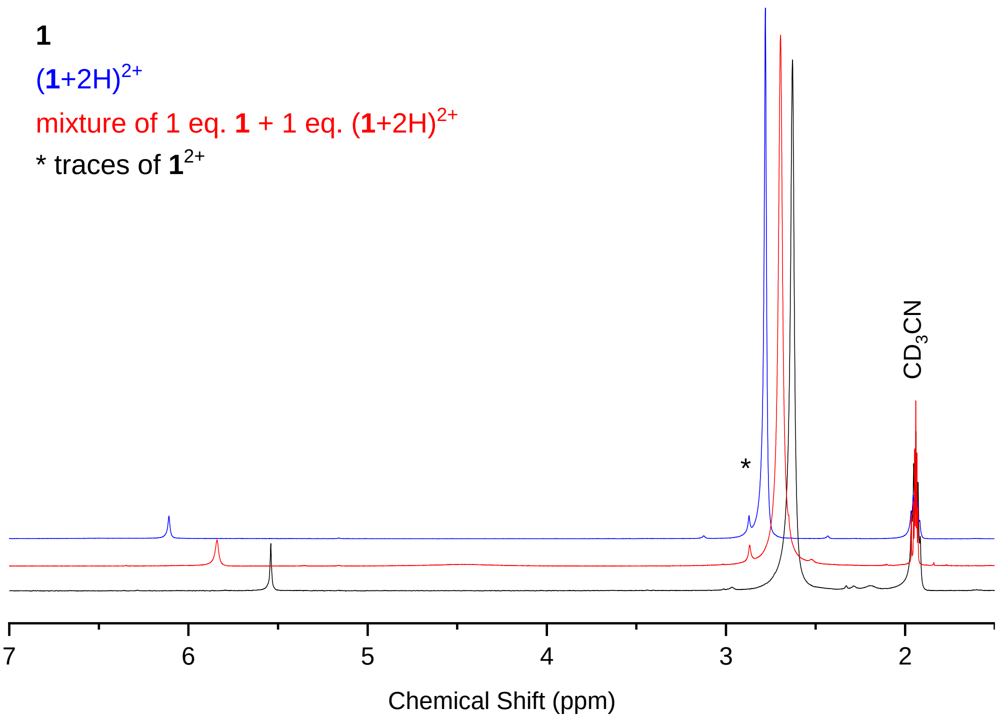

^{1}H NMR spectra (399.89 MHz, CD_3CN, 295.4 K) for compound **2**, (**2**+H)(PF_6) and (**2**+2H)$(PF_6)_2$

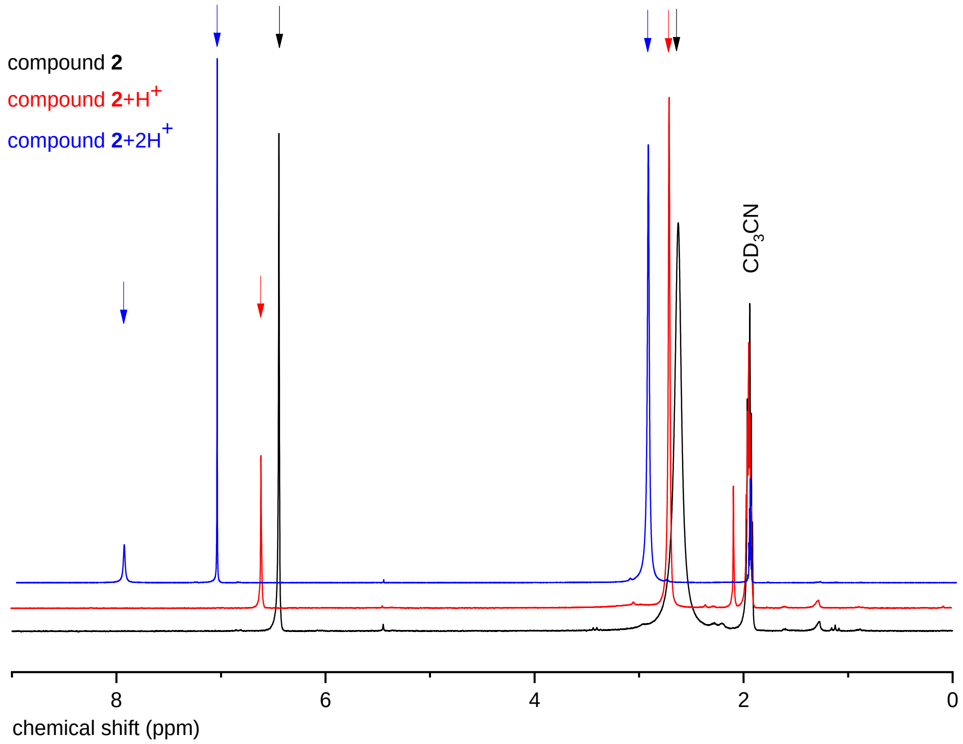

^1H NMR spectra (399.89 MHz, CD_3CN, 295.4 K) for compound **3**, (**3**+H)(PF_6) and (**3**+2H)$(PF_6)_2$

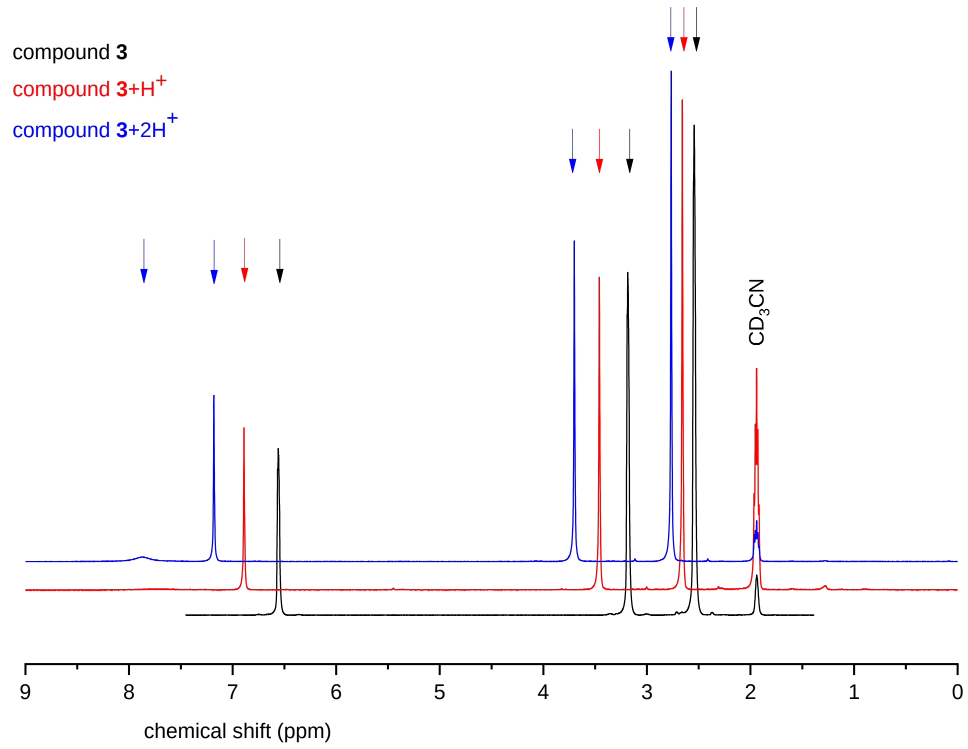

UV-vis spectra in CH_3CN solution

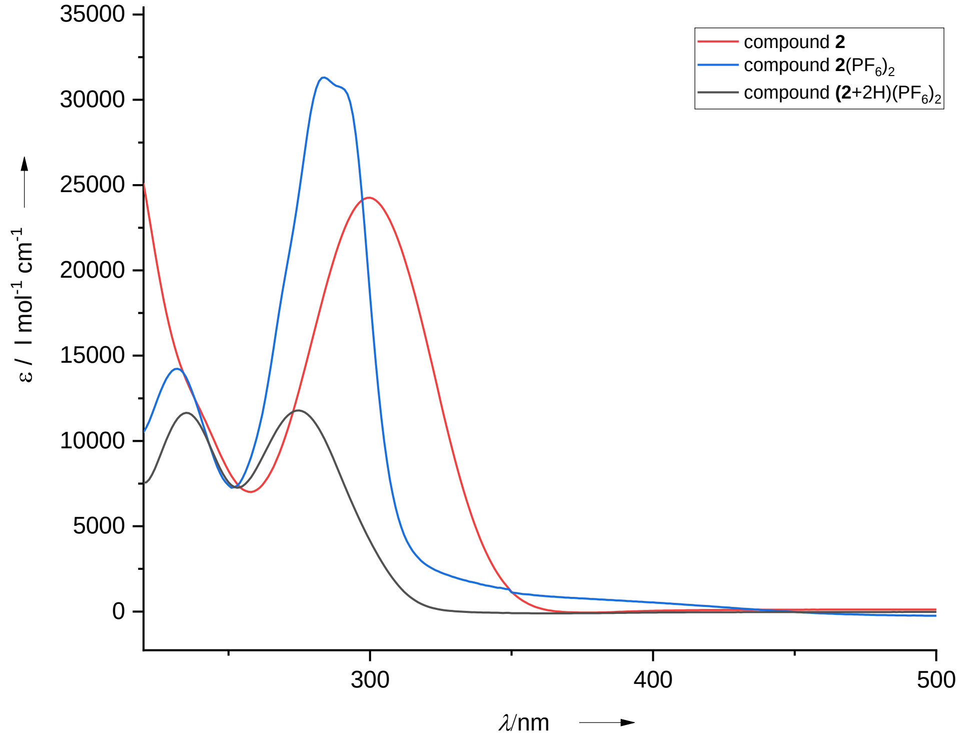

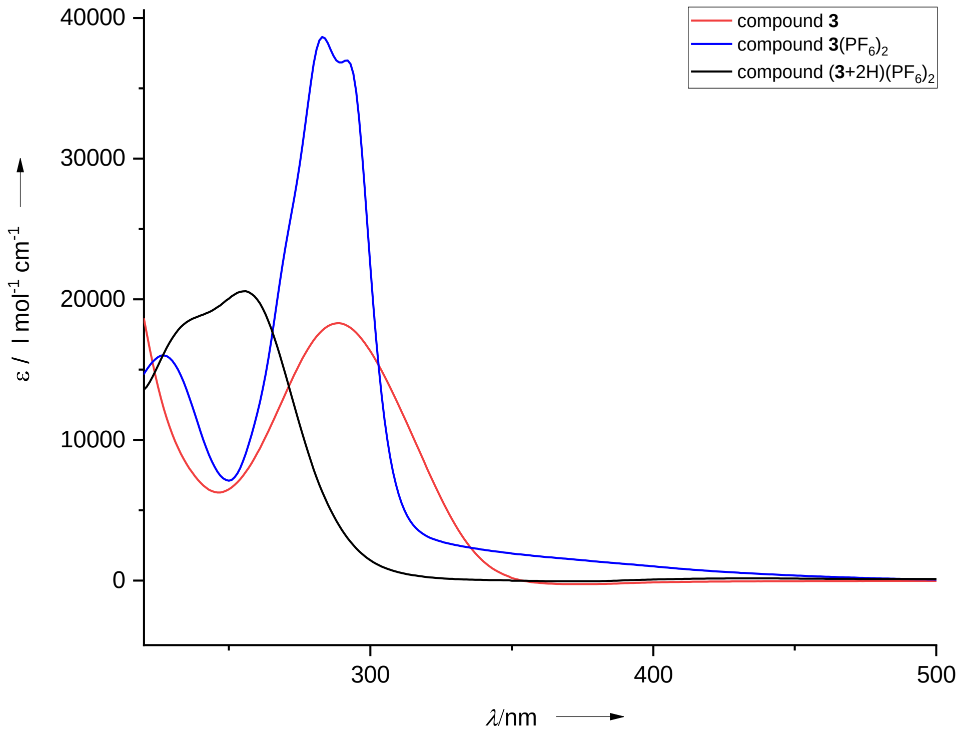

CV curves

CV curves (CH_3CN, Ag/AgCl reference electrode, 0.1 M N(^{n}Bu)$_4$(PF_6) as supporting electrolyte, scan speed 100 mV·s^{-1}) for the two compounds **2** and **3** measured in oxidation direction. Potentials given vs. Fc/Fc$^+$.

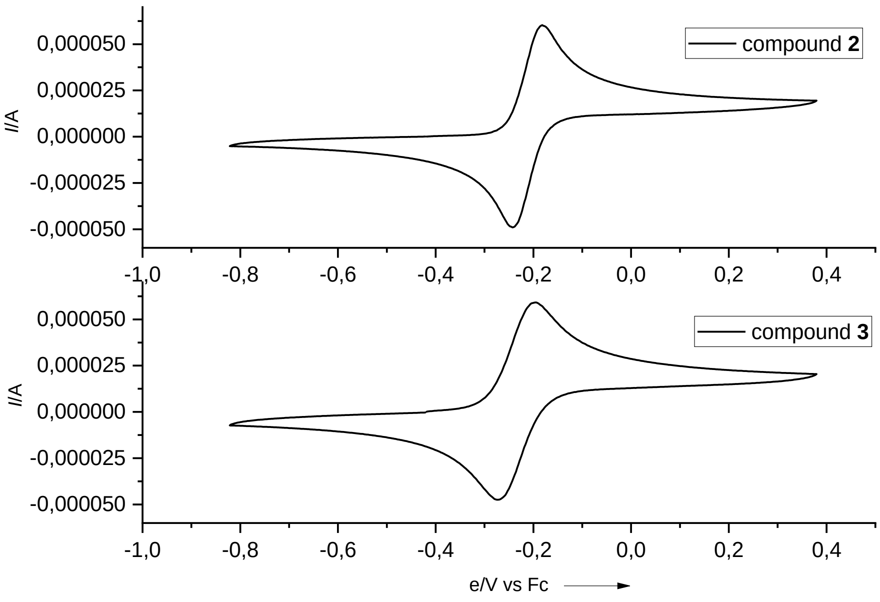

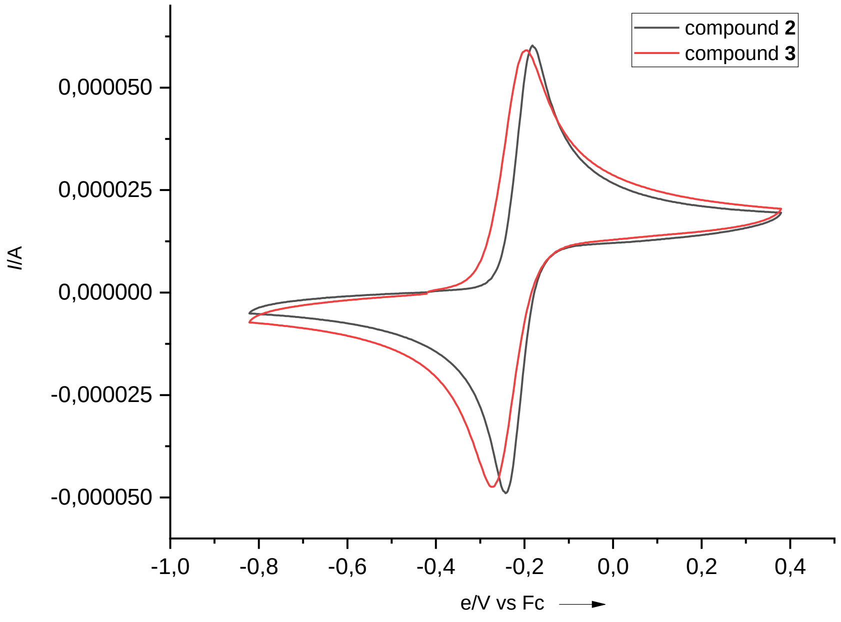

Details of the crystal structure determinations

	$(2+2H)(PF_6)_2$	3	$3(PF_6)_2$	$(3+H)(PF_6)$	$(3+2H)(PF_6)_2$
CCDC-No.	2013653	2013654	2013652	2013655	2013657
formula	$C_{16}H_{30}N_6P_2F_{12}$	$C_{16}H_{24}N_6$	$C_{16}H_{24}N_6P_2F_{12}$	$C_{16}H_{25}N_6PF_6$	$C_{16}H_{26}N_6F_{12}P_2$
crystal system	monoclinic	monoclinic	monoclinic	orthorhombic	monoclinic
space group	$P\ 2_1/c$	$P\ 2_1/c$	$P\ 2_1/c$	*P* b c a	$P\ 2_1/c$
a /Å	10.724(2)	8.0529(7)	6.0211(2)	9.9054(7)	7.5715(2)
b /Å	8.9420(18)	13.0270(11)	22.0229(8)	13.5468(9)	20.1691(5)
c /Å	12.887(3)	8.3789(7)	8.7572(3)	29.713(2)	8.3901
α /°	90	90	90	90	90
β /°	99.47(3)	112.935(3)	104.959(1)	90	116.291(1)
γ /°	90	90	90	90	90
V /Å^3	1219.0(4)	809.50(12)	1121.87(7)	3987.0(5)	1148.72(5)
Z	2	2	2	8	2
M_r	596.4	300.41	590.35	446.39	592.37
F_{000}	4768	324	600	1856	604.0
d_c /Mg·m^{-3}	1.6250	1.232	1.748	1.487	1.713
μ /mm^{-1}	0.288	0.078	0.313	0.208	0.306
max., min. transmission factors		0.7458, 0.7143	0.7461, 0.5334	0.7461, 0.7139	0.7465, 0.6730
X-radiation, λ /Å	Mo-$K\alpha$, 0.71073	Mo-$K\alpha$, 0.71073	Mo-$K\alpha$, 0.71073	Mo-$K\alpha$, 0.71073	Mo-$K\alpha$, 0.71073
data collect. temp. /K	120	100	100	100	100
θ range /°	2.983 to 28.490	2.746 to 27.097	2.579 to 28.998	2.47 to 30.17	2.89 to 33.15
index ranges h,k,l	-15 ... 15, -12 ... 11, -18 ... 18	-10 ... 10, -16 ... 16, -10 ... 10	-8 ... 7, 0 ... 30, 0 ... 11	-13 ... 13, -19 ... 18, -41 ... 41	-10 ... 11, -31 ... 30, -12 ... 12
reflections measured	5720	10624	61160	99187	37741
unique [R_{int}]	2567 [0.0283]	1780 [0.0474]	2975 [0.0595]	5790 [0.0780]	4360 [0.0394]
observed [$I \geq 2\sigma(I)$]	3025	1566	2658	4692	3917
data / restraints/ parameters	3025 / 0 / 167	1780 / 0 / 102	2975 / 0 / 166	5790 / 0 / 270	4360 / 0 / 169
GooF on F^2	1.045	1.042	1.036	1.060	1.049
R indices [$F>4\sigma(F)$] $R(F)$, $wR(F^2)$	0.0352, 0.0937	0.0396, 0.0982	0.0283, 0.0707	0.0379, 0.1375	0.0289, 0.1375
R indices (all data) $R(F)$, $wR(F^2)$	0.0426, 0.0986	0.0453, 0.1037	0.0333, 0.0742	0.0995, 0.0886	0.0780, 0.0757
largest residual peaks /e·Å^{-3}	0.291, -0.352	0.292, -0.175	0.363, -0.313	0.424, -0.339	0.471, -0.374

4 Experimental details for oxidative coupling of *N*-ethylcarbazole to *N,N'*-diethyl-3,3'-bicarbazole

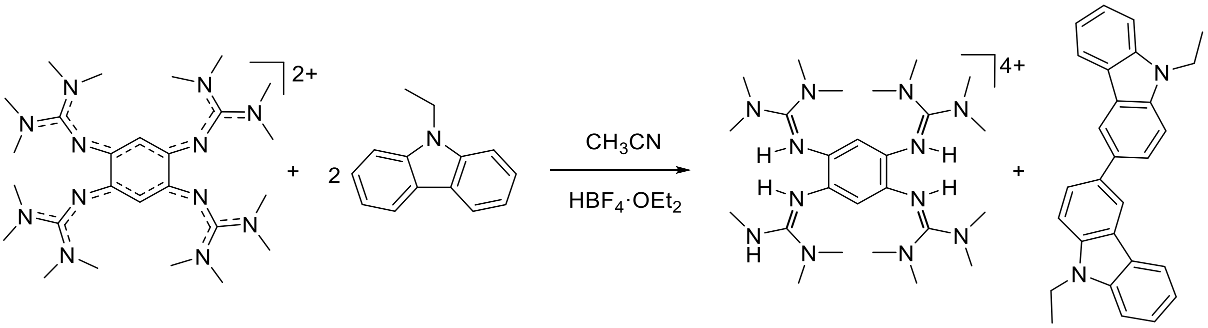

entry	A	B
**1**(ClO_4)$_2$ [mg/µmol]	16.754/22.96	-
**1**(BF_4)$_2$ [mg/µmol]	-	8.618/12.24
N-ethylcarbazole [mg/µmol]	4.526/23.18	2.382/12.20
$HBF_4{\cdot}OEt_2$ (5% in CH_3CN) [ml/µmol]	1/365	0.55/200
HMB [mg/µmol]	0.862/5.31	-
reaction time (temperature)	15 min (ice cooling)<br>45 min (r.t.)	15 min (ice cooling)<br>35 min (r.t.)
conversion to *N,N'*-diethyl-3,3'-bicarbazole [%]	95	94
ratio (GFA/substrate)	1/1	1/1

General protocol

In a Schlenk flask, a $HBF_4{\cdot}OEt_2$ solution (5% in CH_3CN; c = 0.364 mol·l^{-1}) was added under ice cooling in an argon atmosphere to the reactants $\mathbf{1}^{2+}$ and *N*-ethylcarbazole. The reaction was stirred for 15 min under ice cooling and then allowed to warm up to room temperature.

The reaction was stopped by the addition of an aqueous saturated $NaHCO_3$ solution. The small amount of CH_3CN and $HBF_4{\cdot}OEt_2$ was removed under high-vacuum. Then an aqueous diluted solution of NaOH (8%) was added (pH > 9), converting all formed ($\mathbf{1}$+4H)$^{4+}$ to ($\mathbf{1}$+2H)$^{2+}$ (for subsequent transfer into the organic phase). A clear solution was obtained that was extracted several times with CH_2Cl_2. The combined organic phases were dried over Na_2SO_4, filtrated and condensed.

^{1}H NMR spectrum (600.13 MHz, 295 K, CD_2Cl_2) for entry A

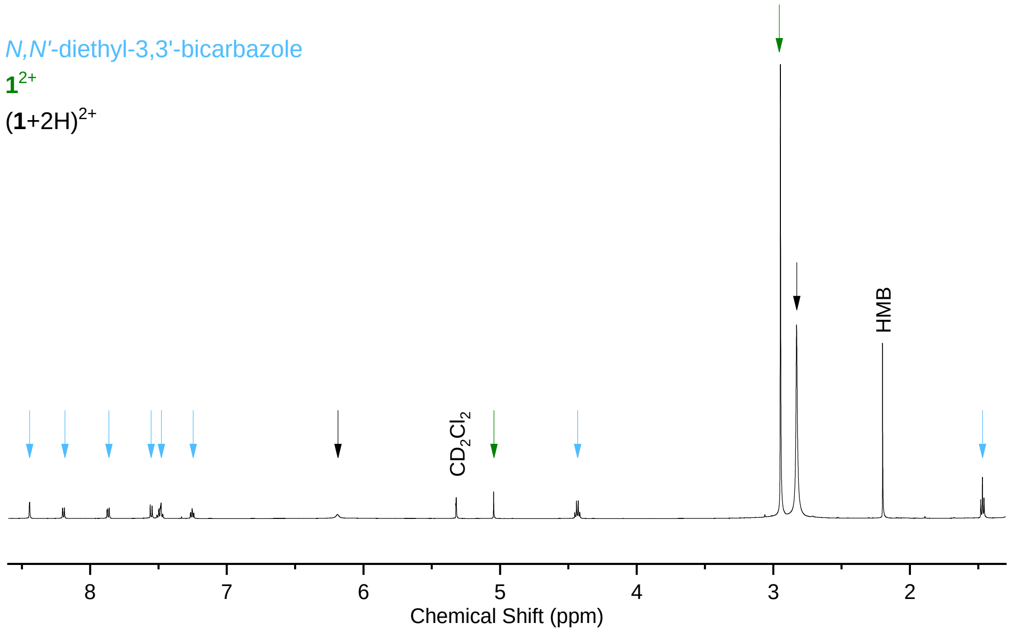

^{1}H NMR of *N,N'*-diethyl-3,3'-bicarbazole: (600.13 MHz, 295 K, CD_2Cl_2): δ = 1.47 (t, J = 7.2, 7.2 Hz, 6 H), 4.43 (q, J = 7.3, 7.2, 7.2 Hz, 4 H), 7.25 (ddd, J = 7.8, 6.5, 1.5 Hz, 2 H), 7.51 - 7.47 (m, 4 H), 7.55 (d, J = 8.4, 2 H), 7.87 (dd, J = 8.4, 1.8 Hz, 2 H), 8.20 (dd, J = 7.7, 0.7 Hz, 2 H), 8.44 (bs, 2 H) ppm.

5 Experimental details for oxidative coupling of 3,3''-dimethoxy-3',4'-dimethyl-*o*-terphenyl to 3,10-dimethoxy-6,7-dimethyltriphenylene

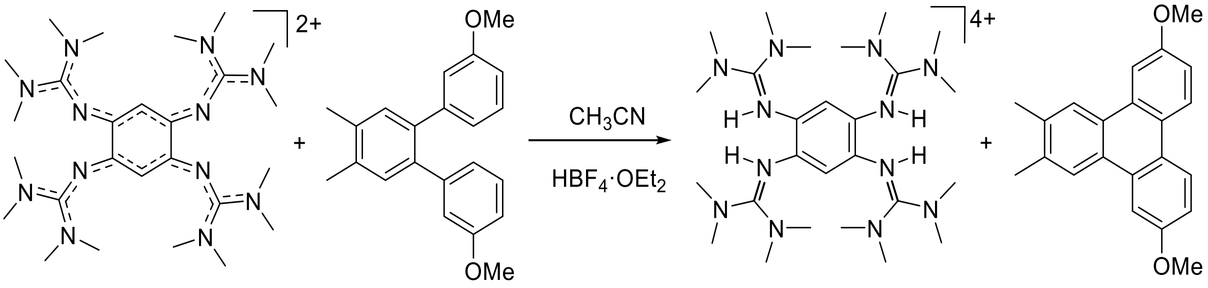

entry	A	B
**1**$(BF_4)_2$ [mg/µmol]	6.562/9.32	-
**1**$(PF_6)_2$ [mg/µmol]	-	7.402/9.02
3,3''-dimethoxy-3',4'-dimethyl-*o*-terphenyl [µmol]	6.25	5.7
$HBF_4{\cdot}OEt_2$ (5% in CH_3CN) [ml/µmol]	0.5/182	0.5/182
HMB [mg/µmol]	1.618/9.97	0.734/4.52
reaction time (temperature)	10 min (ice cooling)<br>120 min (r.t.)	10 min (ice cooling)<br>85 min (r.t.)
conversion to 3,10-dimethoxy-6,7-dimethyltriphenylen [rel. to applied substrate, %]	76	71
ratio (GFA/substrate)	1/0.7	1/0.6

General protocol

In a Schlenk flask, 0.5 ml of a $HBF_4{\cdot}OEt_2$ solution (5% in CH_3CN, c = 0.364 mol·l^{-1}) was added under ice cooling in an argon atmosphere to the reactants $\mathbf{1}^{2+}$ and to an appropriate volume of a 3,3''-dimethoxy-3',4'-dimethyl-*o*-terphenyl - solution (c = 0.284 mol·l^{-1} in CH_3CN). The reaction was stirred for 10 min under ice cooling and then allowed to warm up to room temperature.

The reaction was stopped by the addition of an aqueous saturated $NaHCO_3$ solution. The small amount of CH_3CN and $HBF_4{\cdot}OEt_2$ was removed under high-vacuum. Then an aqueous diluted solution of NaOH (8%) was added (pH > 9), converting all formed $(\mathbf{1}+4H)^{4+}$ to $(\mathbf{1}+2H)^{2+}$ (for subsequent transfer into the organic phase). A clear solution was obtained that was extracted several times with CH_2Cl_2. The combined organic phases were dried over Na_2SO_4, filtrated and condensed.

^{1}H NMR spectrum (399.87 MHz, 294.5 K, CD_2Cl_2) for entry A

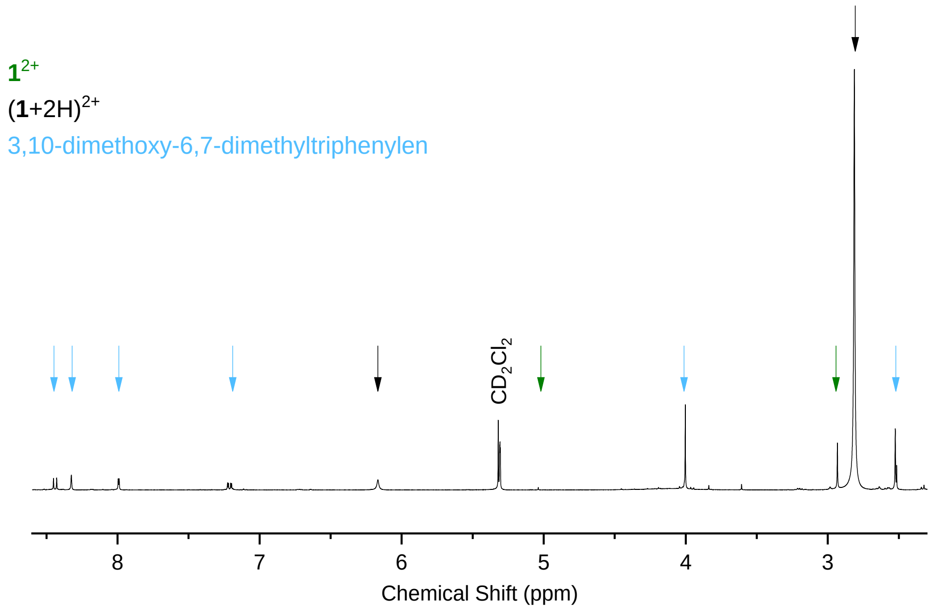

^{1}H NMR of 3,10-dimethoxy-6,7-dimethyltriphenylen: (399.87 MHz, 294.5 K, CD_2Cl_2):
δ = 2.53 (s, 6 H), 4.00 (s, 6 H), 7.21 (dd, J = 9.0, 2.6 Hz, 2 H), 7.99 (d, 2.6 Hz, 2 H), 8.33 (s, 2 H), 8.44 (d, J = 9.0 Hz, 2 H) ppm.

6 Experimental details for reaction of 1(PF_6)$_2$ with 10-methyl-9,10-dihydroacridine (AcrH$_2$)

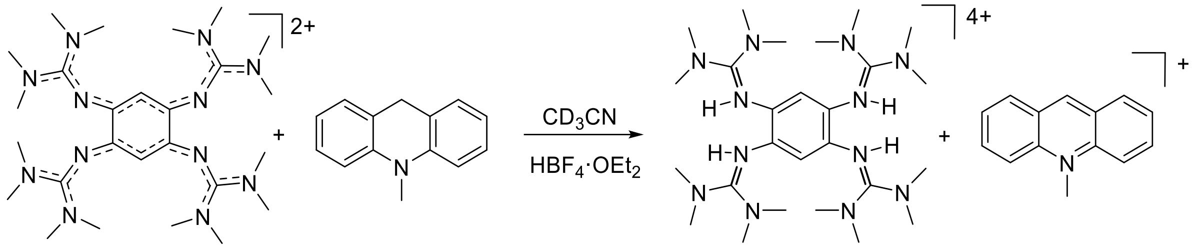

entry	A	B	C
**1**(PF_6)$_2$ [mg/µmol]	5.308/6.47	5.354/6.52	5.312/6.47
AcrH$_2$ [µmol]	6.45	6.45	6.45
HMB [mg/µmol]	0.794/4.89	1.05/6.47	0.932/5.74
$HBF_4 \cdot OEt_2$ (10% in CD_3CN) [µl/µmol/eq. rel. to **1**(PF_6)$_2$]	60/44/7	90/65/10	-
CD_3CN [ml]	0.45	0.45	0.45
reaction time (temperature)	390 min (r.t.)	250 min (r.t.)	2 d (60°C)
ratio (GFA/substrate)	1/1	1/1	1/1
conversion to AcrH$^+$ [%]	90	92	traces

General protocol (NMR experiments)

Under an argon atmosphere 0.25 ml of a solution of 10-methyl-9,10-dihydroacridine (c = 0.0258 mol·l^{-1} in CH_3CN) was transferred in a flame-sealable NMR tube and the solvent was removed under high-vacuum. In a Schlenk flask **1**(PF_6)$_2$ and HMB were dissolved in 0.45 ml CD_3CN and added via syringe to the 10-methyl-9,10-dihydroacridine. $HBF_4 \cdot OEt_2$ (10% in CD_3CN, c = 0.729 mol·l^{-1}) was added via microliter syringe (entry **A**, **B**). The NMR tube was flame-sealed in vacuum at -196 °C.

Experimental Details for 10-methyl-9,10-methylacridane ($AcrH_2$)

This compound was synthesised according to literature procedure[1] using 750 mg (19.7 mmol) $LiAlH_4$, 375 mg (1.71 mmol) 10-methylacridone and 50 ml diethylether.

Workup[2]: the reaction mixture was quenched by H_2O, extracted several times with ethylacetate and dried over $MgSO_4$. After the organic layer had been filtered and concentrated the crude product was purified by column chromatography on silica gel (d = 2.5 cm, h = 20 cm, PE (40/60)/toluene = 4/1, R_f: 0.39) to afford 270 mg (1.38 mmol, 81%) of a white solid. Elemental analysis calcd. (%) for $C_{14}H_{13}N$ (195.26 g·mol^{-1}): C 86.12, H 6.71, N 7.17; found C 86.04, H 6.94, N 7.24.

^{1}H NMR spectrum (399.89 MHz, 295 K, CD_3CN) for 10-methyl-9,10-dihydroacridine ($AcrH_2$)

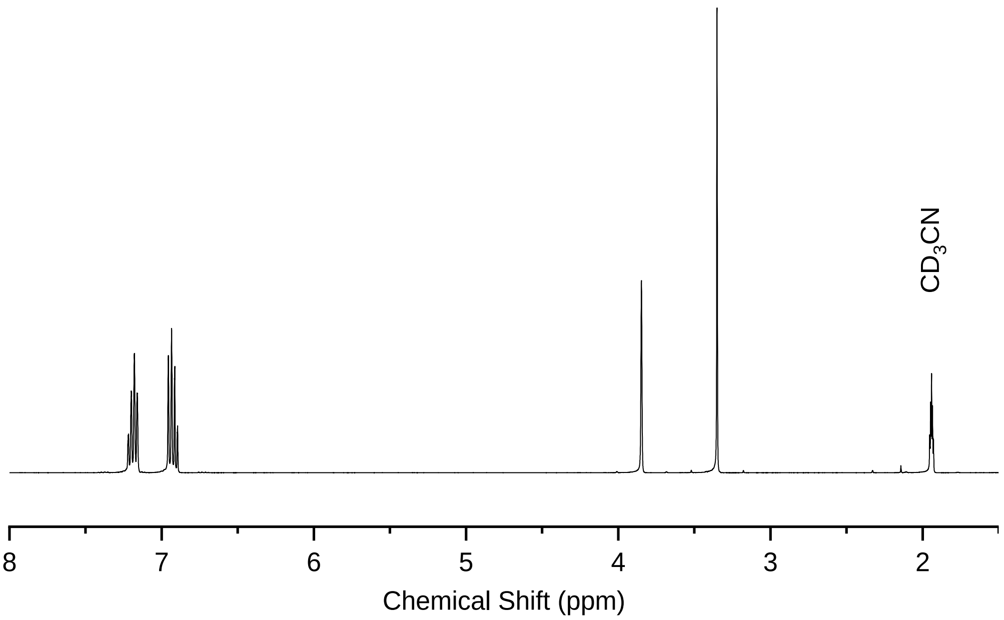

^{1}H NMR of $AcrH_2$: (399.89 MHz, 295 K, CD_3CN): δ = 3.35 (s, 3 H), 3.85 (s, 2 H), 6.94 (m, 4 H), 7.18 (m, 4 H) ppm.

[1] P. Karrer, L. Szabo, H. J. Krishna, R. Schwyzer, *Helv. Chim. Acta* **1950**, *33*, 294-300.
[2] Á. Pintér, A. Sud, D. Sureshkumar, M. Klussmann, *Angew. Chem. Int. Ed.* **2010**, *49*, 5004-5007.

^{13}C NMR spectrum (100.56 MHz, 295 K, CD_3CN) for 10-methyl-9,10-dihydroacridine ($AcrH_2$)

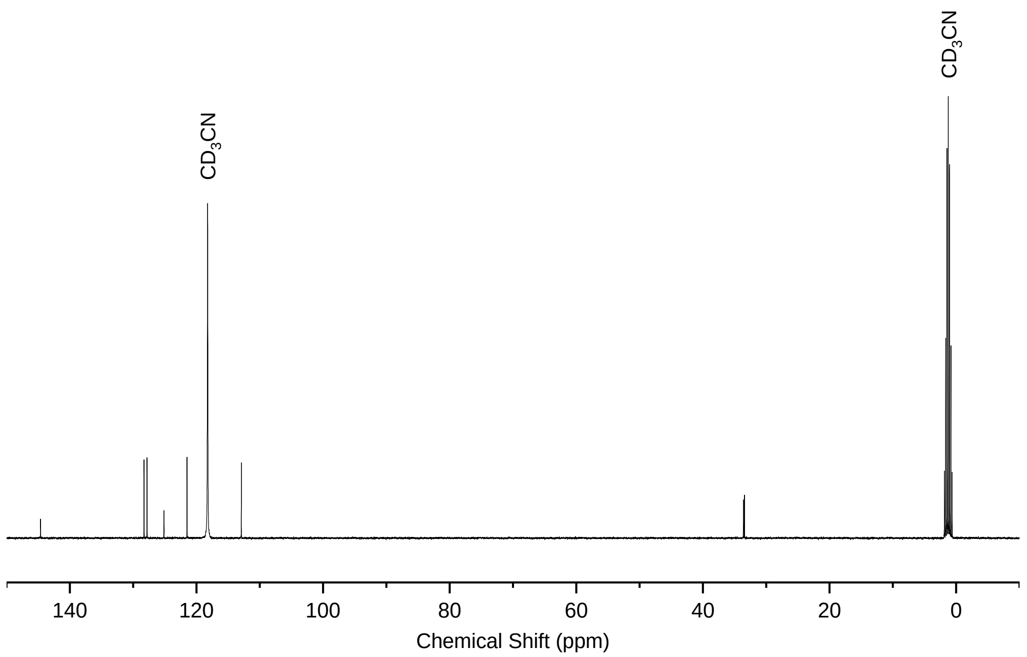

^{13}C NMR of $AcrH_2$: (100.56 MHz, 295 K, CD_3CN): δ = 33.44, 33.60, 112.92, 121.48, 125.15, 127.80, 128.27, 144.62 ppm.

^{1}H NMR spectrum (199.87 MHz, 298 K, CD_3CN) for entry A: first measurement

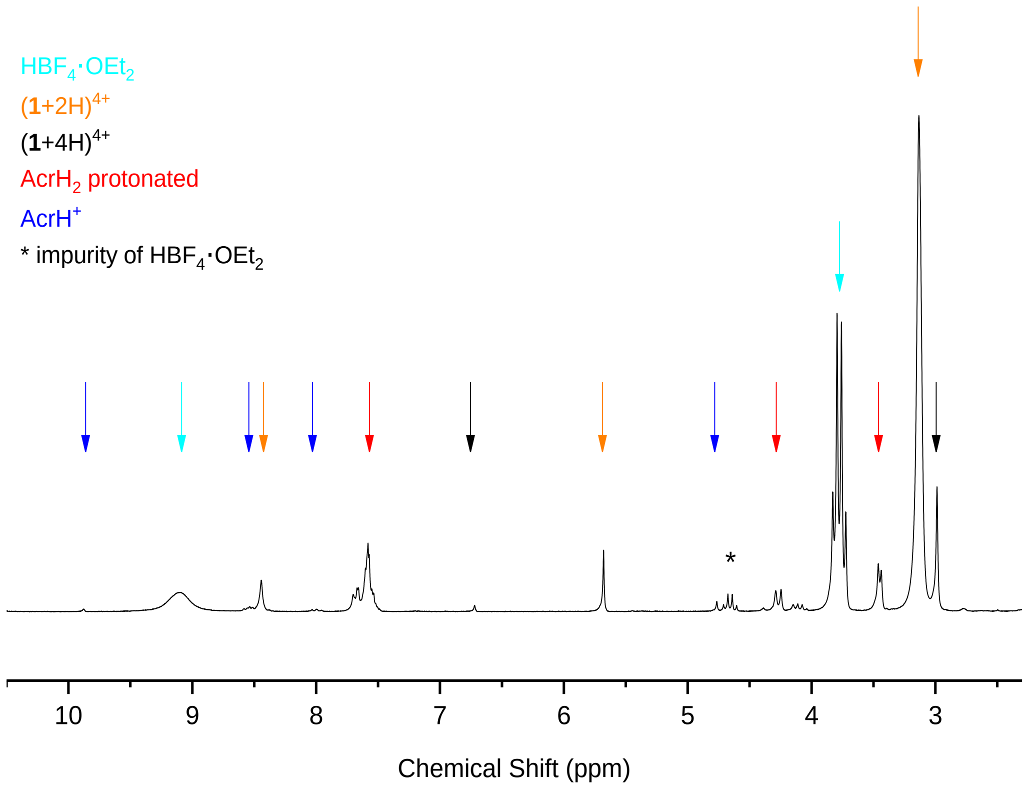

^{1}H NMR spectrum (600.13 MHz, 295 K, CD_3CN) for entry A: last measurement

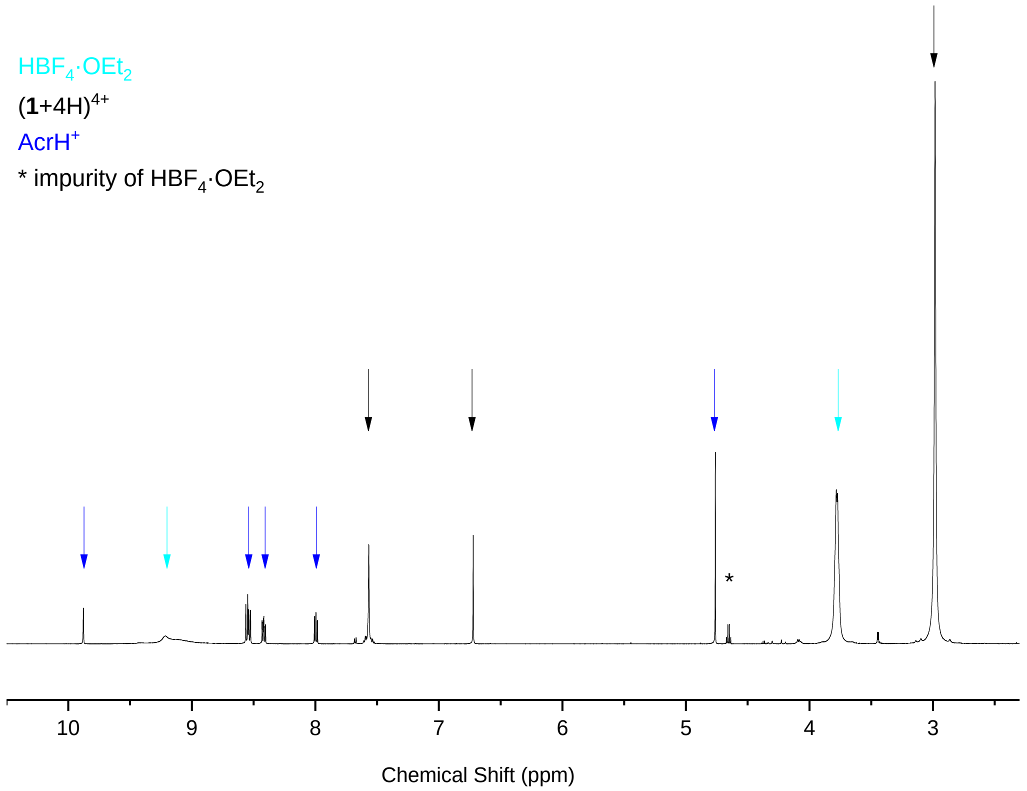

7 Experimental details for reaction of 1(PF_6)$_2$ with 10-methyl-[9,9'-$^2H_2$]-acridine (AcrD$_2$)

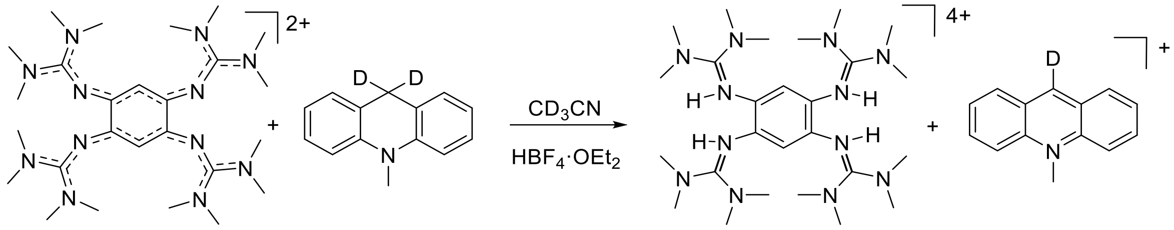

entry	A	B
**1**(PF_6)$_2$ [mg/µmol]	5.364/6.54	5.382/6.56
AcrD$_2$ [µmol]	6.50	6.50
HMB [mg/µmol]	0.708/4.36	0.622/3.83
$HBF_4 \cdot OEt_2$ (10% in CD_3CN) [µl/µmol/ eq. rel. to **1**(PF_6)$_2$]	40/29/4.5	90/65/10
CD_3CN [ml]	0.45	0.45
reaction time (temperature)	630 min (r.t.)	400 min (r.t.)
ratio (GFA/substrate)	1/1	1/1
conversion to AcrD$^+$ [%]	87	90

General protocol (NMR experiments)

Under an argon atmosphere 0.25 ml of a solution of 10-methyl-[9,9'-$^2H_2$]-10-acridine ($c = 0.0260$ mol·l^{-1} in CH_3CN) was transferred into a flame-sealable NMR tube and the solvent was removed under high-vacuum. In a Schlenk flask **1**(PF_6)$_2$ and HMB were dissolved in 0.45 ml CD_3CN and added via syringe to the [9,9'-$^2H_2$]-10-methylacridine. $HBF_4 \cdot OEt_2$ (10% in CD_3CN, $c = 0.729$ mol·l^{-1}) was added via microliter syringe. The NMR tube was flame-sealed in vacuum at -196 °C.

Experimental Details for 10-methyl-[9,9'-$^2H_2$]-acridine ($AcrD_2$)

This compound was synthesised according to literature procedure[1] using 255.7 mg (6.1 mmol) $LiAlD_4$, 116 mg (0.554 mmol) 10-methylacridone and 30 ml diethylether.

Workup[2]: the reaction mixture was quenched by H_2O, extracted several times with ethylacetate and dried over $MgSO_4$. After the organic layer had been filtered and concentrated the crude product was purified by column chromatography on silica gel (d = 2.5 cm, h = 20 cm, PE (40/60)/toluene 4:1, R_f: 0.33) to afford 62 mg (0.31 mmol, 57%) of a white solid. Elemental analysis calcd. (%) for $C_{14}H_{11}ND_2$ (197.27 g·mol^{-1}): C 85.24, H 6.64, N 7.10; found C 85.06, H 6.98, N 6.85.

^{1}H NMR spectrum (399.89 MHz, 295.2 K, CD_3CN) for 10-methyl-[9,9'-$^2H_2$]-acridine ($AcrD_2$)

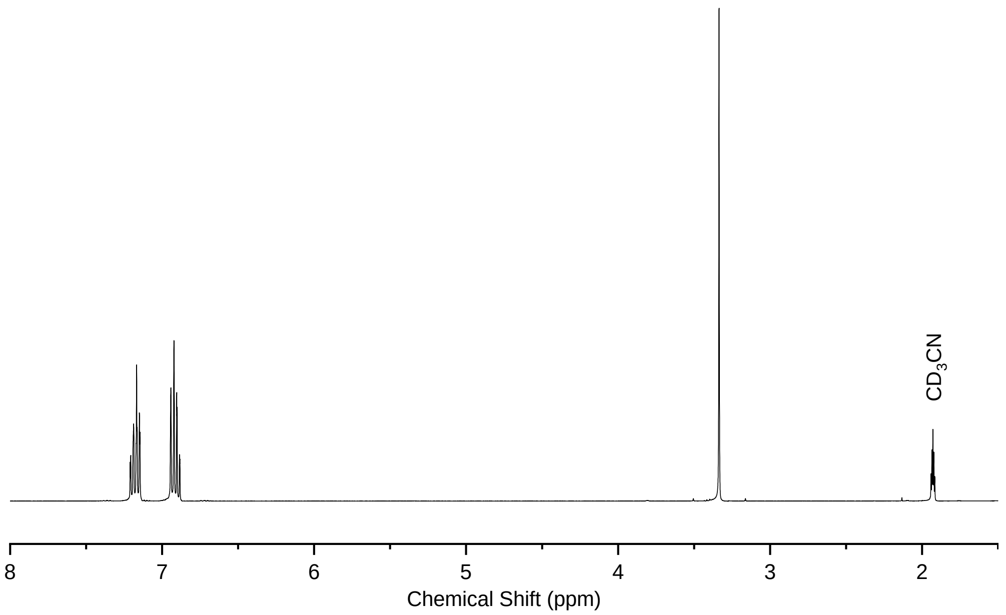

^{1}H NMR of $AcrD_2$: (399.87 MHz, 295.2 K, CD_3CN): δ = 3.35 (s, 3 H), 6.94 (m, 4 H), 7.18 (m, 4 H) ppm.

[1] P. Karrer, L. Szabo, H. J. Krishna, R. Schwyzer, *Helv. Chim. Acta* **1950**, *33*, 294-300.
[2] Á. Pintér, A. Sud, D. Sureshkumar, M. Klussmann, *Angew. Chem. Int. Ed.* **2010**, *49*, 50–04-5007.

^{13}C NMR spectrum (100.56 MHz, 295.2 K, CD_3CN) for 10-methyl-[9,9’-$^2H_2$]-acridine ($AcrD_2$)

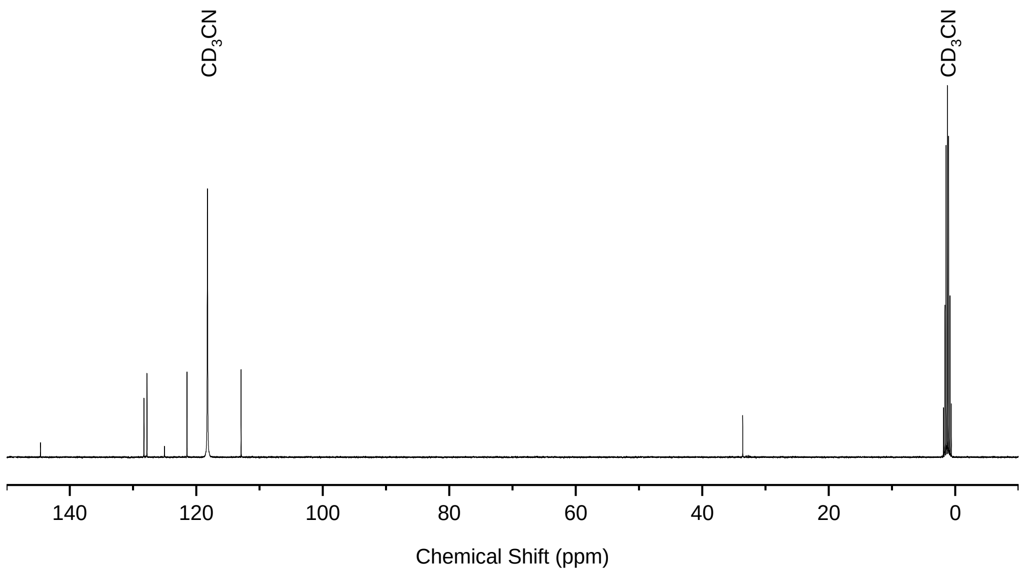

^{13}C NMR of $AcrD_2$: (100.56 MHz, 295.3 K, CD_3CN): δ = 33.60, 112.91,121.47, 125.04, 127.80, 128.28, 144.62 ppm.

^2D NMR spectrum (92.12 MHz, 295 K, CH_3CN) for 10-methyl-[9,9’-$^2H_2$]-acridine ($AcrD_2$)

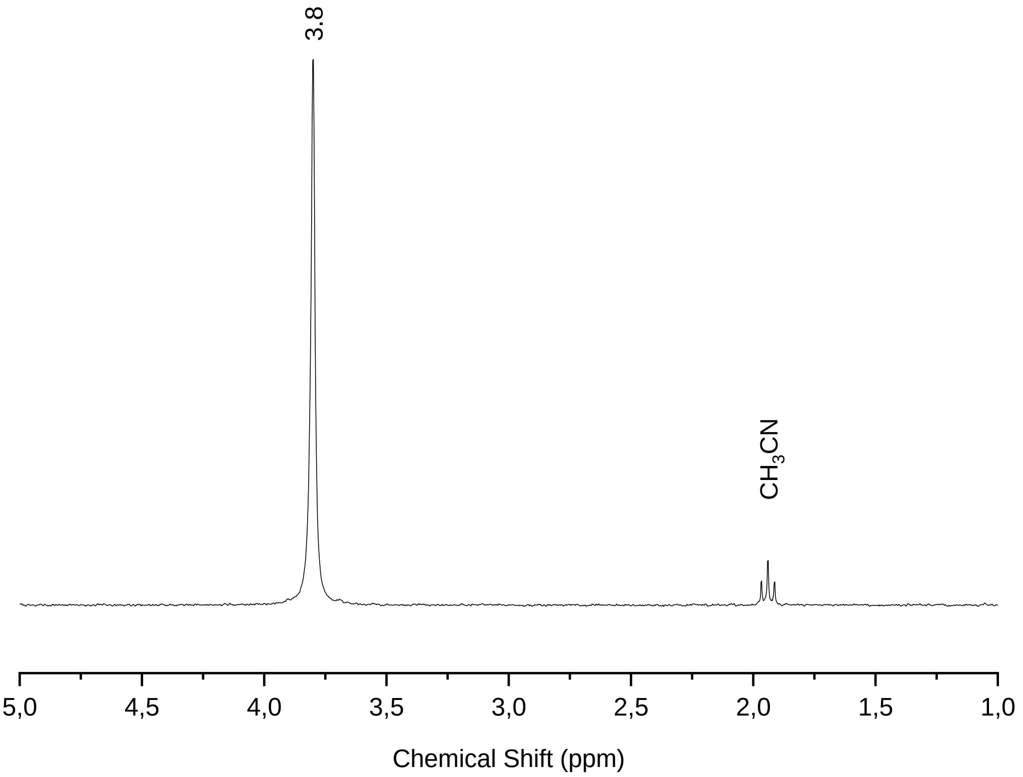

^{1}H NMR spectrum (199.87 MHz, 298 K, CD_3CN) for entry A: first measurement

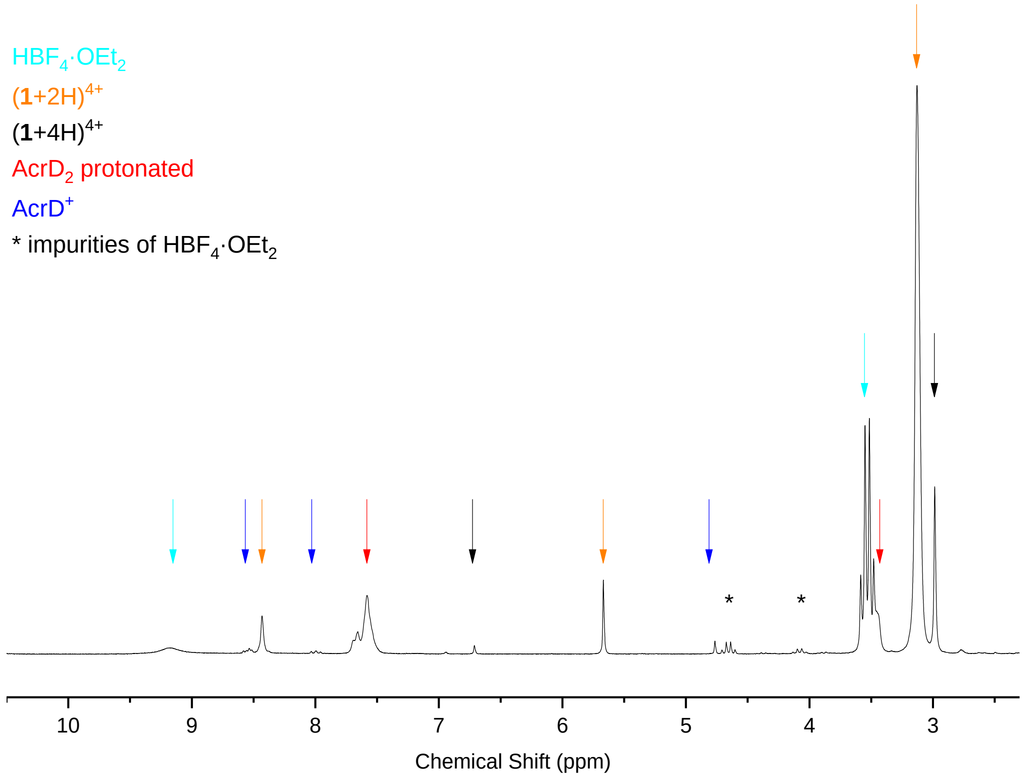

^{1}H NMR spectrum (399.89 MHz, 295.2 K, CD_3CN) for entry A: last measurement

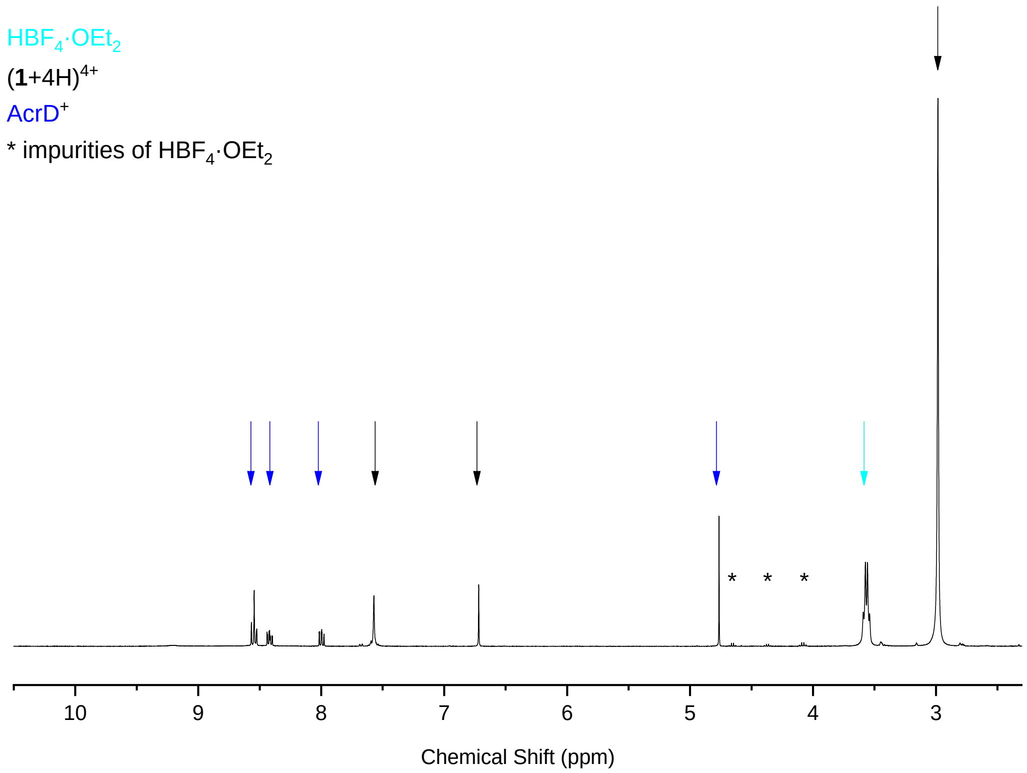

Dependence of the conversion vs. time plots for the reaction between **1**$(PF_6)_2$ and $AcrD_2$ on the applied equivalents of $HBF_4{\cdot}OEt_2$

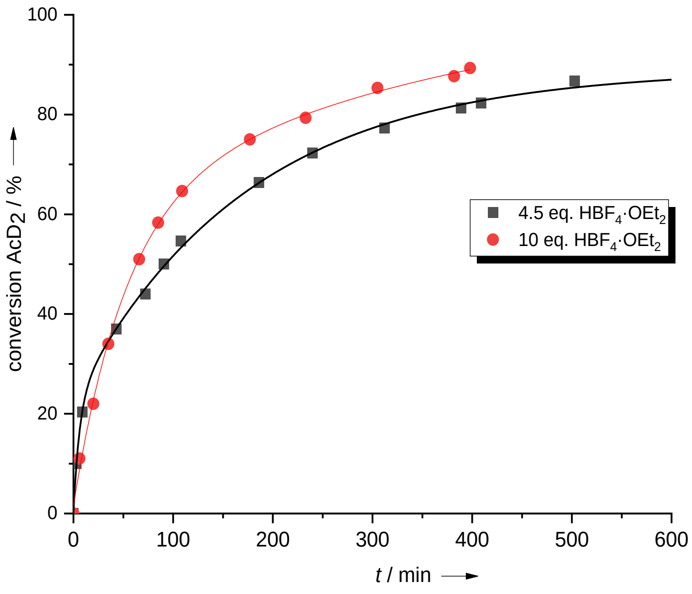

Comparison between the conversion vs. time plots for $AcrH_2$ and $AcrD_2$ in the presence of 10 eq. of $HBF_4{\cdot}OEt_2$

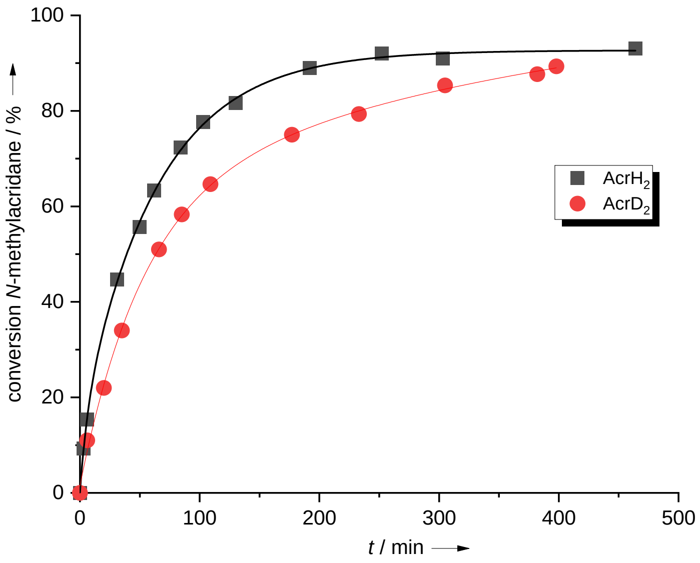

^{1}H NMR Data (CD_3CN)

$AcrH_2$[a]	$AcrD_2$[b]	$AcrH^+$[c]	$AcrD^+$[a]
7.18 (m, 4 H), 6.94 (m, 4 H), 3.85 (s, 2 H), 3.35 (s, 3 H)	7.18 (m, 4 H), 6.94 (m, 4 H), 3.35 (s, 3 H)	9.86 (s, 1 H), 8.53 (m, 4 H), 8.41 (t, 2 H), 7.99 (t, 2 H), 4.76 (s, 3 H)	8.56 (t, 4 H), 8.42 (t, 2 H), 7.99 (t, 2 H), 4.76 (s, 3 H)

[a] 399.89 MHz; [b] 199.87 MHz; [c] 600.13 MHz

8 Experimental details for reaction of 1^{2+} with 9,10-dihydroanthracene (AnH_2)

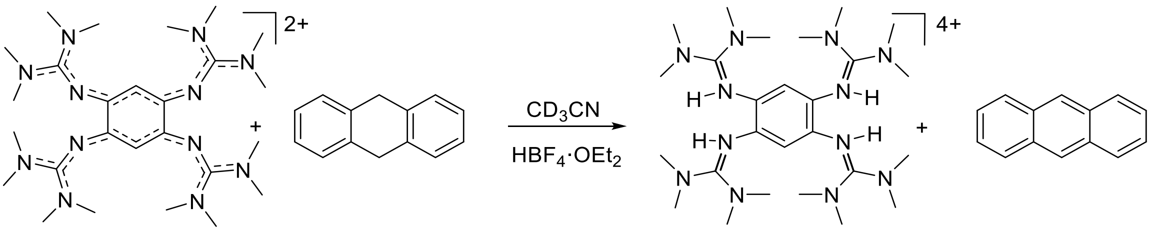

entry	A	B	C
**1**$(PF_6)_2$ [mg/µmol]	8.248/10.05	-	6.838/8.33
**1**$(ClO_4)_2$ [mg/µmol]	-	5.848/8.01	-
AnH_2 [mg/µmol]	2.048/11.36	1.442/8.00	1.514/8.40
$HBF_4 \cdot OEt_2$ (10% in CD_3CN) [µL/µmol/eq. rel. to $\mathbf{1}^{2+}$]	69/50/5	88/64/8	103/75/9
HMB [mg/µmol]	0.938/5.78	1.184/7.30	0.776/4.78
CD_3CN [ml]	0.45	0.45	0.45
reaction time (temperature)	180 min (r.t.)	60 min (r.t.)	90 min (r.t.)
ratio (GFA/substrate)	1/1.1	1/1	1/1
conversion to anthracene (An) [%]	78[a]	62	65

[a] rel. to $(\mathbf{1}+4H)^{4+}$

General protocol (NMR experiments)

In a Schlenk flask $\mathbf{1}^{2+}$, HMB and 9,10-dihydroanthracene were dissolved in 0.45 ml CD_3CN and transferred via syringe into a flame-sealable NMR tube. $HBF_4 \cdot OEt_2$ (10% in CD_3CN, c = 0.729 $mol \cdot l^{-1}$) was added via microliter syringe. The NMR tube was flame-sealed in vacuum at -196 °C.

^{1}H NMR spectrum (199.87 MHz, 298 K, CD_3CN) for entry A: first measurement

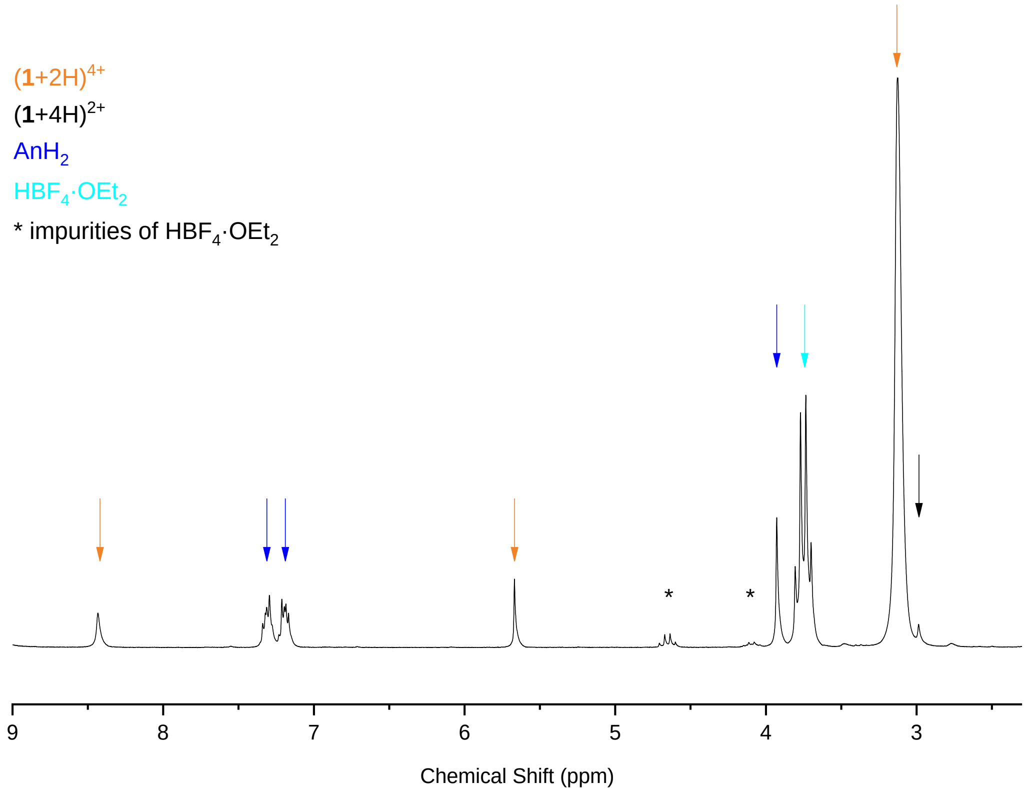

^{1}H NMR spectrum (199.87 MHz, 298 K, CD_3CN) for entry A: measurement after 2 h

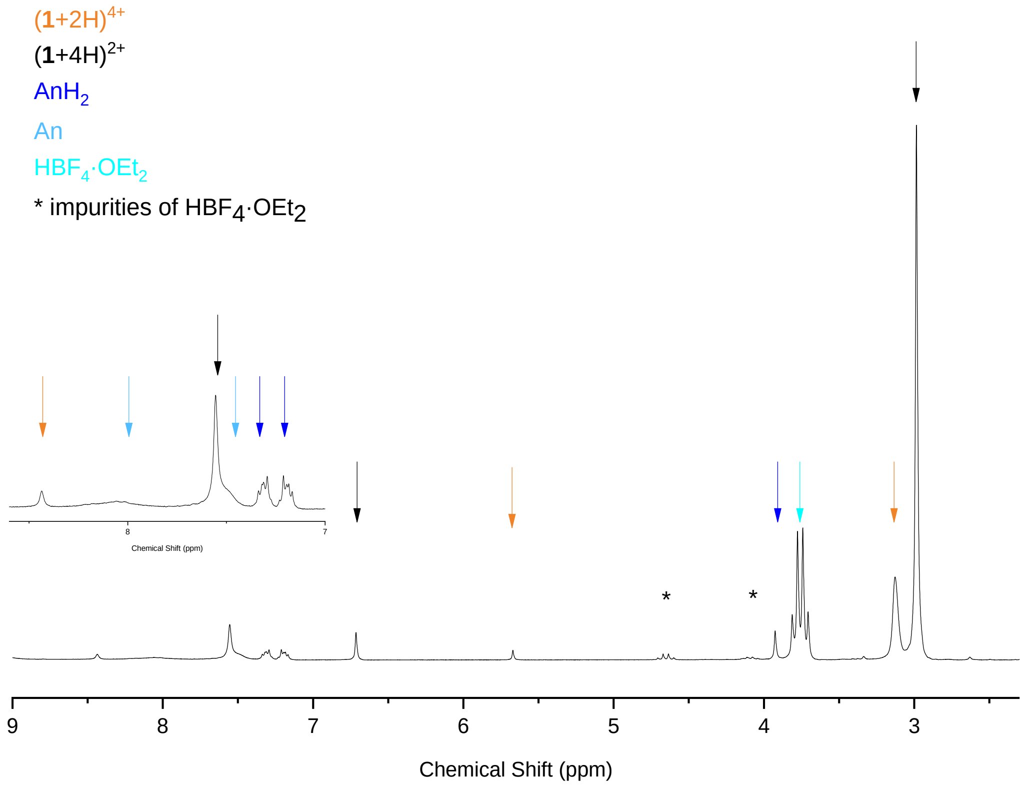

^{1}H NMR spectrum (600.13 MHz, 295 K, CD_3CN) for entry A: last measurement

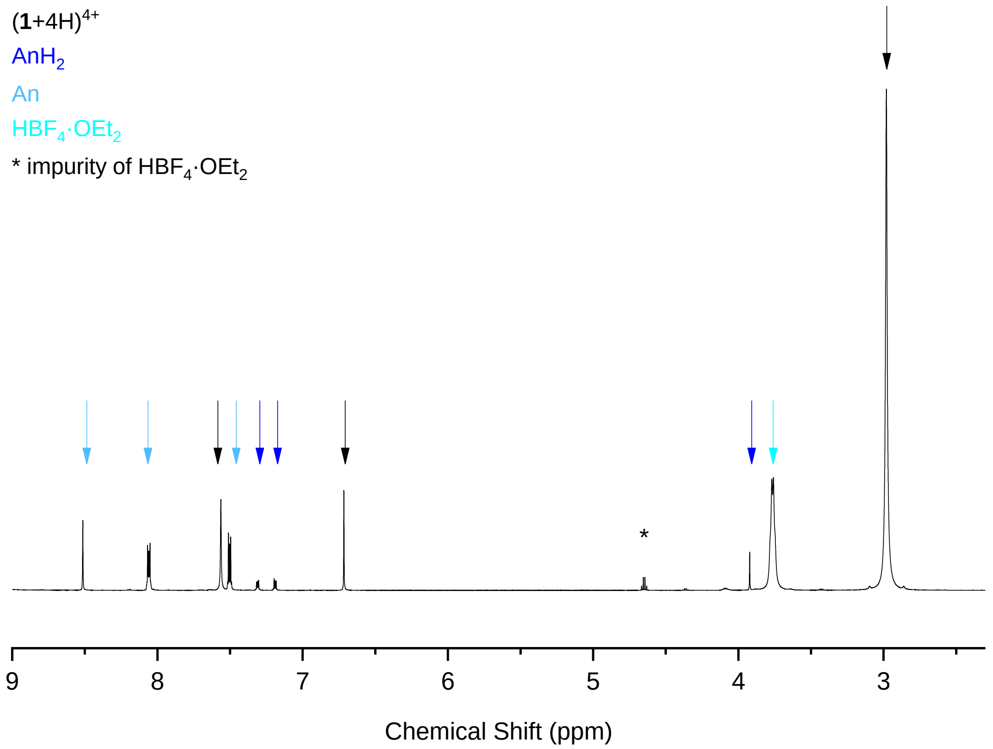

Plot showing the conversion as a function of time for the reaction between $1(PF_6)_2$ and 9,10-dihydroanthracene in the presence of 5 eq. and 9 eq. $HBF_4{\cdot}OEt_2$

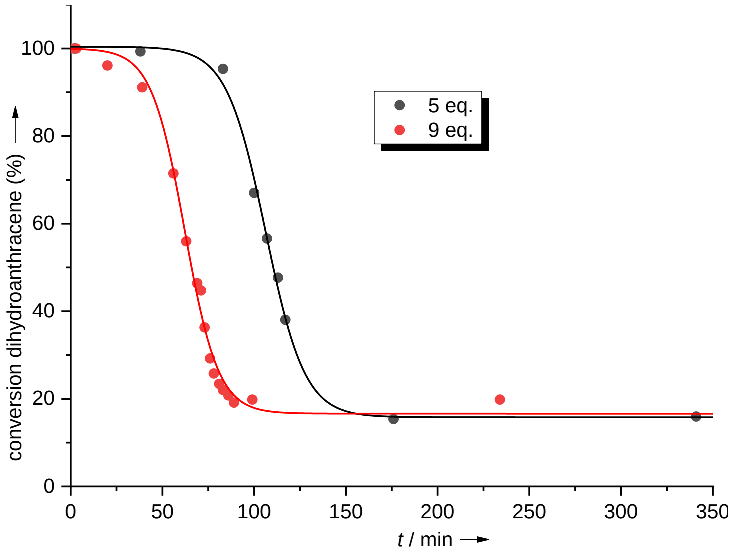

Please note that the conversion in this plot refers to the decay of the dihydroanthracene (AnH_2) signals in the ^{1}H NMR spectrum. By contrast, the conversion to anthracene (NMR yield) given in the tables are determined from the anthracene (An) signal integrals relative to the guanidine signal integrals. The different procedures are necessary due to the formation of the radical $AnH^{\cdot}$ as reaction intermediate, leading to temporary broad product signals not applicable for integration in the course of the reaction.

9 Experimental details for reaction of 1(PF_6)$_2$ with 1-benzyl-1,4-dihydronicotinamide (BNAH)

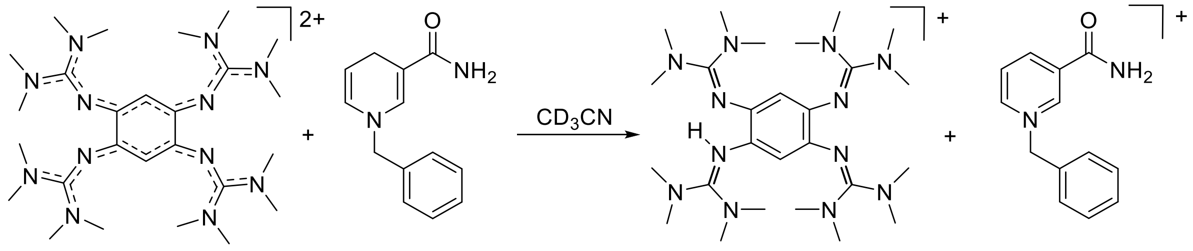

entry	A	B
**1**(PF_6)$_2$ [mg/µmol]	5.314/6.48	4.024/4.90
BNAH [mg/µmol]	1.440/6.72	1.042/4.86
HMB [mg/µmol]	0.822/5.07	0.660/4.07
CD_3CN [ml]	0.45	0.45
reaction time (temperature)	24 h (60 °C)	24 h (40 °C)
ratio (GFA/substrate)	1/1	1/1
conversion to $BNAH^+$ [rel. to $\mathbf{1}^{2+}$/($\mathbf{1}$+nH)$^{n+}$, %]	27	24

General protocol (NMR experiments)

In a Schlenk flask **1**(PF_6)$_2$, HMB and BNAH were dissolved in 0.45 ml CD_3CN and transferred via syringe into a flame-sealable NMR tube. The NMR tube was flame-sealed in vacuum at -196 °C. The tube was heated to 60 °C or in case of entry **B** to 40 °C.

^{1}H NMR spectrum (199.87 MHz, 298 K, CD_3CN) for entry A: first measurement

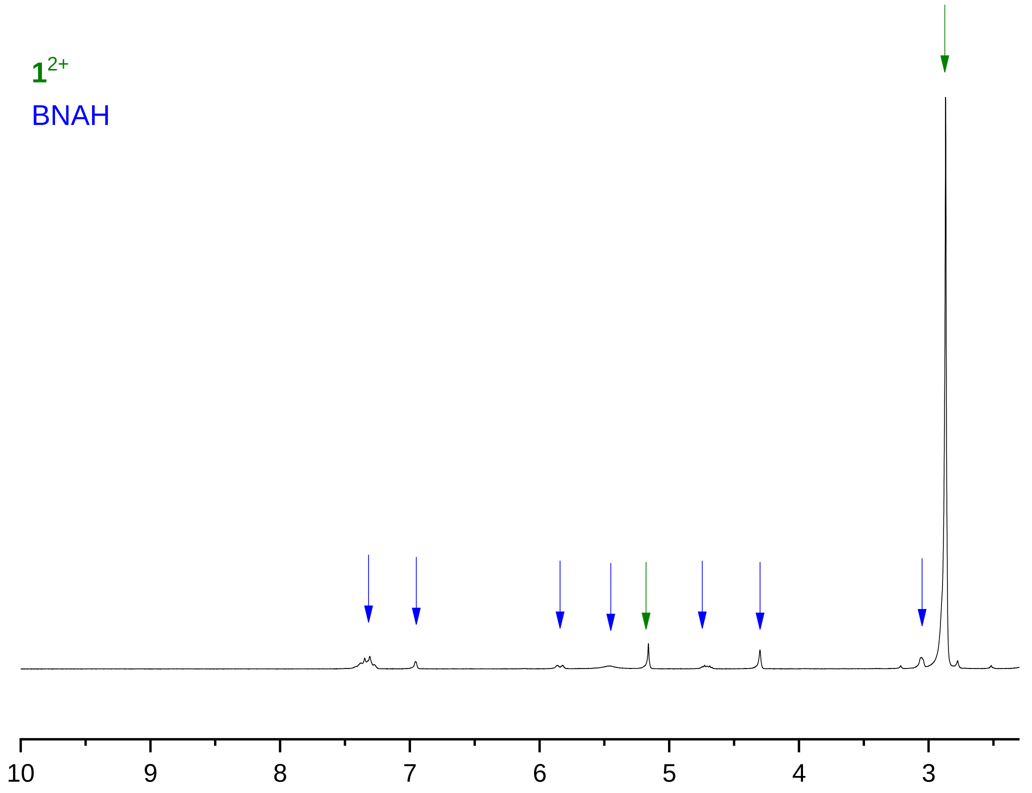

^{1}H NMR spectrum (199.87 MHz, 298 K, CD_3CN) for entry A: measurement after 24 h

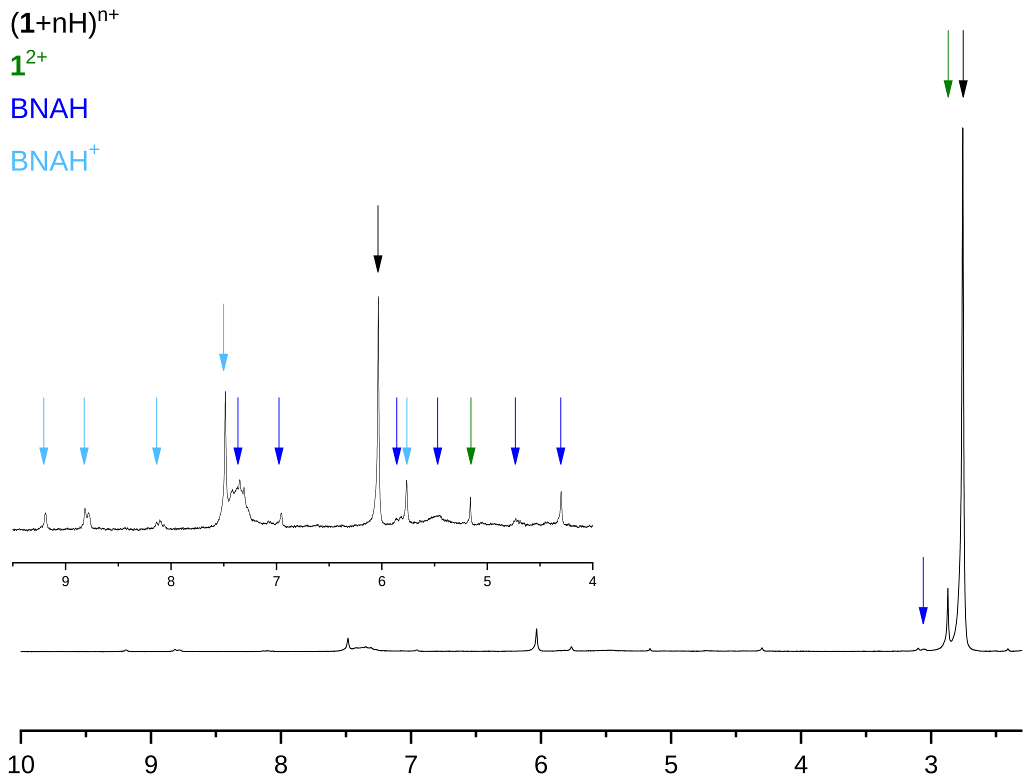

Prolonged reaction times (2 d) lead to complete consumption of $\mathbf{1}^{2+}$, but also to degradation of $BNAH^+$.

^{1}H NMR spectrum (199.87 MHz, 298 K, CD_3CN) for entry A: measurement after 2d

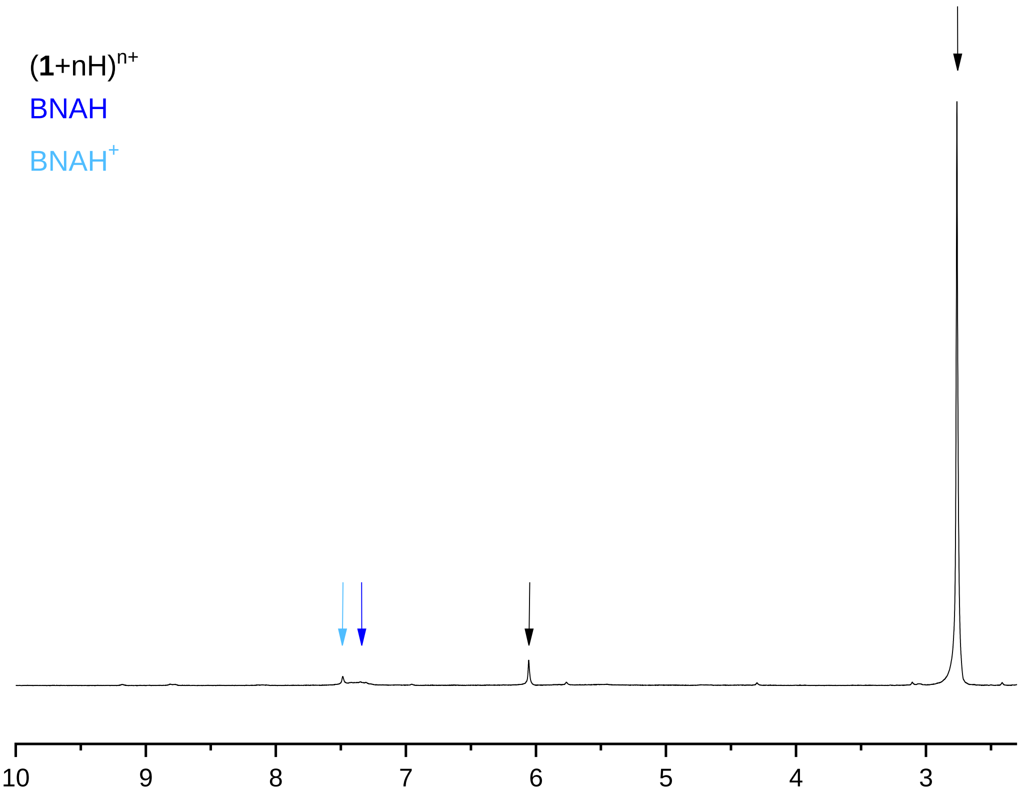

10 Experimental details for the PCET reaction between $2(PF_6)_2$ and $(1{+}2H)(PF_6)_2$

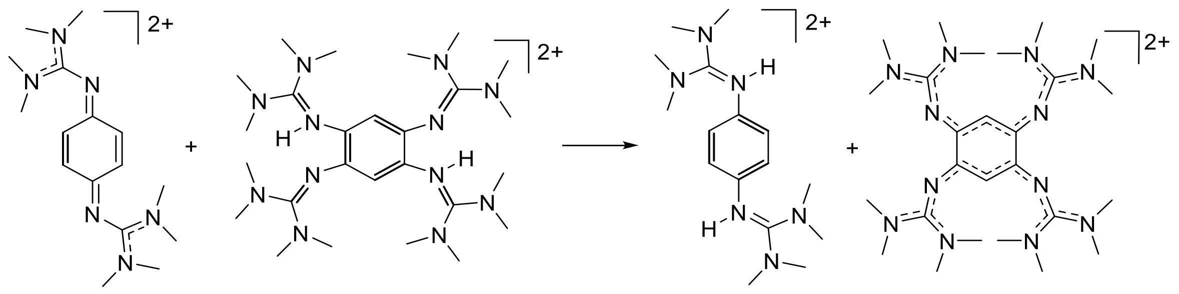

UV-vis experiments

Three solutions were prepared (solution I – III, see Table below). Under argon atmosphere a 1 cm quartz glass cuvette was filled with 3.0 ml of solution I. Under stirring 9.3 µl of solution II was added. For the reaction in entry B, 3.8 µl of solution III was also added. The conversion was followed by UV-vis spectroscopy.

	**solution I**	**solution II**	**solution III**
	**2**$(PF_6)_2$	(**1**+2H)$(PF_6)_2$	$HBF_4 \cdot OEt_2$
[mg/mmol]	$0.278/4.68 \cdot 10^{-4}$	$24.876/3.02 \cdot 10^{-2}$	$1.19/7.35^{-3}$
CH_3CN [ml]	25	5	2
c [$mol \cdot l^{-1}$]	$1.87 \cdot 10^{-5}$	$6.05 \cdot 10^{-3}$	$3.67 \cdot 10^{-3}$

**entry**	**A**	**B**
solution I [ml/mmol]	$3/5.61 \cdot 10^{-5}$	$3/5.61 \cdot 10^{-5}$
solution II [µl/mmol]	$9.3/5.62 \cdot 10^{-5}$	$9.3/5.62 \cdot 10^{-5}$
solution III [µl/mmol]		$3.8/1.4 \cdot 10^{-5}$
ratio **2**$^{2+}$/(**1**+2H)$^{2+}$	1/1	1/1
ratio **2**$^{2+}$/ $HBF_4 \cdot OEt_2$		1/0.25

UV-vis spectra for entry A
(without $HBF_4 \cdot OEt_2$)

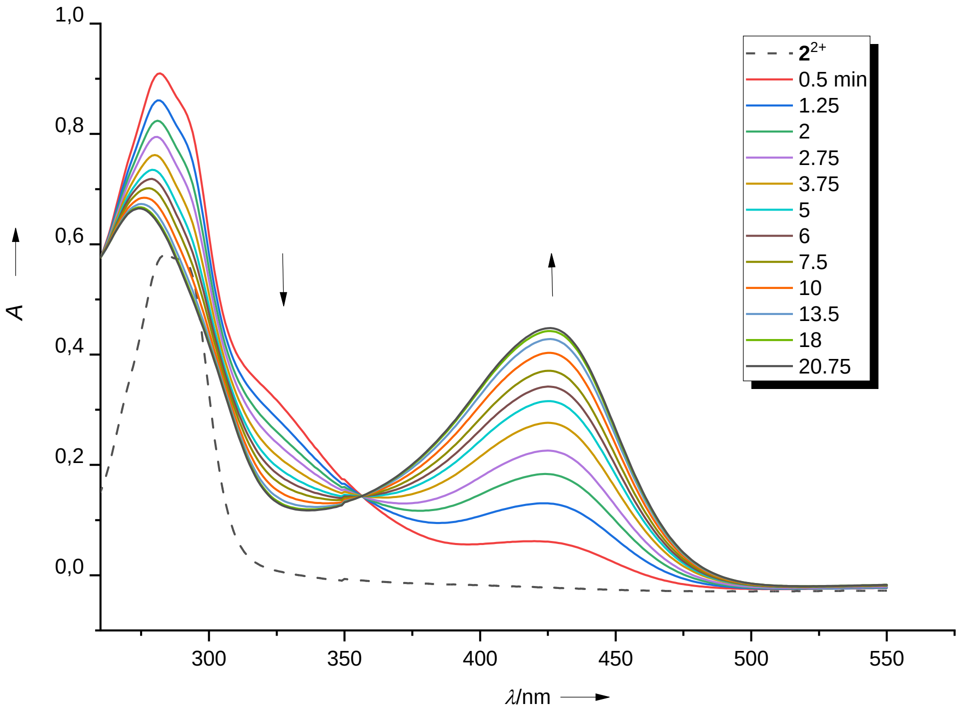

UV-vis spectra for entry B
(with $HBF_4 \cdot OEt_2$)

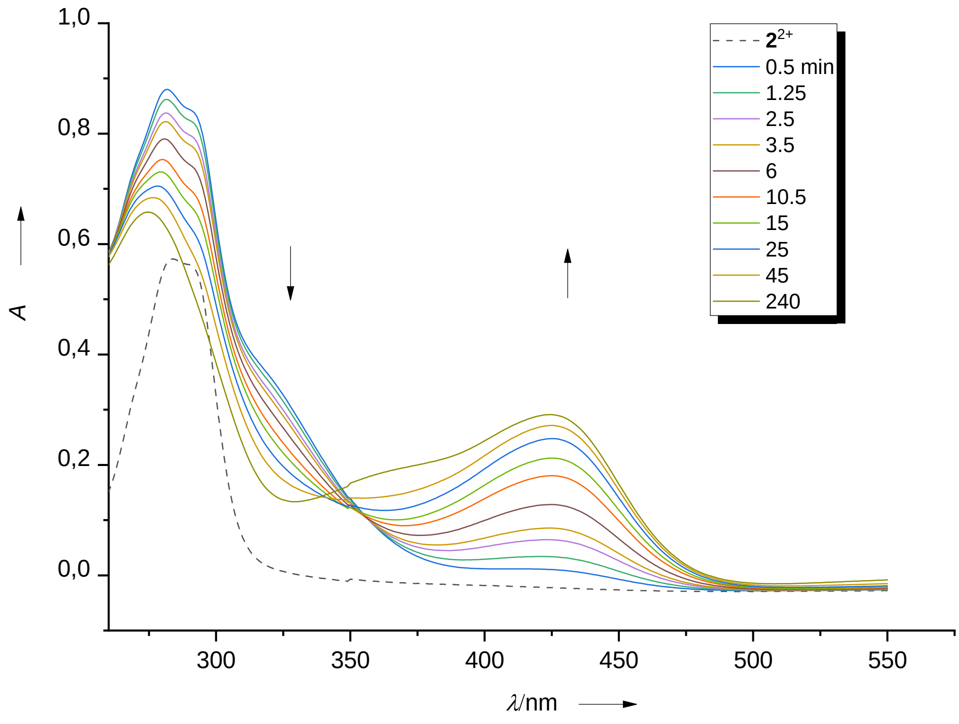

Plot of the absorptions at 420 nm (band of the product 1^{2+}) and 325 nm (dominating contribution from the reactant 2^{2+}) in dependence of the reaction time

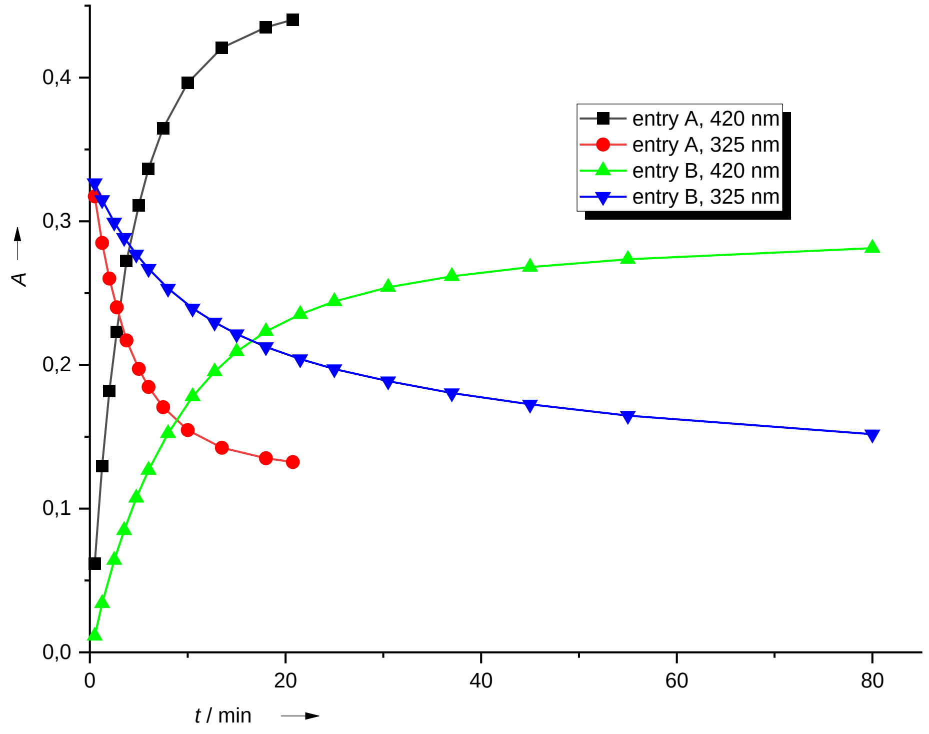

NMR experiments for the reaction of 2(PF_6)$_2$ with (1+2H)(PF_6)$_2$

Protocol: The two reactants, 5.94 mg (0.01 mmol) of **2**(PF_6)$_2$ and 8.22 mg (0.01 mmol) of (**1**+2H)(PF_6) were weighed into an NMR tube and dissolved in 8 ml CD_3CN ($c = 1.25 \cdot 10^{-2}$ mol·l^{-1}). The reaction was followed by ^1H NMR spectroscopy. Already the first measurements showed quantitative conversion.

^1H NMR spectrum (199.87 MHz, 298 K, CD_3CN) for the reaction between 2(PF_6)$_2$ and (1+2H)(PF_6)$_2$: first measurement after 5 min at 298 K

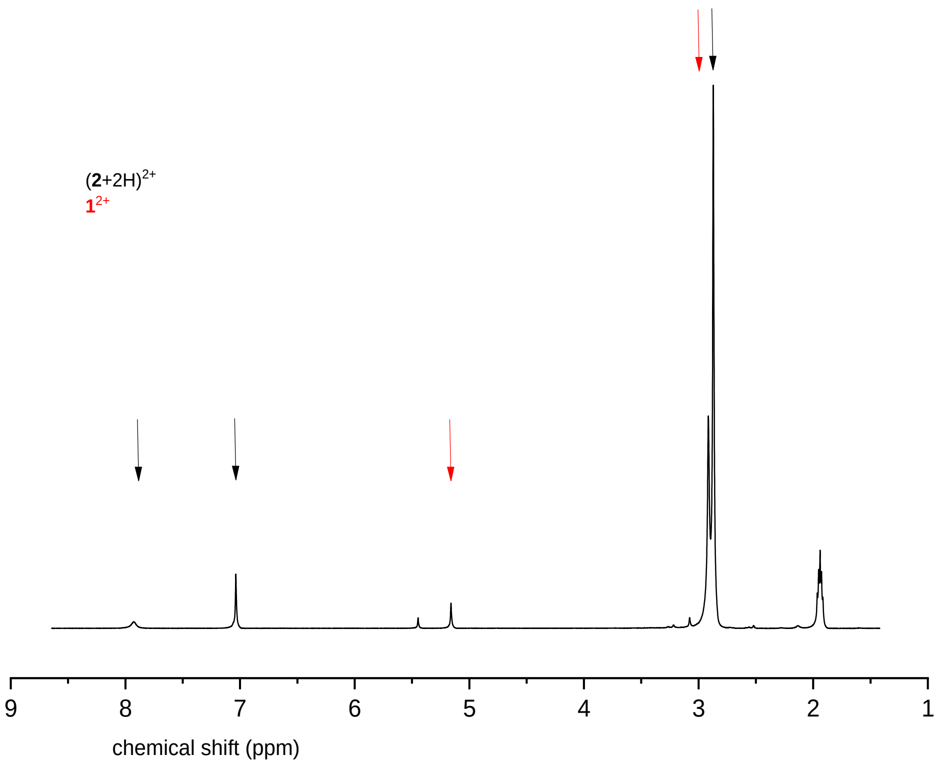

Experiments with different concentrations of $HBF_4{\cdot}OEt_2$

After completion of the reaction, 3-5 µl portions of a solution of $HBF_4{\cdot}OEt_2$ (0.12 g·ml^{-1} in CD_3CN, c = 7.3·10^{-1} mol·l^{-1}) were added. The ratio **2**$^{2+}$/ $HBF_4{\cdot}OEt_2$ was estimated by NMR spectroscopy. An excess of $HBF_4{\cdot}OEt_2$ leads to formation of the twofold-protonated, oxidized compound (**1**+2H)$^{4+}$, but not to PCET.

^{1}H NMR spectra (199.87 MHz, 298 K, CD_3CN)

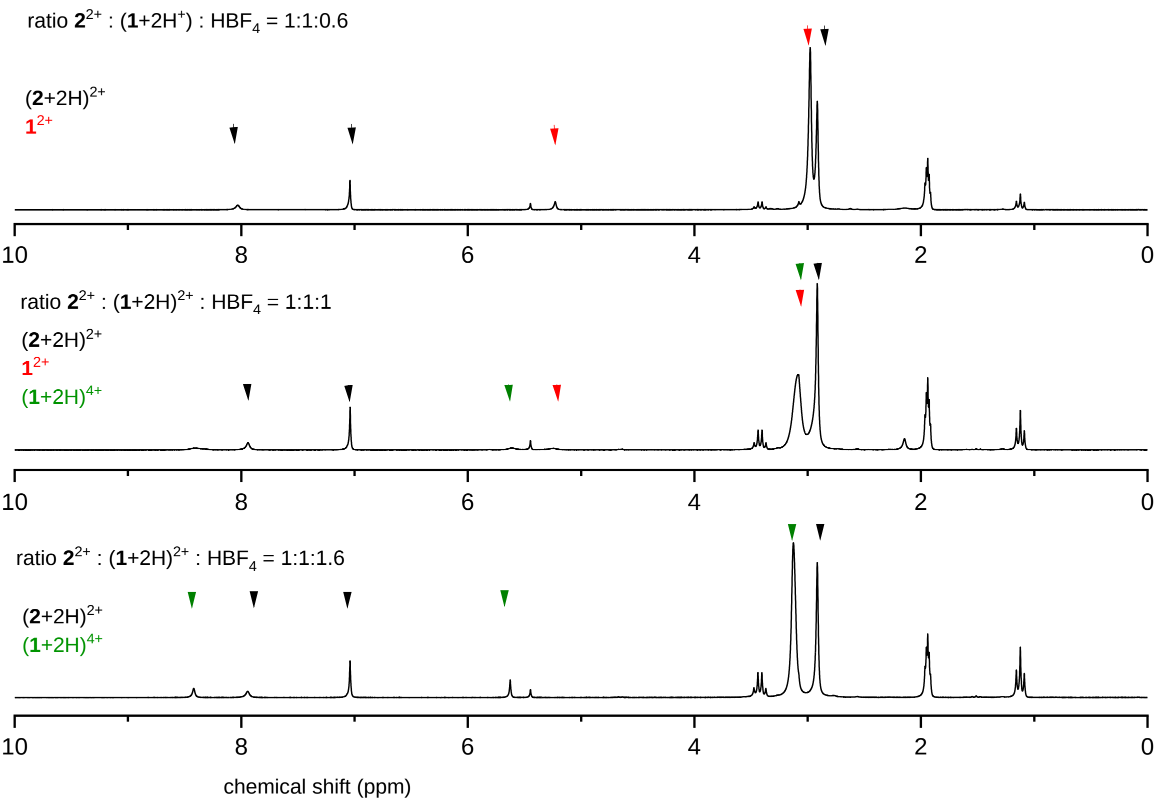

Stopped flow UV-vis measurements:

First, stock solutions of (**1**+2H)(PF_6)$_2$ (solution I) and **2(**PF_6)$_2$ (solution II) were prepared. By stepwise dilution of solution II, three further solutions (III-V) of **2(**PF_6)$_2$ with decreasing concentrations were obtained.

	**solution I**	**solution II**
	(**1**+2H)(PF_6)$_2$	**2**(PF_6)$_2$
[mg/mmol]	$0.660/8.02 \cdot 10^{-4}$	$18.698/3.15 \cdot 10^{-2}$
CH_3CN [ml]	10	10
c [$mol \cdot l^{-1}$]	$8.02 \cdot 10^{-5}$	$3.15 \cdot 10^{-3}$

	**solution III**	**solution IV**	**solution V**
solution [ml]	solution II [5]	solution III [5]	solution IV [1]
CH_3CN [ml]	5	5	9
c [$mol \cdot l^{-1}$]	$1.57 \cdot 10^{-3}$	$7.87 \cdot 10^{-4}$	$7.87 \cdot 10^{-5}$

Then the two storage vessels of the instrument were charged with solution I and one of the solutions II-V, and the measuring cell filled with equal volumes of both solutions. The following concentrations and molar ratios between the two reactants result.

**entry**	**A**	**B**	**C**	**D**
solution / c [$mol \cdot l^{-1}$]	II/$1.57 \cdot 10^{-3}$	III/$7.87 \cdot 10^{-4}$	IV/$3.93 \cdot 10^{-4}$	V/$3.93 \cdot 10^{-5}$
solution I c [$mol \cdot l^{-1}$]	$4.01 \cdot 10^{-5}$	$4.01 \cdot 10^{-5}$	$4.01 \cdot 10^{-5}$	$4.01 \cdot 10^{-5}$
ratio	1 : 39	1 : 19.6	1 : 9.8	1 : 0.98

First order plots of the absorption at 420 nm in dependence of the reaction time for the initial stage of the reaction between (**1**+2H)$(PF_6)_2$ and an excess of **2**$(PF_6)_2$. The pseudo-first order rate constants k_{obs} result from linear fits.

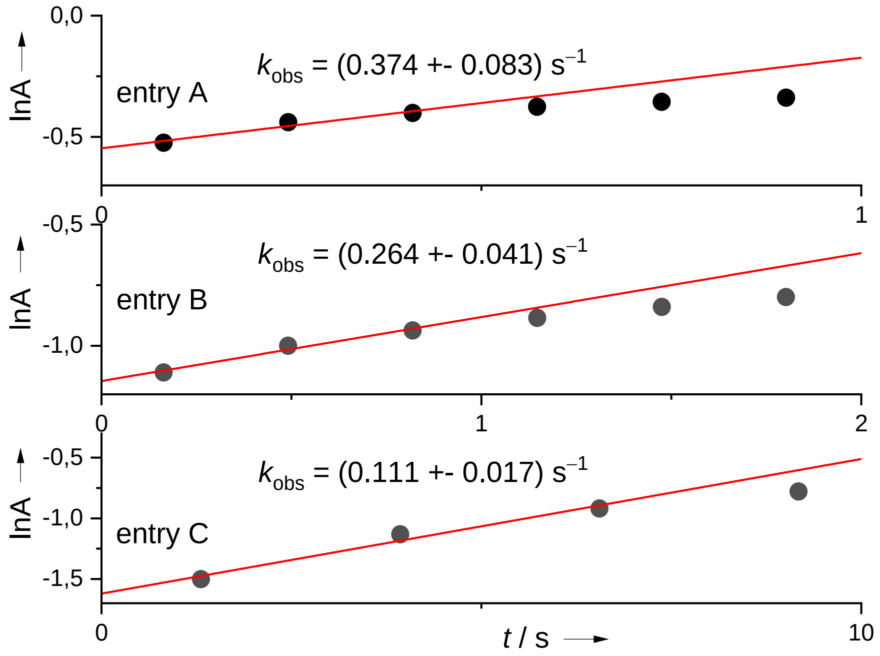

Plot of the k_{obs} values as a function of the concentration of **2**$^{2+}$ in the solution. From a linear fit the second order rate constant is estimated to be $k_H = 257 \pm 27\ M^{-1}s^{-1}$ at room temperature.

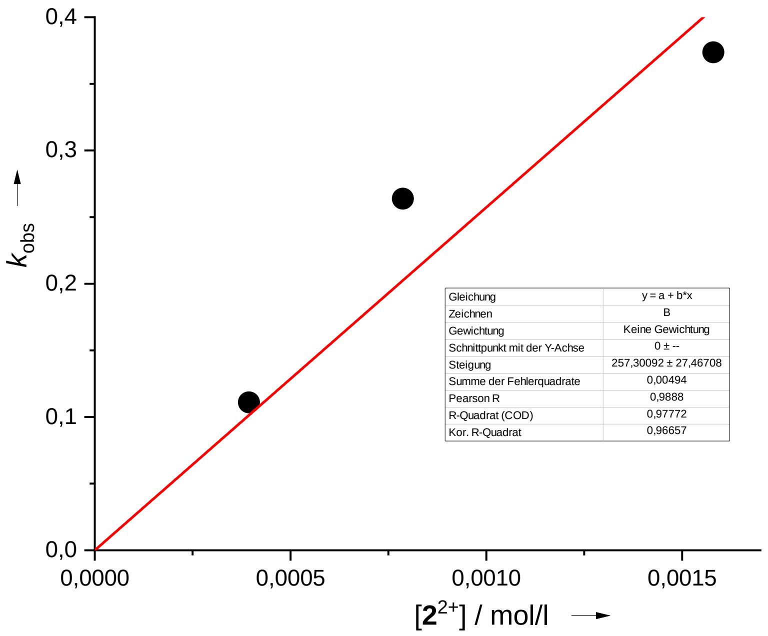

11 Experimental details for reaction of 2(PF_6)$_2$ with 1-benzyl-1,4-dihydronicotinamide (BNAH)

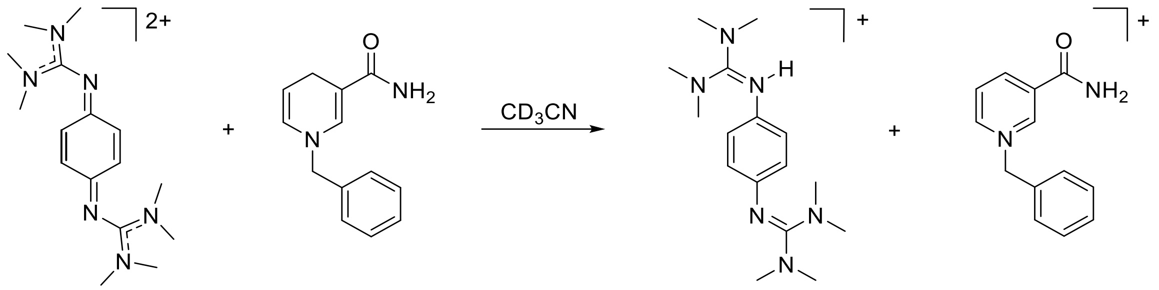

entry	A
**2**(PF_6)$_2$ [mg/µmol]	3.924/6.60
BNAH [mg/µmol]	1.42/6.63
HMB [mg/µmol]	0.856/5.28
CD_3CN [ml]	0.45
reaction time (temperature)	< 1 min (r.t.)
ratio (GFA/substrate)	1/1
conversion to $BNAH^+$ [%]	>99

General protocol (NMR experiments)

In a Schlenk flask **2**(PF_6)$_2$, HMB and BNAH were dissolved in 0.45 ml CD_3CN and transferred via syringe into a flame-sealable NMR tube. The NMR tube was flame-sealed in vacuum at -196 °C.

^{1}H NMR spectrum (600.13 MHz, 294.9 K, CD_3CN) for entry A

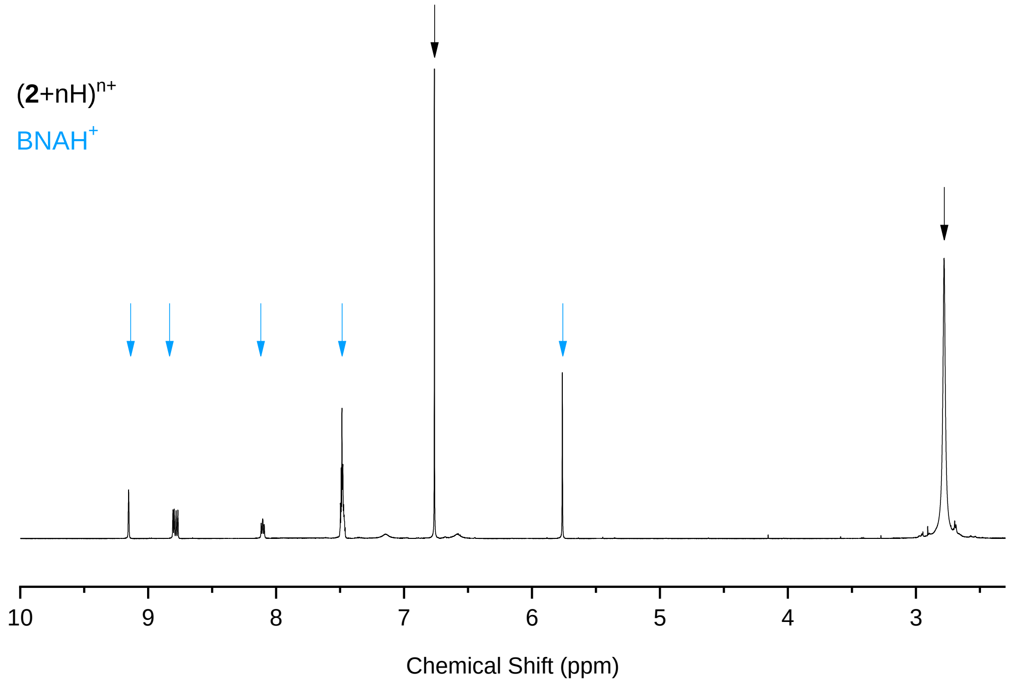

^{1}H NMR of BNAH$^+$: (600.13 MHz, 294.9 K, CD_3CN): δ = 5.76 (s, 2 H), 7.49 (m, 5 H), 8.10 (dd, *J* = 7.69, 6.52 Hz, 1 H), 8.79 (dd, 15.99, 7.13 Hz, 2 H), 9.15 (s, 1 H) ppm.

12 Experimental details for reaction of 2(PF_6)$_2$ with 10-methyl-9,10-dihydroacridine ($AcrH_2$)

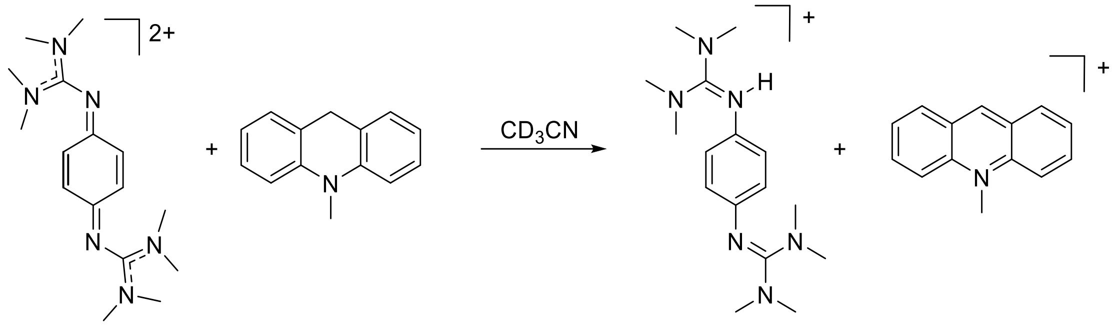

entry	A	B	C
**2**(PF_6)$_2$ [mg/µmol]	7.76/13.06	3.912/6.58	3.568/6.00
$AcrH_2$ [µmol]	6.45	6.45	6.45
HMB [mg/µmol]	0.598/3.69	1.06/6.53	0.914/5.63
CD_3CN [ml]	0.45	0.45	0.45
reaction time (temperature)	160 min (r.t.)	180 min (r.t.)	180 min (r.t.)
ratio (GFA/substrate)	1/0.5	1/1	1/1.1
conversion to $AcrH^+$ [%]	92[b]	73	74[a]

[a] rel. to (**2**+nH)$^{n+}$
[b] rel. to applied $AcrH_2$

General protocol (NMR experiments)

Under an argon atmosphere 0.25 ml of a solution of 10-methyl-9,10-dihydroacridine (c = 0.0258 mol·l^{-1} in CH_3CN) was transferred into a flame-sealable NMR tube and the solvent was removed under high-vacuum. In a Schlenk flask **2**(PF_6)$_2$ and HMB were dissolved in 0.45 ml CD_3CN and added via syringe to the 10-methyl-9,10-dihydroacridine. The NMR tube was flame-sealed in vacuum at -196 °C.

^1H NMR spectrum (200.13 MHz, 298 K, CD_3CN) for entry A: first measurement

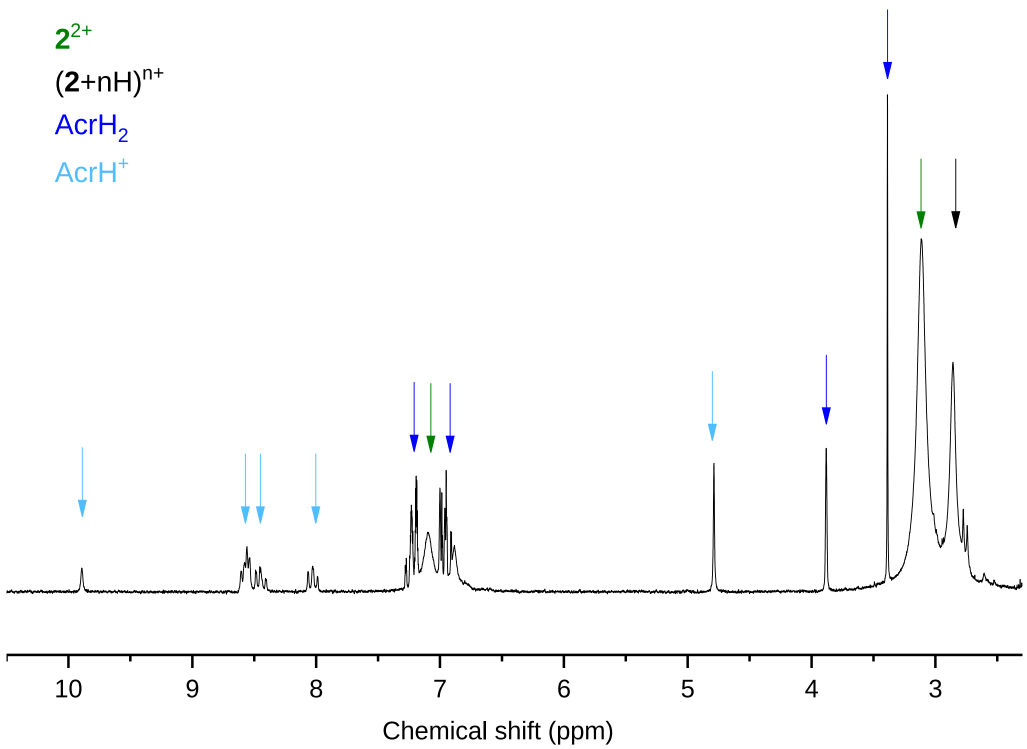

^1H NMR spectrum (200.13 MHz, 298 K, CD_3CN) for entry A: last measurement

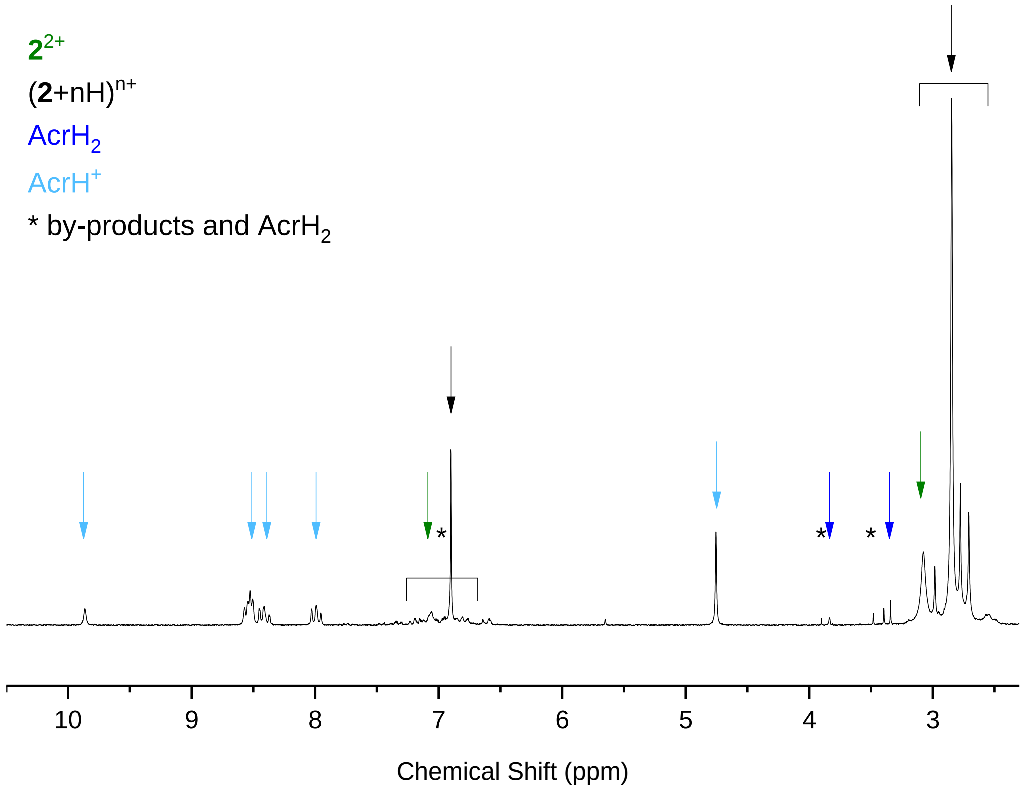

^{1}H NMR spectrum (199.87 MHz, 298 K, CD_3CN) for entry B: last measurement (after 3 h)

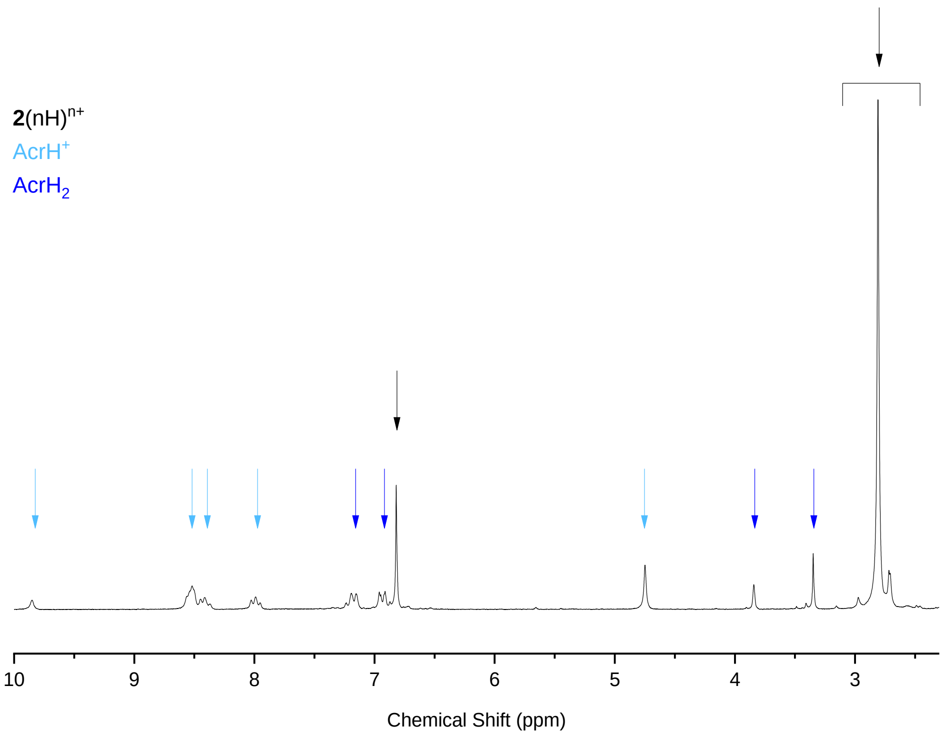

^{1}H NMR spectrum (600.13 MHz, 295 K, CD_3CN) for entry B: measurement after 7.5 h

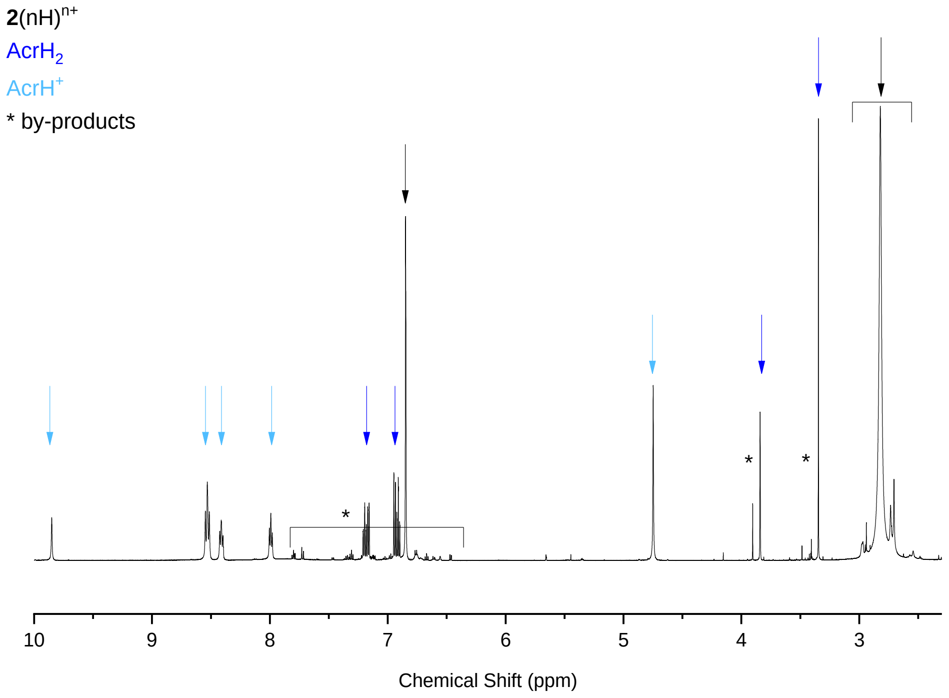

The NMR spectrum recorded after 180 min still shows the presence of $AcrH_2$. Hence one might expect higher conversion for longer reaction times. However, prolonged reaction times led to the appearance of signals due to unidentified by-products, without further $AcrH_2$ conversion.

13 Experimental details for reaction of 2(PF$_6$)$_2$ with 10-methyl-[9,9'-^{2}H$_2$]-acridine (AcrD$_2$)

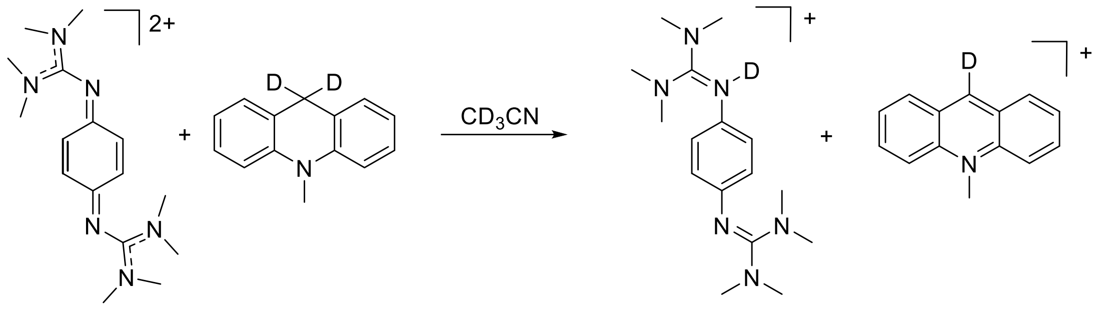

entry	A	B
**2**(PF$_6$)$_2$ [mg/µmol]	6.862/11.55	3.168/5.33
AcrD$_2$ [µmol]	6.50	6.50
HMB [mg/µmol]	0.436/2.69	0.708/4.36
CD$_3$CN [ml]	0.45	0.45
reaction time (temperature)	460 min (r.t.)	400 min (r.t.)
ratio (GFA/substrate)	1/0.6	1/1.2
conversion to AcrD$^+$ [%]	80[b]	59[a]

[a] rel. to (**2**+nD)$^{n+}$
[b] rel. to applied AcrD$_2$

General protocol (NMR experiments)

Under an argon atmosphere 0.25 ml of a solution of 10-methyl-[9,9'-^{2}H$_2$]-acridine (c = 0.026 mol·l^{-1} in CH$_3$CN) was transferred into a flame-sealable NMR tube and the solvent was removed under high-vacuum. In a Schlenk flask **2**(PF$_6$)$_2$ and HMB were dissolved in 0.45 ml CD$_3$CN and added via syringe to the 10-methyl-[9,9'-^{2}H$_2$]-acridine. The NMR tube was flame-sealed in vacuum at -196 °C.

^{1}H NMR spectrum (199.87 MHz, 298 K, CD_3CN) for entry A: first measurement

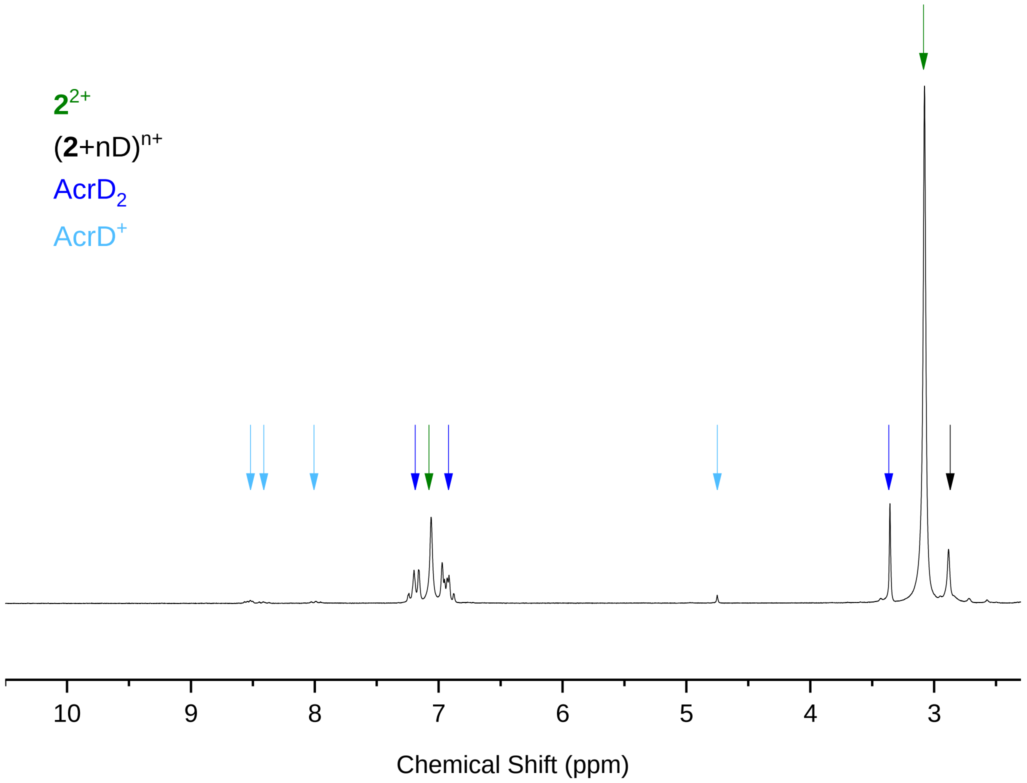

^{1}H NMR spectrum (399.89 MHz, 295.3 K, CD_3CN) for entry A: last measurement

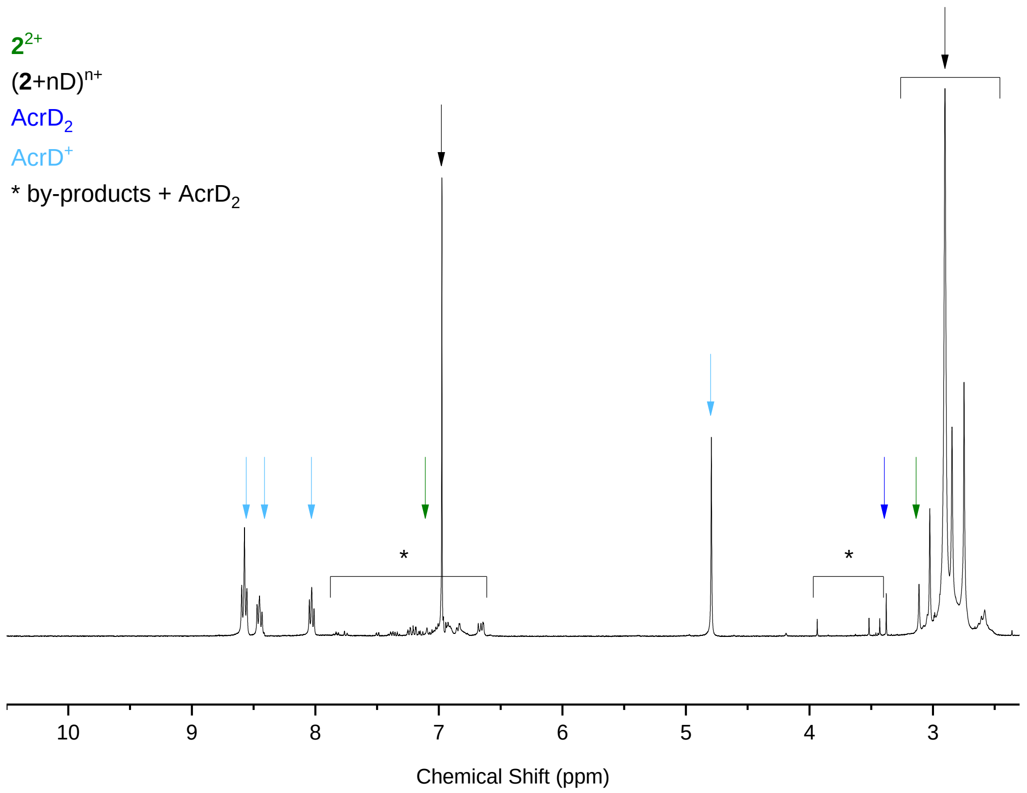

^{1}H NMR spectrum (199.87 MHz, 298 K, CD_3CN) for entry B: last measurement

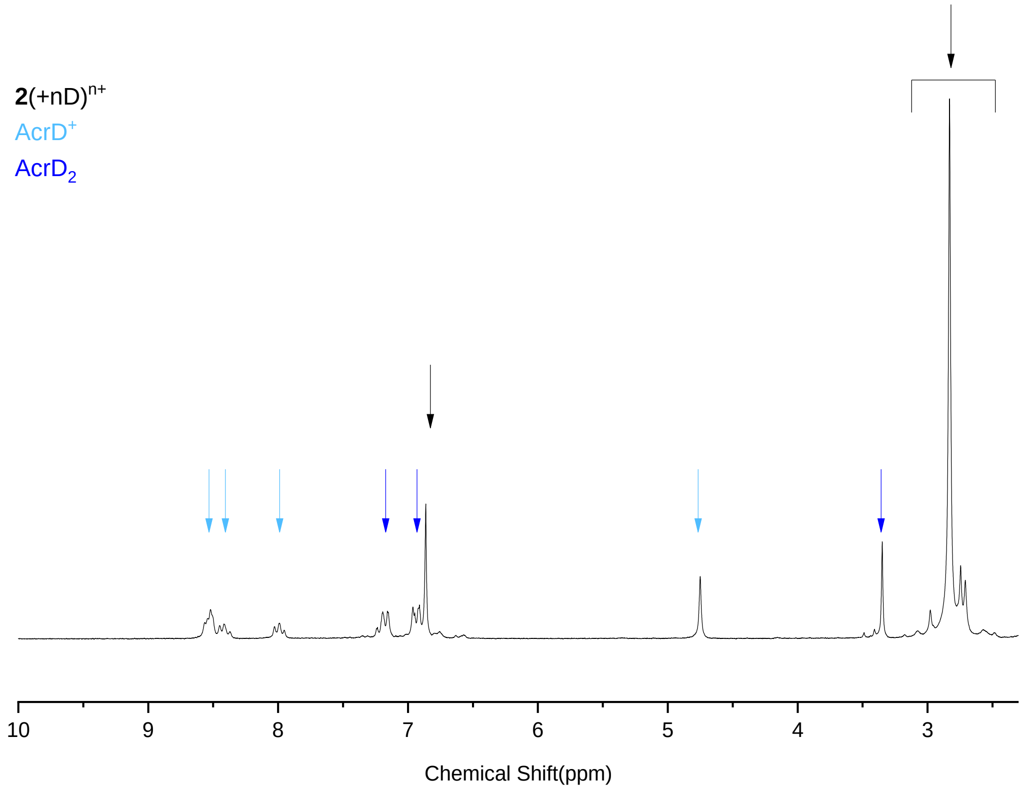

14 Experimental details for reaction of 2(PF_6)$_2$ with 9,10-dihydroanthracene (AnH_2)

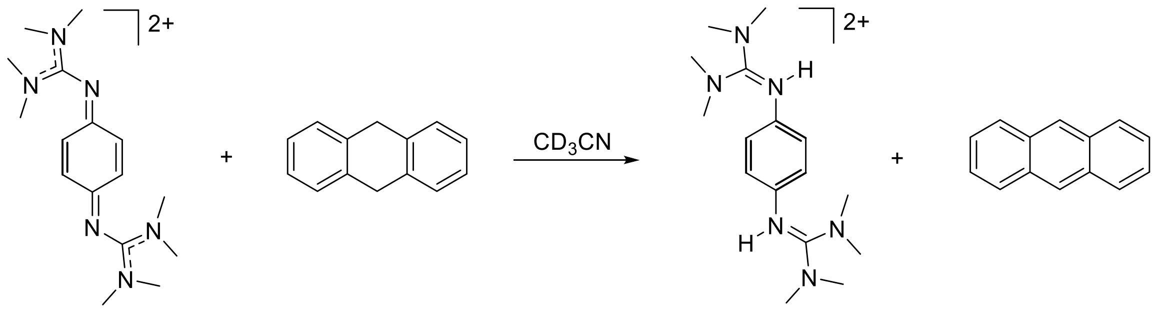

entry	A	B
**2**(PF_6)$_2$ [mg/µmol]	5.052/8.50	4.676/7.87
AnH_2 [mg/µmol]	1.994/11.06	1.550/8.60
NH_4PF_6 [mg/µmol]	-	2.5/15.34
HMB [mg/µmol]	0.808/4.98	1.006/6.20
CD_3CN [ml]	0.45	0.45
reaction time (temperature)	50 h (60 °C)	50 h (60 °C)
ratio (GFA/substrate)	1/1.3	1/1.1
conversion to anthracene (An) [rel. to (**2**+2H)$^{2+}$/**2**$^{2+}$, %]	6	29

General protocol (NMR experiments)

In a Schlenk flask **2**(PF_6)$_2$, HMB and 9,10-dihydroanthracene (and NH_4PF_6 for entry **B**) were dissolved in 0.45 ml CD_3CN and transferred via syringe into a flame-sealable NMR tube. The NMR tube was flame-sealed in vacuum at -196 °C.

^{1}H NMR spectrum (199.87 MHz, 298 K, CD_3CN) for entry A: first measurement

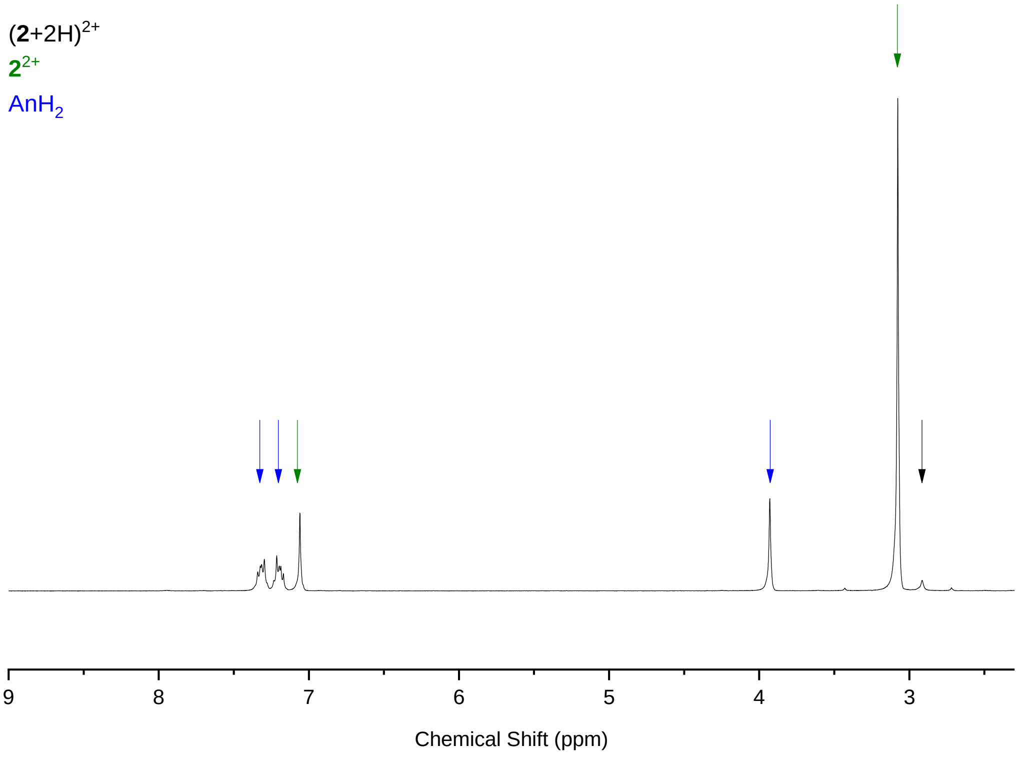

^{1}H NMR spectrum (199.87 MHz, 298 K, CD_3CN) for entry A: last measurement

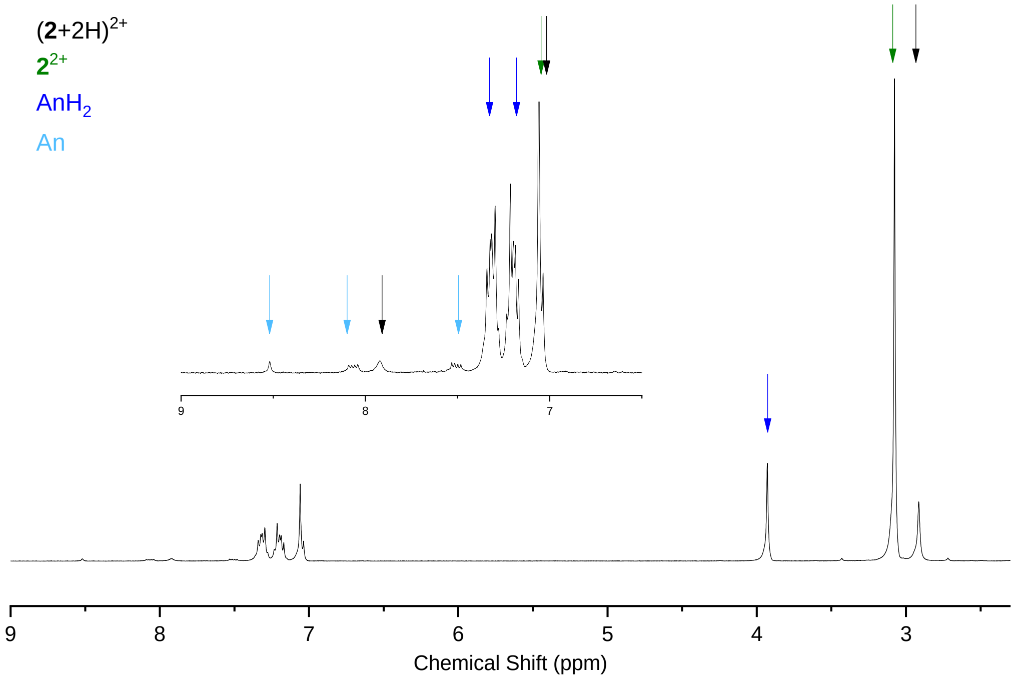

^{1}H NMR of anthracene: (199.87 MHz, 298 K, CD_3CN): δ = 7.51 (dd, J = 6.6, 3.2 Hz, 4 H), 8.06 (dd, J = 6.3, 3.9 Hz, 4 H), 8.52 (s, 2 H) ppm.

^{1}H NMR spectrum (199.87 MHz, 298 K, CD_3CN) for entry B: first measurement

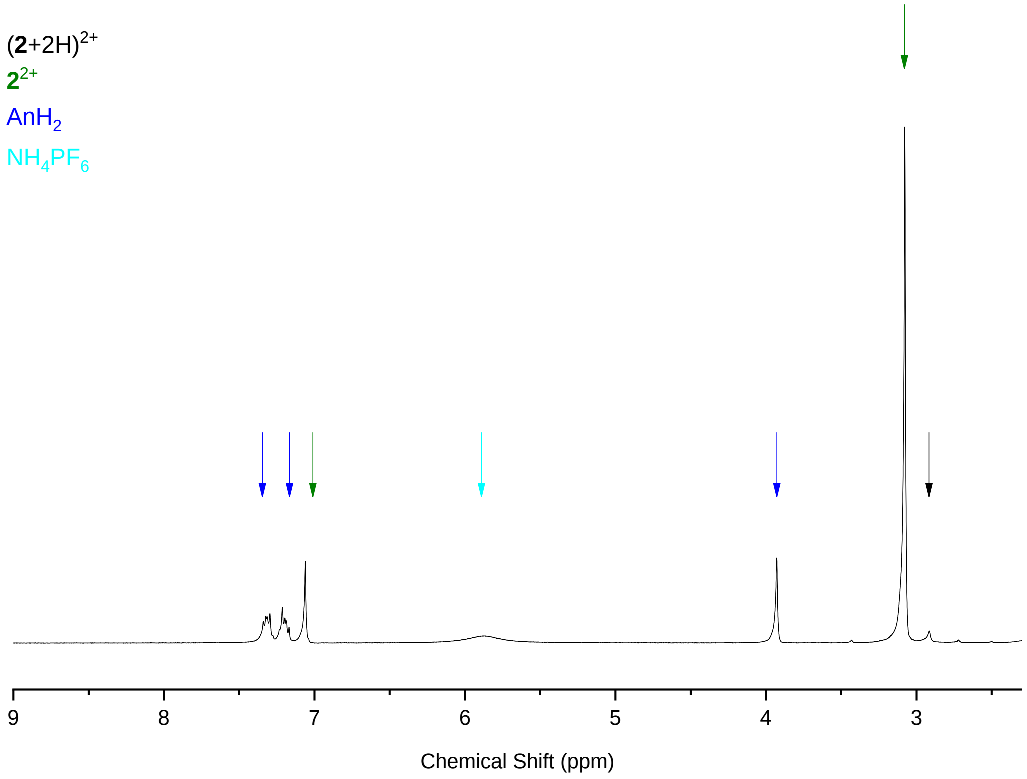

^{1}H NMR spectrum (199.87 MHz, 298 K, CD_3CN) for entry B: last measurement

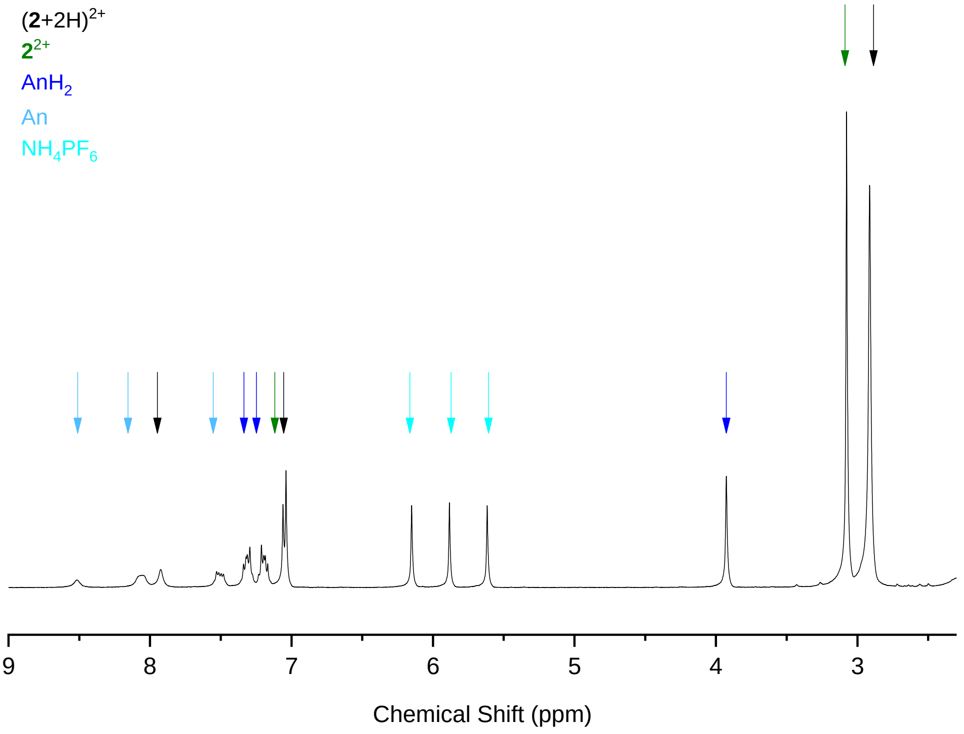

Conversion vs. time plot for the reaction between $2(PF_6)_2$ and 9,10-dihydroanthracene (AnH_2)

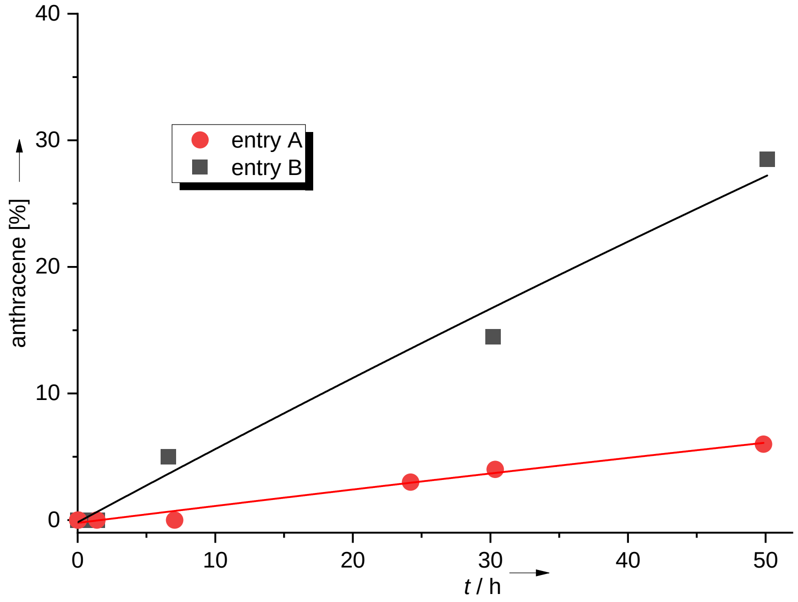

15 Experimental details for reaction of 2(PF_6)$_2$ with 3,3‘‘,4,4‘‘-tetramethoxy-*o*-terphenyl (TMTP) to 2,3,10,11-tetramethoxy-triphenylene

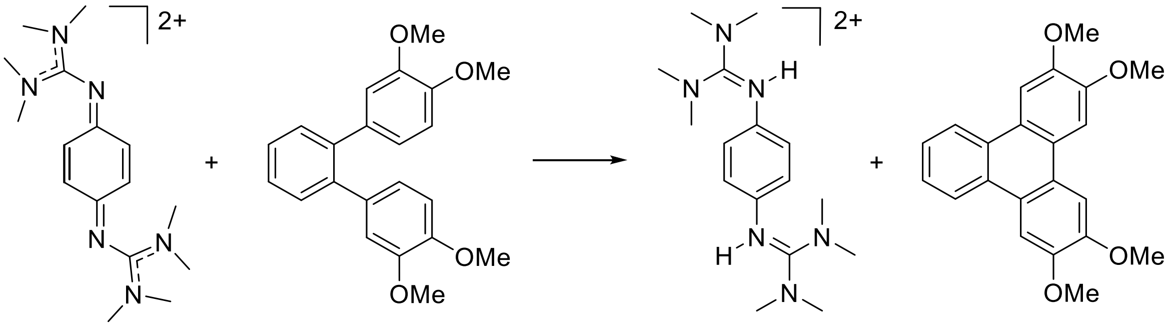

a) Without $HBF_4{\cdot}OEt_2$

entry	A
**2**(PF_6)$_2$ [mg/µmol]	3.108/5.23
3,3‘‘,4,4‘‘-tetramethoxy-*o*-terphenyl [mg/µmol]	1.824/5.21
CD_3CN [ml]	0.5
reaction time (temperature)	60 min (333 K)
ratio (GFA/substrate)	1/1
conversion to 2,3,10,11-tetramethoxy-triphenylene [%]	0.01

General protocol (NMR experiments)

In an NMR tube, the two reactants **2**(PF_6)$_2$ and 3,3‘‘,4,4‘‘-tetramethoxy-*o*-terphenyl were dissolved in CD_3CN. After addition of $HBF_4{\cdot}OEt_2$ (0.12 g·ml^{-1} in CD_3CN), conversion was followed by ^1H NMR spectroscopy.

^{1}H NMR spectrum for entry A (199.87 MHz, 298 K, CD_3CN) for 2(PF_6)$_2$ + 3,3‘‘,4,4‘‘-tetramethoxy-*o*-terphenyl in the absence of HBF_4·OEt_2, after 60 min, 333 K

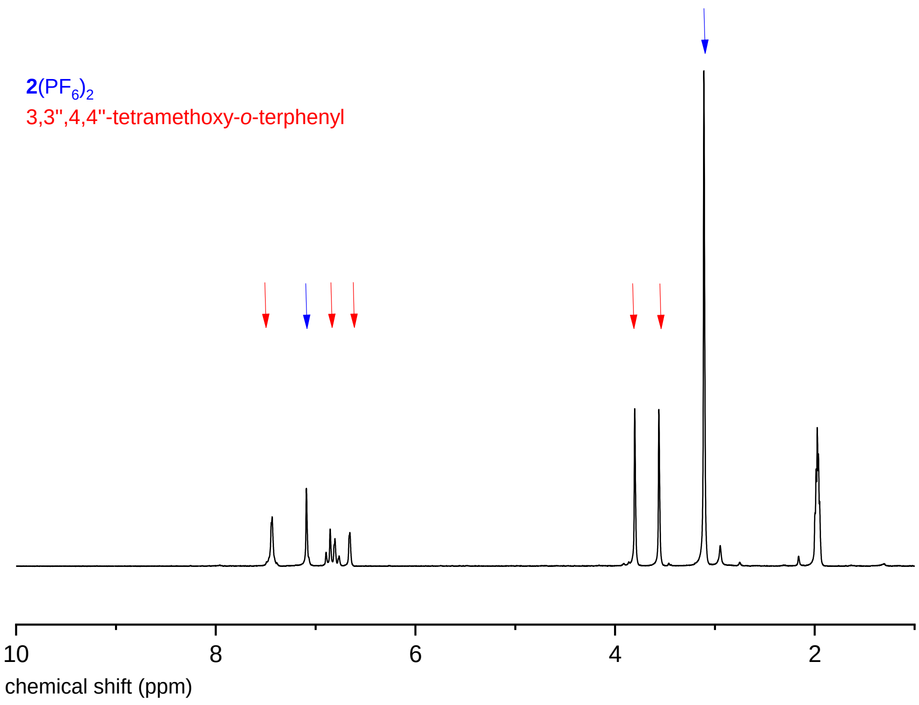

b) With $HBF_4{\cdot}OEt_2$

Experiments at different temperatures

	**B**	**C**
**2**$(PF_6)_2$ [mg/µmol]	3.422/5.75	3.336/5.62
3,3'',4,4''-tetramethoxy-*o*-terphenyl [mg/µmol]	2.044/5.72	1.988/5.67
c [**2**$(PF_6)_2$] [$mol{\cdot}l^{-1}$]	$1.14{\cdot}10^{-2}$	$1.04{\cdot}10^{-2}$
CD_3CN [ml]	0.5	0.5
ratio (GFA/substrate)	1/1	1/1
$HBF_4{\cdot}OEt_2$ (0.12 $g{\cdot}ml^{-1}$ in CD_3CN) [µl/µmol/eq. rel. to **2**$^{2+}$]	1.0/0.73/0.13	10/7.35/1.3
Reaction time (temperature)	300 min (298 K)	10 min (333 K)
conversion to 2,3,10,11-tetramethoxy-triphenylene after workup [%]	88	96

Workup:

The reaction was stopped by the addition of an aqueous saturated $NaHCO_3$ solution. The small amount of CD_3CN was removed under high-vacuum. Then an aqueous diluted solution of NaOH (8%) was added (pH > 9), (converting all formed (**2**+2H)$^{2+}$ to **2**) A clear solution was obtained that was extracted several times with CH_2Cl_2. The combined organic phases were dried over Na_2SO_4, filtrated and condensed.

Conversion vs. time plot for entry B

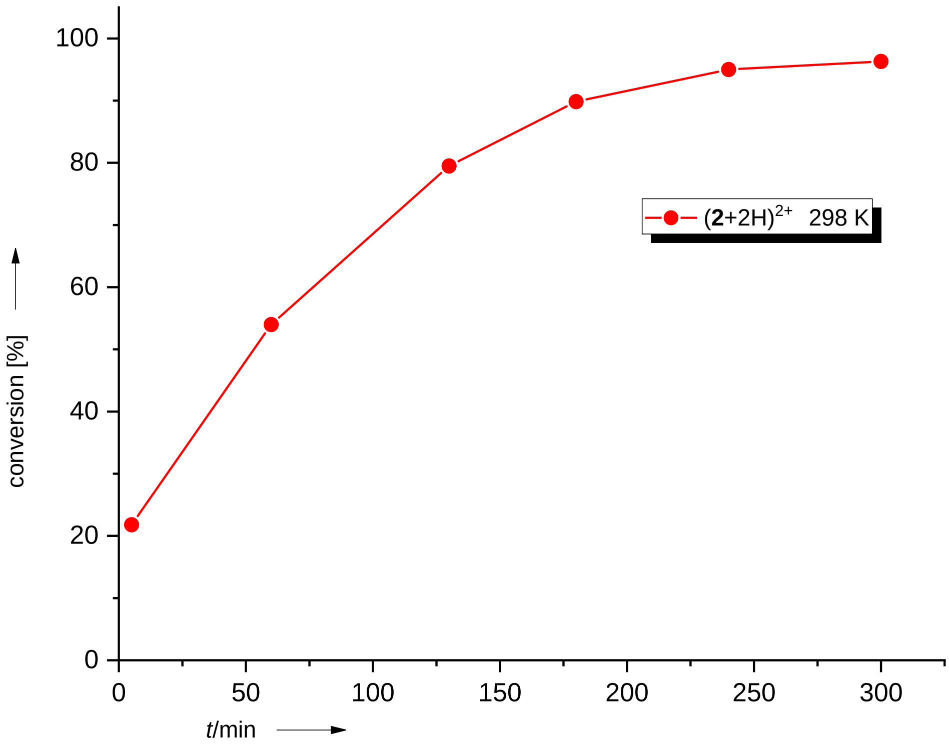

Please note that the conversion in this plot is determined from the integration of ^{1}H NMR signals of **2**$^{2+}$ and (**2**+2H)$^{2+}$. By contrast, the conversion (NMR yield) given in the table was determined after workup. This procedure is necessary due to the emergence of radical intermediates.

^1H NMR spectrum for entry B (199.87 MHz, 298 K, CD_2Cl_2) after workup

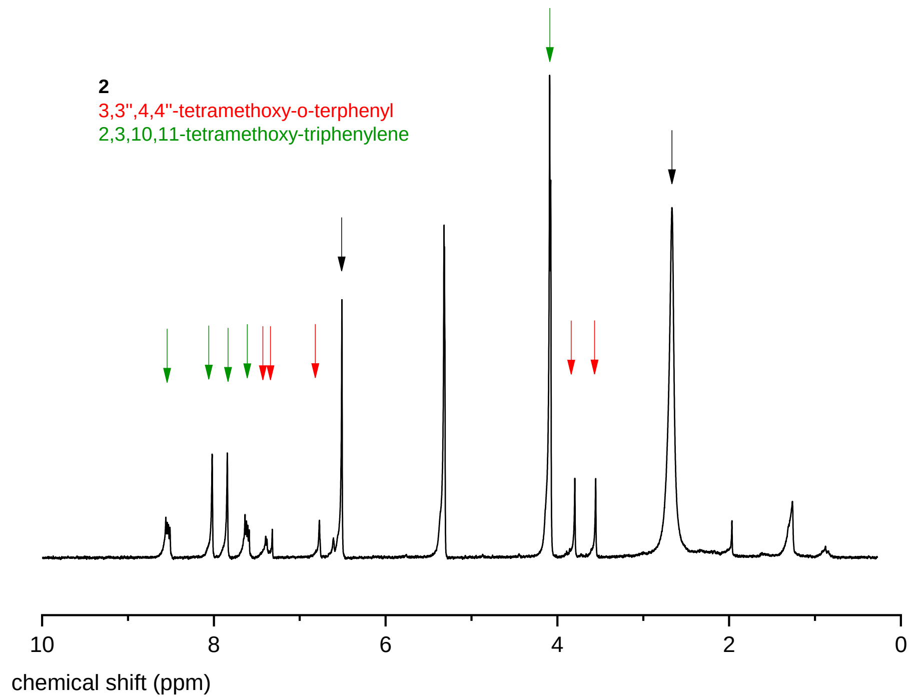

^1H NMR spectrum for entry C (199.87 MHz, 298 K, CD_2Cl_2) after workup

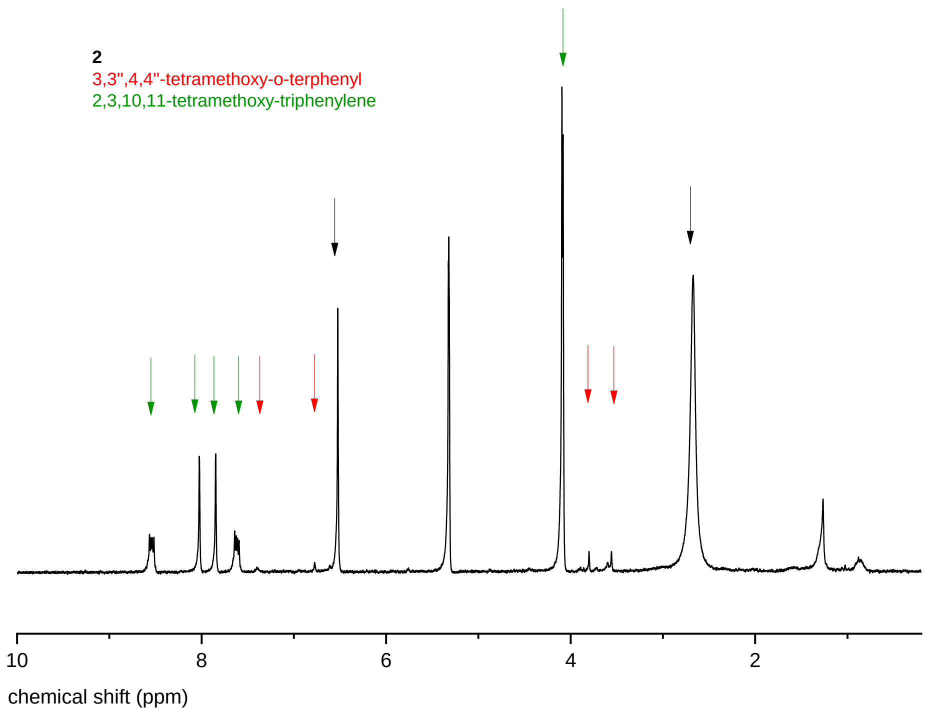

16 Experimental details for reaction of 2(PF_6)$_2$ with *p*-dihydro-benzoquinone to *p*-benzoquinone

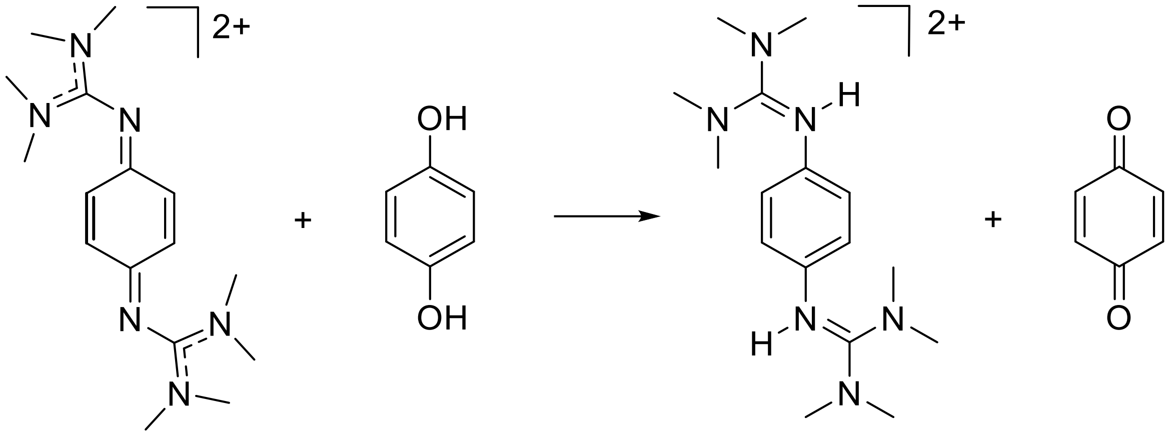

NMR experiments at different concentrations (keeping the 1:1 molar ratio of both reactants)

Protocol: First, two stock solutions were prepared. Stock solution I: 31.774 mg (0.0535 mmol) of **2**(PF_6)$_2$ in 0.85 ml CD_3CN (c = $6.26\cdot10^{-2}$ mol·l^{-1}); stock solution II: 5.932 mg (0.0538 mmol) *p*-dihydro-benzoquinone in 0.86 ml CD_3CN (c = $6.26\cdot10^{-2}$ mol·l^{-1}). Equimolar mixtures were then prepared, in which the concentration of both reactants varied (while keeping a 1:1 molar ratio between them).

entry	A	B	C	D
solution I [ml]	0.25	0.18	0.1	0.05
**2**(PF_6)$_2$ [µmol]	15.7	11.3	6.26	3.13
solution II [ml]	0.25	0.18	0.1	0.05
p-dihydro-benzoquinone [µmol]	15.7	11.3	6.26	3.13
ratio **2**$^{2+}$/*p*-dihydro-benzoquinone	1/1	1/1	1/1	1/1
CD_3CN [ml]	0	0.14	4.8	4.9
c [mol·l^{-1}]	$3.13\cdot10^{-2}$	$2.25\cdot10^{-2}$	$1.25\cdot10^{-2}$	$1.0\cdot10^{-2}$
reaction time (temperature)	60 min (298 K)	60 min (298 K)	30 min (298 K)	15 min (298 K)
conversion [%]	99	99	99	99

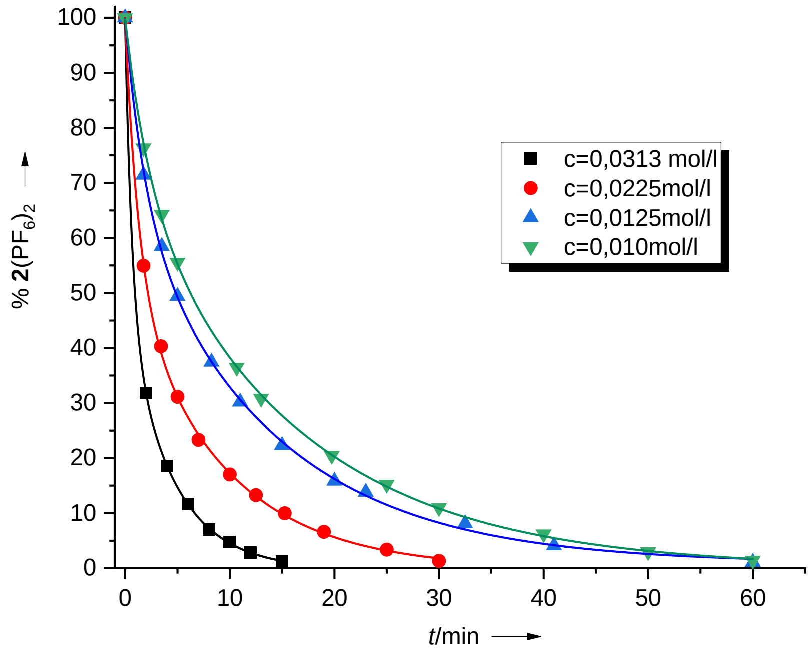

^{1}H NMR spectrum (199.87 MHz, 298 K, CD3CN) for entry D: first measurement after 2.5 min, 298 K

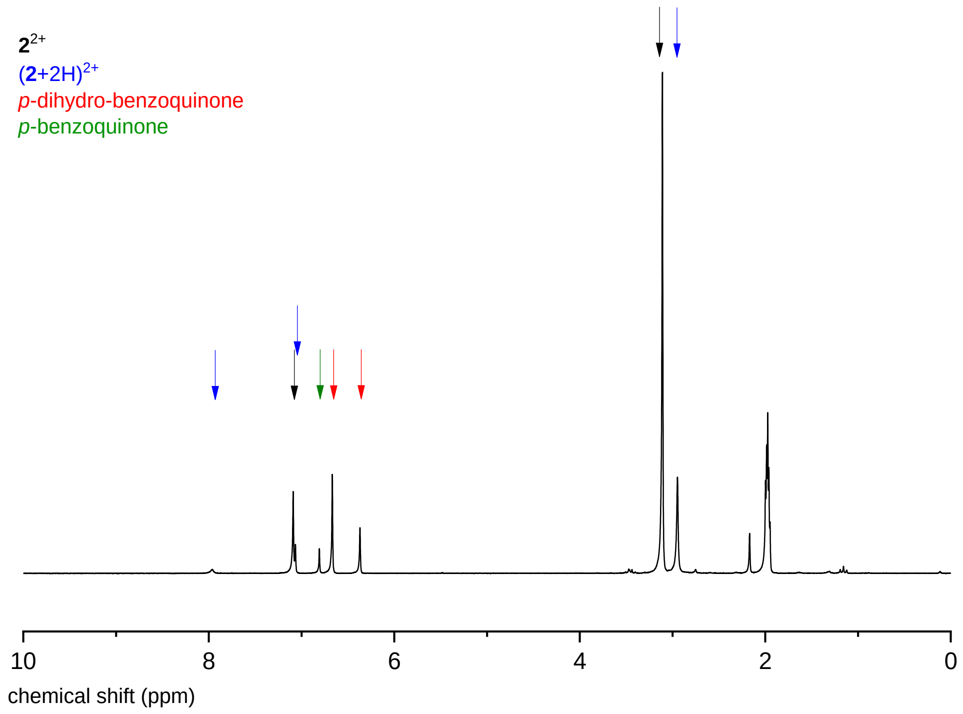

^{1}H NMR spectrum (199.87 MHz, 298 K, CD_3CN) for entry D: last measurement after 60 min, 298 K

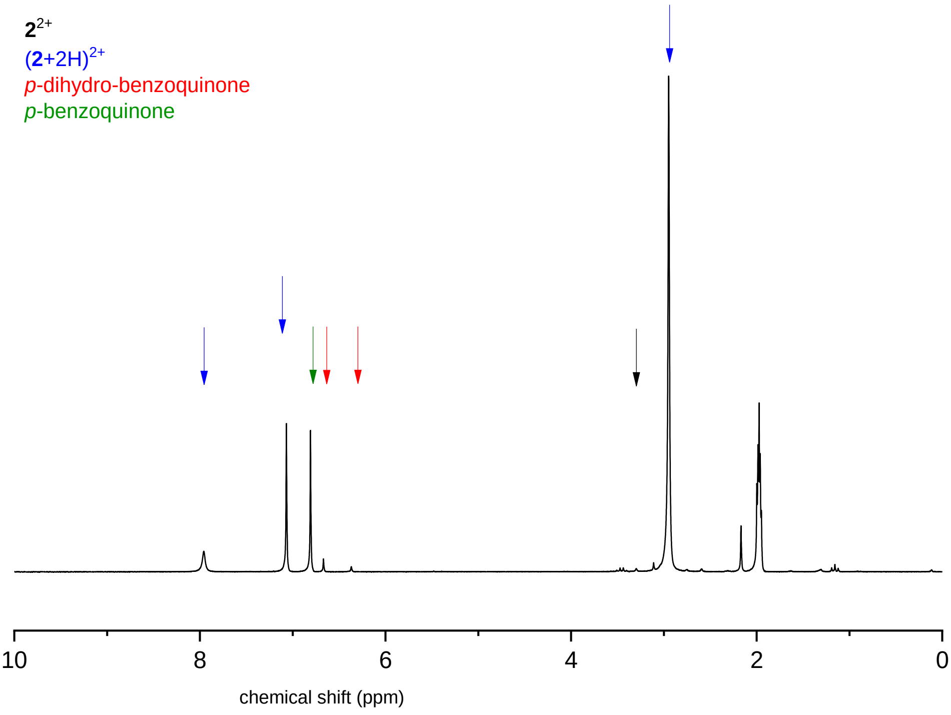

NMR experiments at different temperatures

Protocol: Two stock solutions were prepared. Stock solution I: 22.46 mg (0.038 mmol) of **2**$(PF_6)_2$ in 1.3 ml CD_3CN ($c = 2.9 \cdot 10^{-2}$ mol·l^{-1}); stock solution II: 4.038 mg (0.037 mmol) *p*-dihydro-benzoquinone in 1.26 ml CD_3CN ($c = 2.9 \cdot 10^{-2}$ mol·l^{-1}). Then equimolar mixtures of the two solutions were prepared and the conversion followed by ^{1}H NMR spectroscopy at different reaction temperatures.

**entry**	**E**	**F**	**G**	**H**
**solution I** [ml]	0.25	0.25	0.25	0.25
**2**$(PF_6)_2$ [µmol]	7.3	7.3	7.3	7.3
**solution II** [ml]	0.25	0.25	0.25	0.25
p-dihydro-benzoquinone [µmol]	7.3	7.3	7.3	7.3
ratio **2**$^{2+}$/*p*-dihydro-benzoquinone	1/1	1/1	1/1	1/1
reaction time (temperature)	60 min (298 K)	50 min (308 K)	25 min (318 K)	11.5 min (328 K)
conversion [%]	98	99	>99	>99

^{1}H NMR spectrum (199.87 MHz, 298 K, CD_3CN) for entry E: first measurement after 2.5 min, 298 K

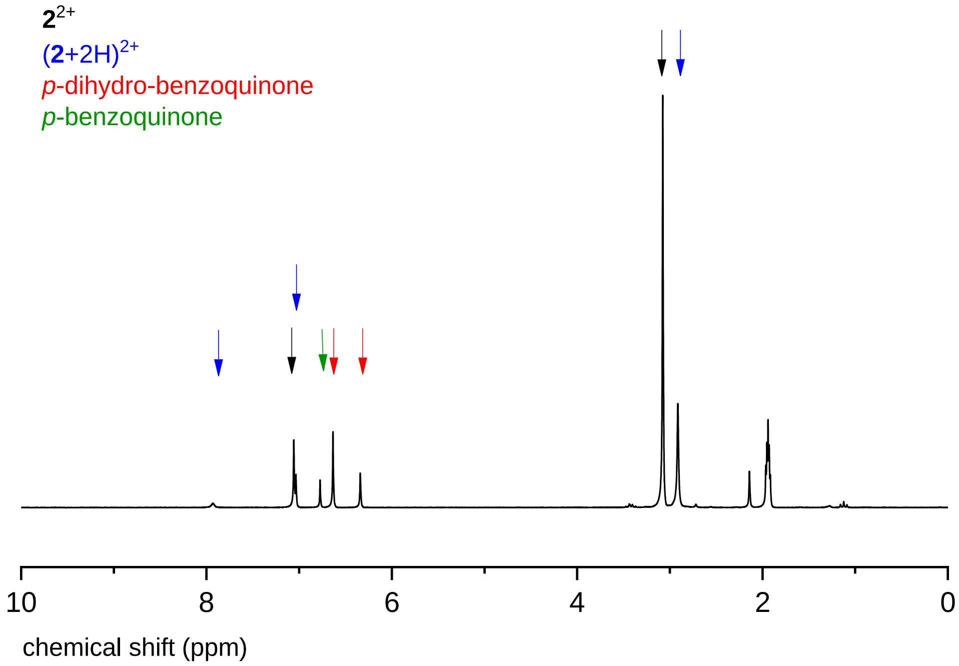

^{1}H NMR spectrum (199.87 MHz, 298 K, CD_3CN) for entry E: last measurement after 60 min, 298 K

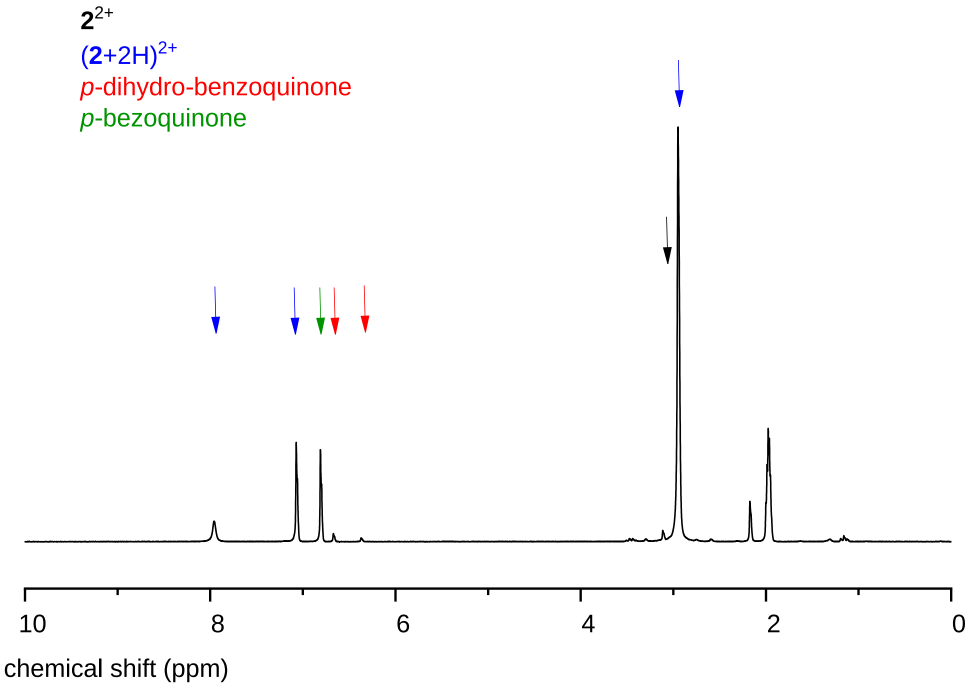

Plot showing the decrease of 2^{2+} (%) with time for different temperatures

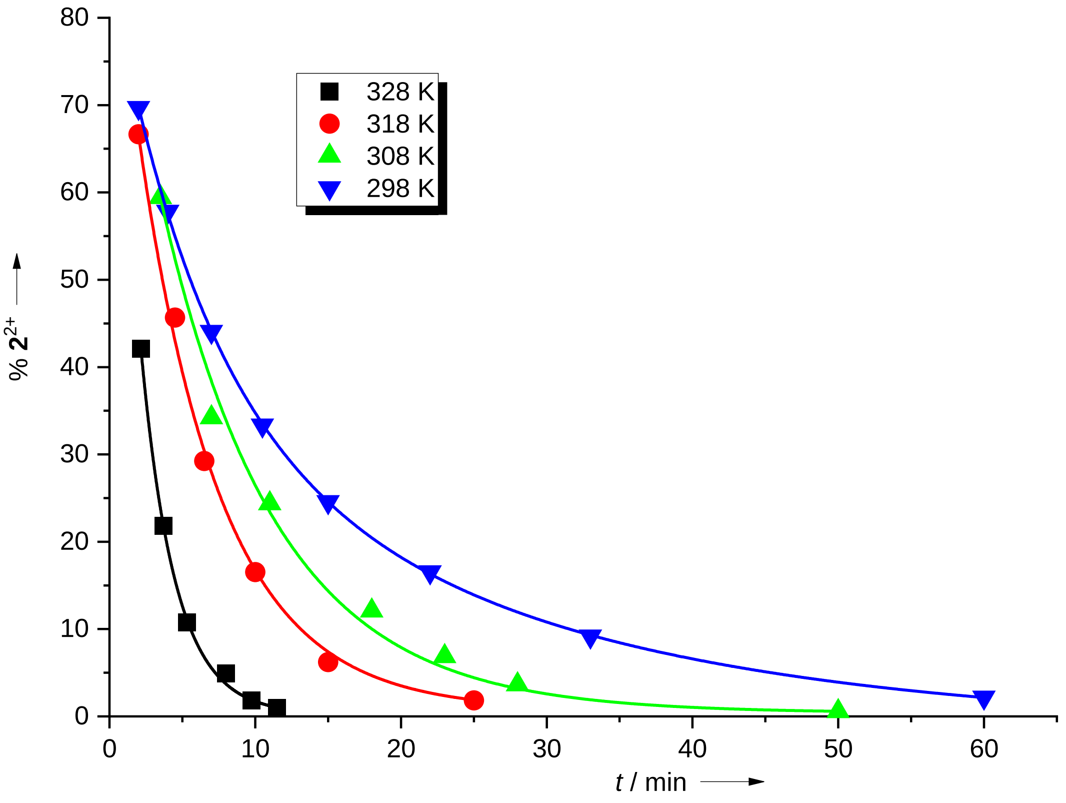

17 Reaction of $2(PF_6)_2$ with $AcrH_2$

UV-vis Experiments

stock solution I: 1.386 mg **2**$(PF_6)_2$ in 25 ml CH_3CN (c = $9.33\cdot10^{-5}$ mol·l^{-1})
stock solution II: 25.2 mg 10-methyl-9,10-dihydroacridine in 5 ml CH_3CN (c = 0.0258 mol·l^{-1})

Under an argon atmosphere a 1 cm quartz cuvette equipped with a rubber septum was filled with 2.8 ml of stock solution I and with CH_3CN if noted below. Stock solution II was added quickly with a syringe. The concentration refers to the concentration in the cuvette (3.2 ml). To check the reproducibility, all experiments are repeated and in all cases similar results are obtained.

**entry**	**stock solution I vol [ml]**	**stock solution II vol [ml]**	**CH_3CN vol [ml]**	**c [$2(PF_6)_2$] [mol·l^{-1}]**	**c [$AcrH_2$] [mol·l^{-1}]**	**eq.**
**A**	2.8	0.1	0.3	$8.16\cdot10^{-5}$	$8.07\cdot10^{-4}$	9.9
**B**	2.8	0.2	0.2	$8.16\cdot10^{-5}$	$1.61\cdot10^{-3}$	19.7
**C**	2.8	0.4	-	$8.16\cdot10^{-5}$	$3.23\cdot10^{-3}$	39.6

UV-vis spectra for entry A:

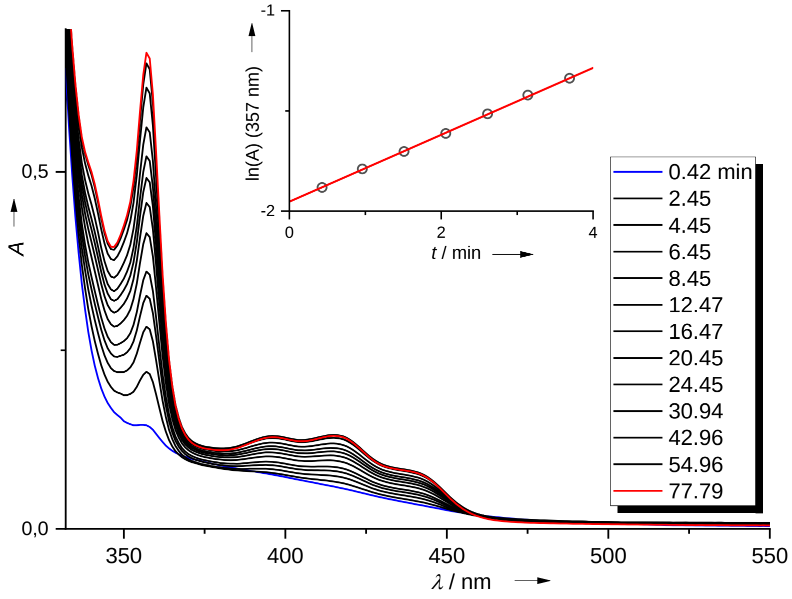

Plots of the absorbance at 357 and 420 nm (maxima of the bands due to $AcrH^+$) and 575 nm (reaction intermediate identified as **2**$^{\cdot+}$) as a function of reaction time

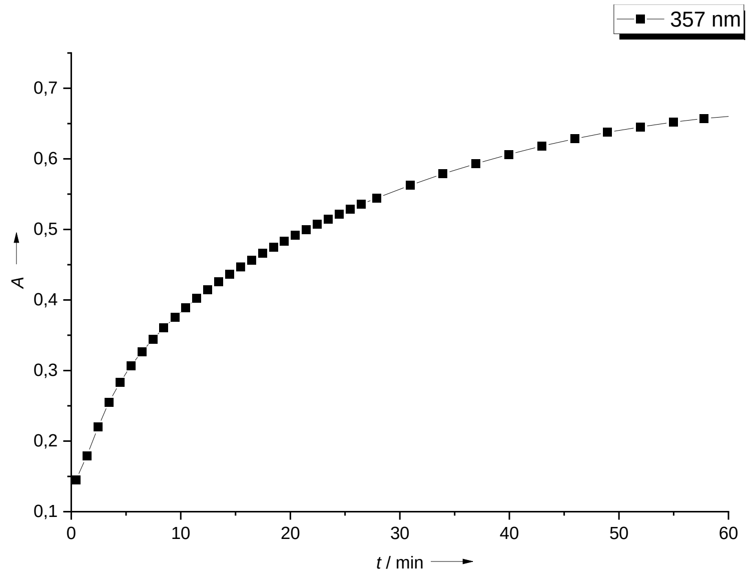

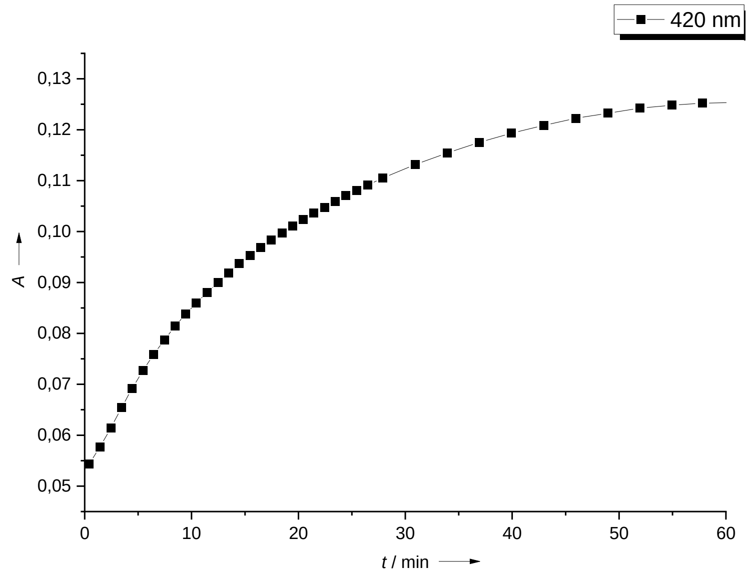

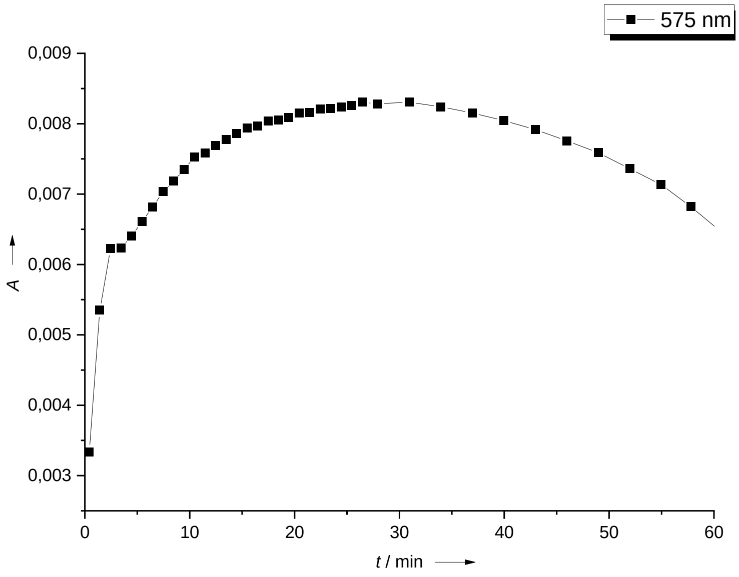

UV-vis spectra for entry B:

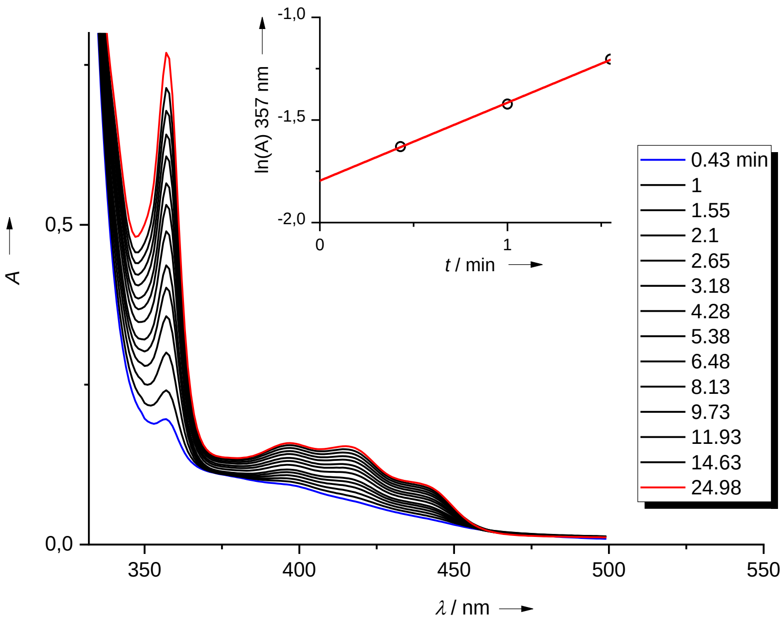

UV-vis spectra for entry C:

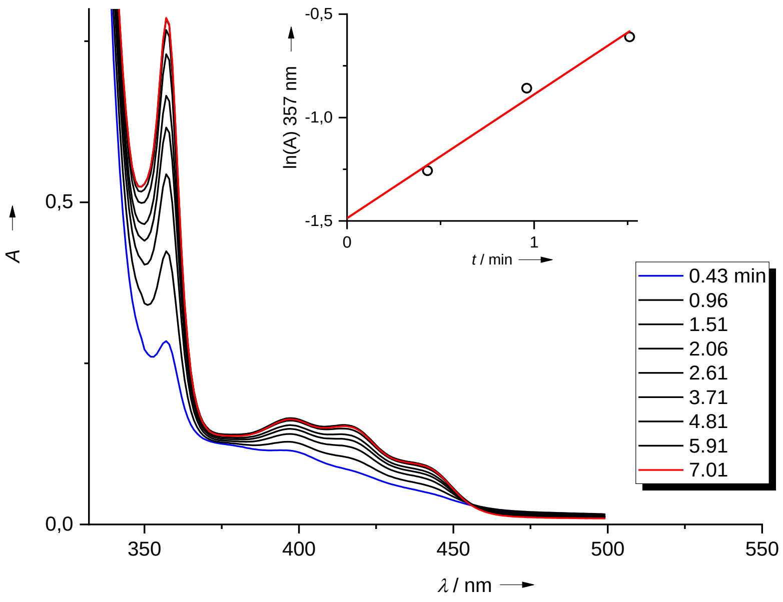

Please note that the bands of $AcrH^+$ are less intense as expected based on the known extinction coefficient ε_{max}[1] = $1.8 \cdot 10^4$ $M^{-1} \cdot cm^{-1}$ for the band at 358 nm.

[1] S. Fukuzumi, K. Okamoto, Y. Tokuda, C. P. Gros, R. Guilard, *J. Am. Chem. Soc.* **2004**, *126*, 17059-17066.

18 Reaction of 2(PF_6)$_2$ with $AcrD_2$

UV-vis Experiments

stock solution I: 1.428 mg **2**(PF_6)$_2$ in 25 ml CH_3CN (c = $9.61 \cdot 10^{-5}$ mol·l^{-1})
stock solution II: 25.6 mg 10-methyl-[9, 9'-$^2H_2$]-acridine in 5 ml CH_3CN (c = 0.0260 mol·l^{-1})

Under an argon atmosphere a 1 cm quartz cuvette equipped with a rubber septum was filled with 2.7 ml of stock solution I and CH_3CN. Stock solution II was added quickly with a syringe. The concentration refers to the concentration in the cuvette (3.2 ml). To check the reproducibility, all experiments are repeated and in all cases similar results are obtained.

**entry**	**stock solution I vol [ml]**	**stock solution II vol [ml]**	**CH_3CN vol [ml]**	**c [2(PF_6)$_2$] [mol·l^{-1}]**	**c [$AcrH_2$] [mol·l^{-1}]**	**eq.**
**A**	2.7	0.1	0.4	$8.11 \cdot 10^{-5}$	$8.11 \cdot 10^{-4}$	10.0
**B**	2.7	0.2	0.3	$8.11 \cdot 10^{-5}$	$1.62 \cdot 10^{-3}$	20.0
**C**	2.7	0.4	0.1	$8.11 \cdot 10^{-5}$	$3.24 \cdot 10^{-3}$	40.0

UV-vis spectra for entry A:

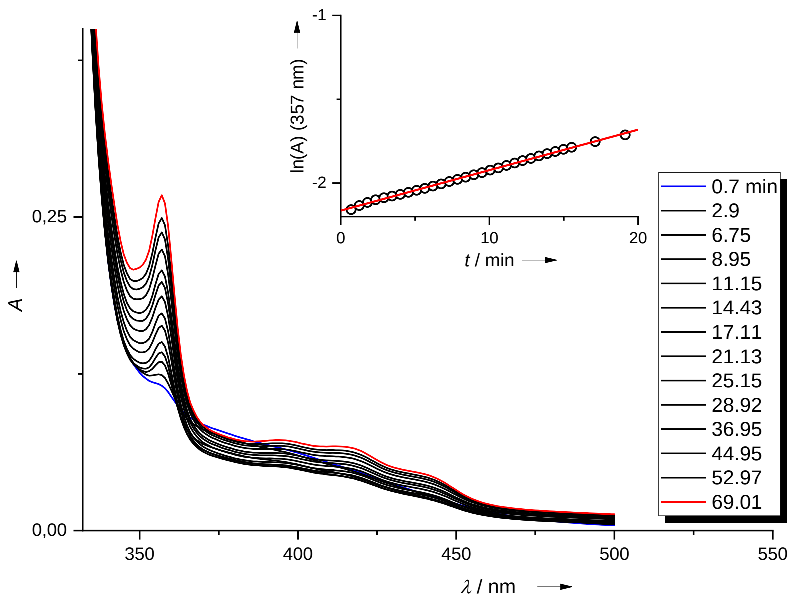

UV-vis spectra for entry B:

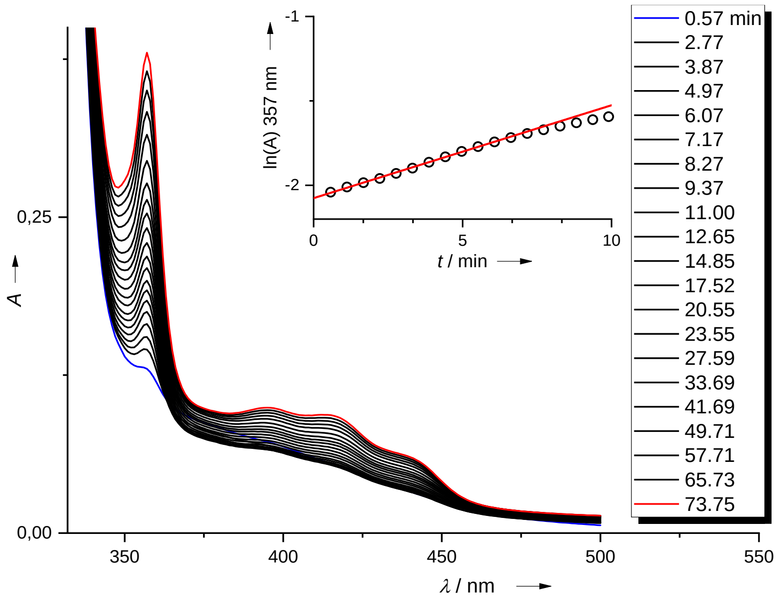

UV-vis spectra for entry C:

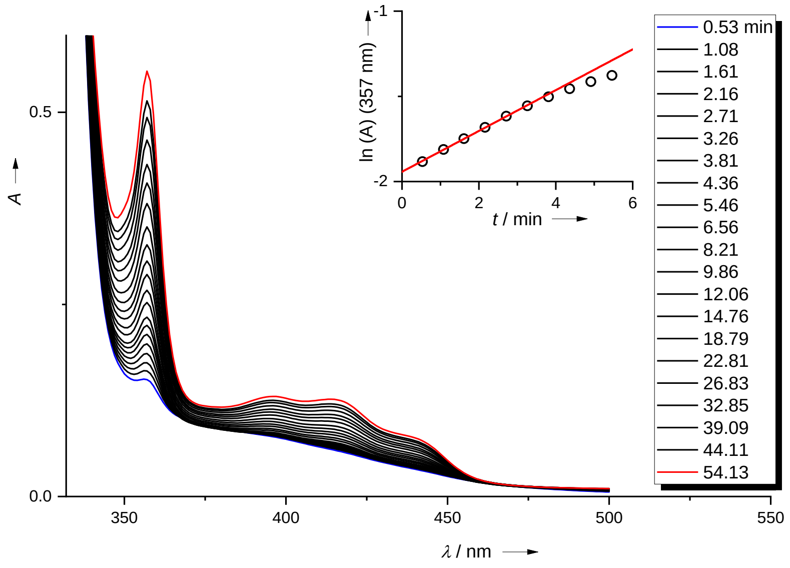

Please note that the bands of $AcrH^+$ are less intense as expected based on the known extinction coefficient ε_{max}[1] = $1.8 \cdot 10^4$ $M^{-1} \cdot cm^{-1}$ for the band at 358 nm.

---

[1] S. Fukuzumi, K. Okamoto, Y. Tokuda, C. P. Gros, R. Guilard, *J. Am. Chem. Soc.* **2004**, *126*, 17059-17066.

19 Experimental details for the PCET reaction between 2(PF_6)$_2$ and (3+2H)(PF_6)$_2$

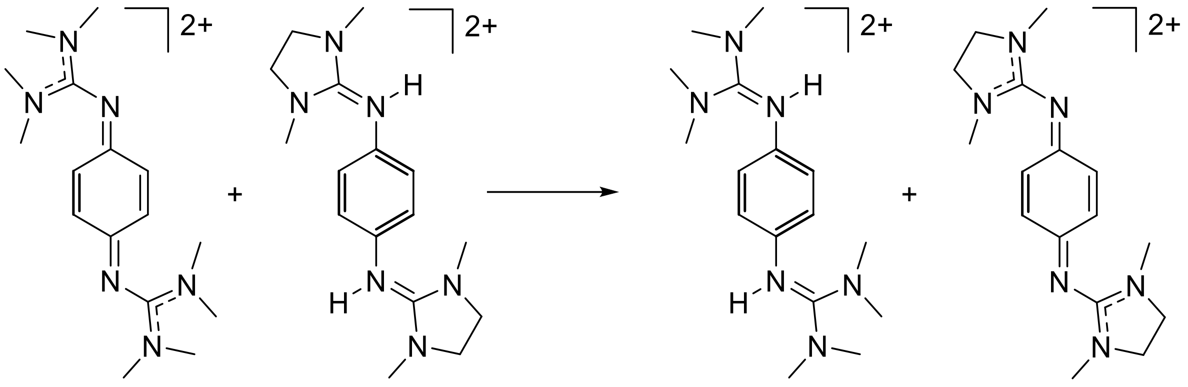

Protocol

First two stock solutions were prepared. Stock solution I: 29.39 mg (0.05 mmol) **2**(PF_6)$_2$ in 1.5 ml CD_3CN (c = $3.33 \cdot 10^{-2}$ mol·l^{-1}); stock solution II: 29.45 mg (0.05 mmol) (**3**+2H)(PF_6)$_2$ in 1.5 ml CD_3CN (c = $3.33 \cdot 10^{-2}$ mol·l^{-1}). Then 0.3 ml of solution I and 0.3 ml of solution II were mixed in an NMR tube and the reaction between **2**(PF_6)$_2$ and (**3**+2H)(PF_6)$_2$ followed at different temperatures by ^{1}H NMR spectroscopy.

**entry**	**A**	**B**	**C**	**D**
solution I [ml]	0.30	0.30	0.30	0.30
**2**(PF_6)$_2$ [µmol]	10	10	10	10
solution II [ml]	0.30	0.30	0.30	0.30
(**3**+2H)(PF_6)$_2$ [µmol]	10	10	10	10
ratio **2**$^{2+}$/(**3**+2H)$^{2+}$	1/1	1/1	1/1	1/1
reaction time (temperature)	180 min (298 K)	95 min (308 K)	60 min (318 K)	45 min (328 K)
ratio (**3**+2H)$^{2+}$/**3**$^{2+}$	0.08/1	0.06/1	0.075/1	0.014/1
conversion to **3**(PF_6)$_2$ [%]	92.6	94.3	93.0	98.6

^{1}H NMR spectrum (199.87 MHz, 298 K, CD_3CN) for entry B: first measurement after 2.5 min, 308 K

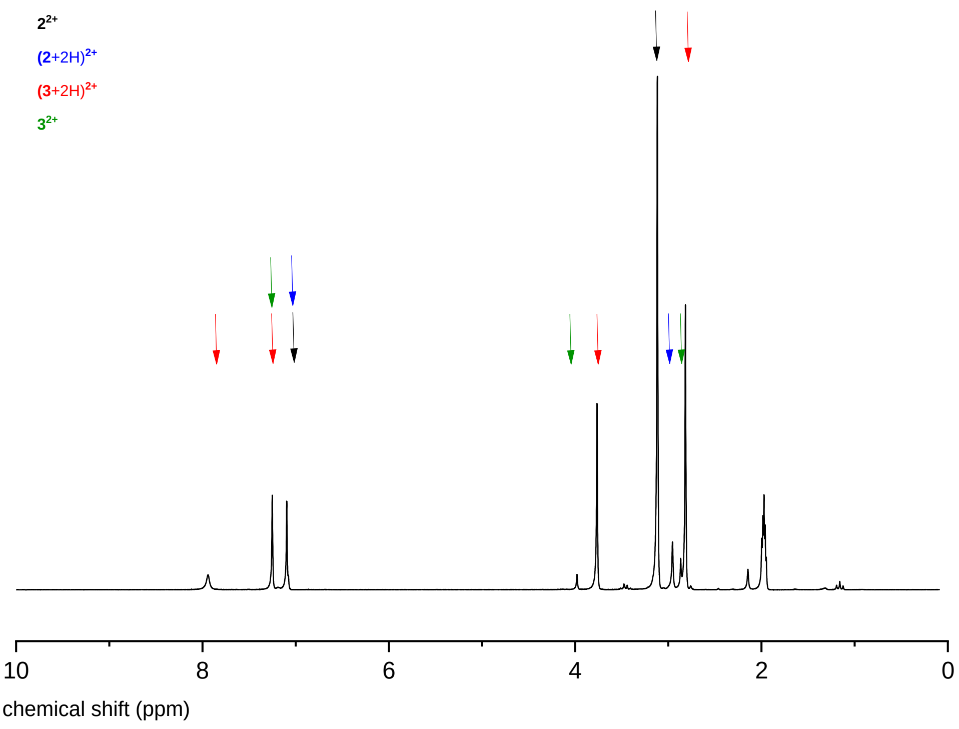

^{1}H NMR spectrum (199.87 MHz, 298 K, CD_3CN) for entry B: last measurement after 90 min, 308 K

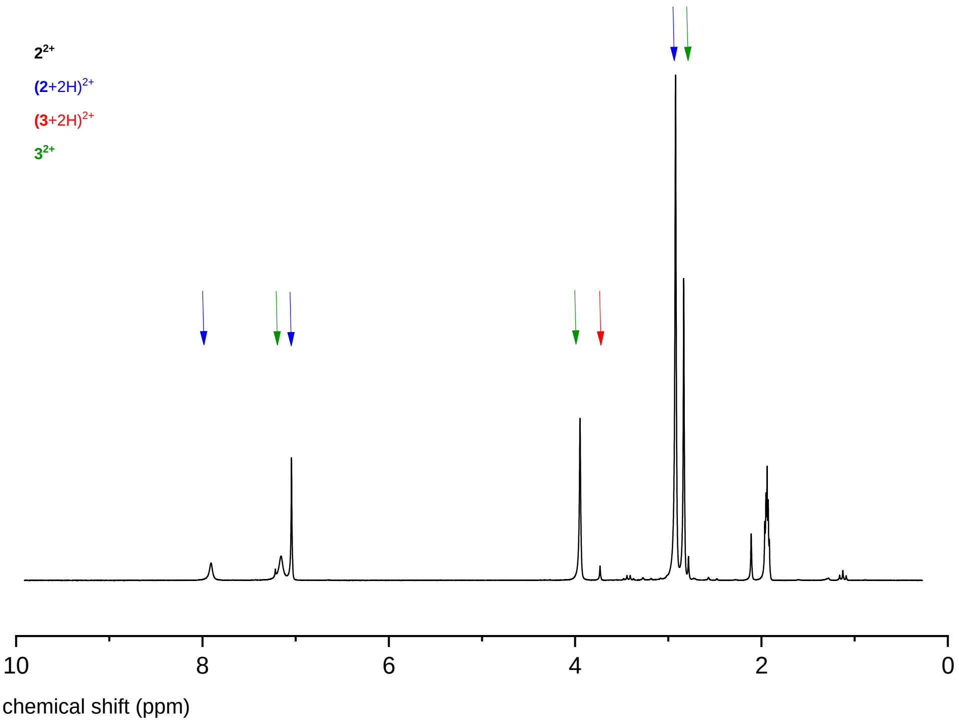

Conversion vs. time plots for four different temperatures

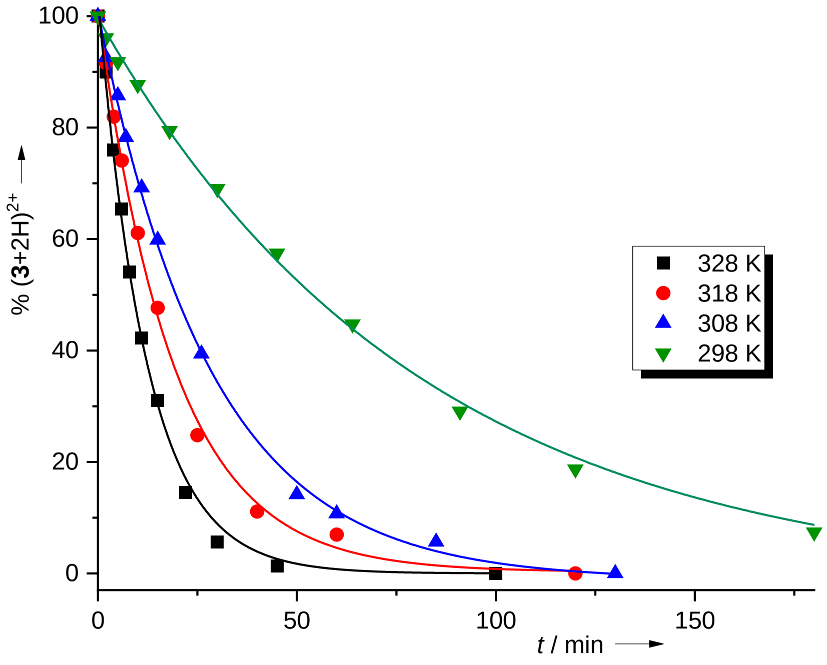

Analysis on the assumption of a second-order rate law:

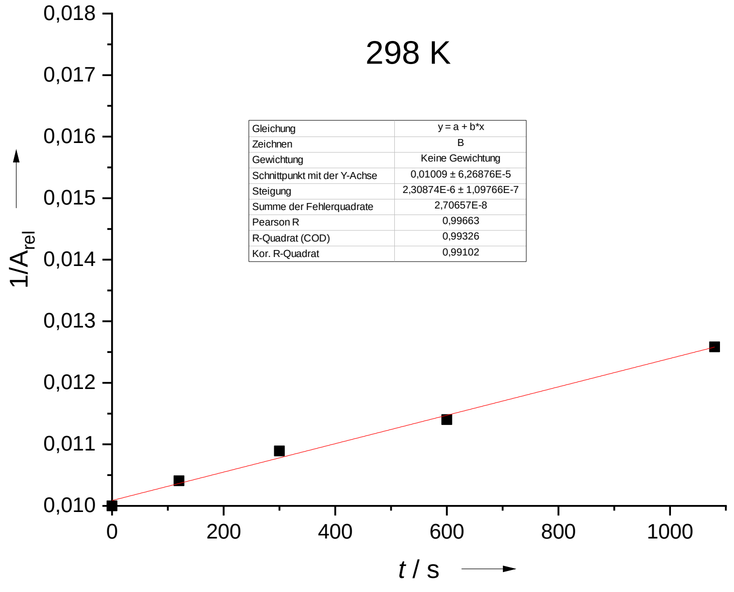

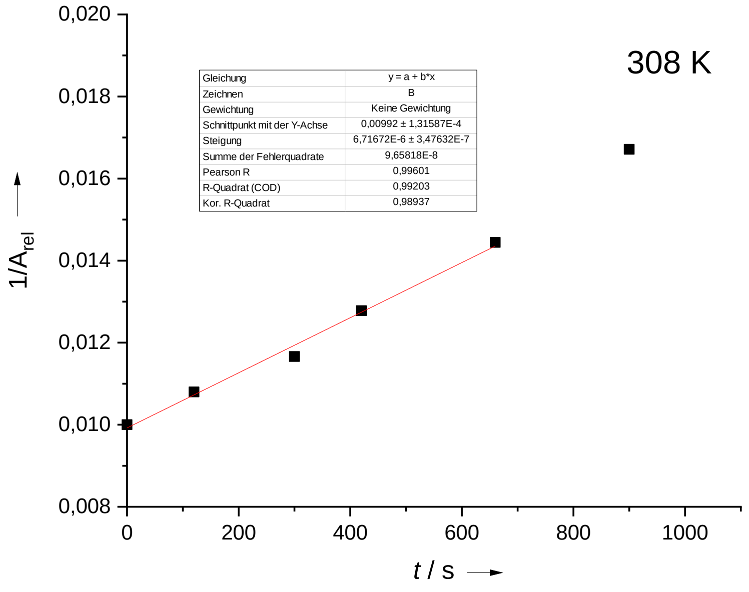
308 K
Gleichung | y = a + b*x
Zeichnen | B
Gewichtung | Keine Gewichtung
Schnittpunkt mit der Y-Achse | 0,00992 ± 1,31587E-4
Steigung | 6,71672E-6 ± 3,47632E-7
Summe der Fehlerquadrate | 9,65818E-8
Pearson R | 0,99601
R-Quadrat (COD) | 0,99203
Kor. R-Quadrat | 0,98937
1/A_rel
t / s

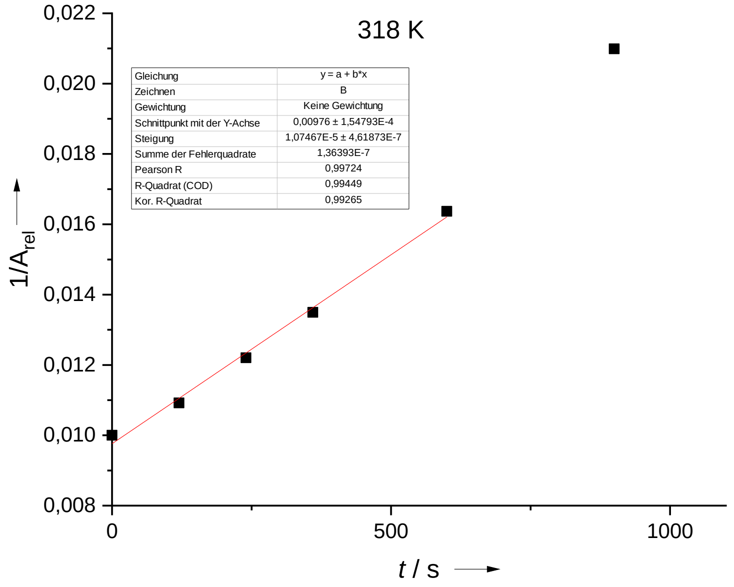
318 K
Gleichung | y = a + b*x
Zeichnen | B
Gewichtung | Keine Gewichtung
Schnittpunkt mit der Y-Achse | 0,00976 ± 1,54793E-4
Steigung | 1,07467E-5 ± 4,61873E-7
Summe der Fehlerquadrate | 1,36393E-7
Pearson R | 0,99724
R-Quadrat (COD) | 0,99449
Kor. R-Quadrat | 0,99265
1/A_rel
t / s

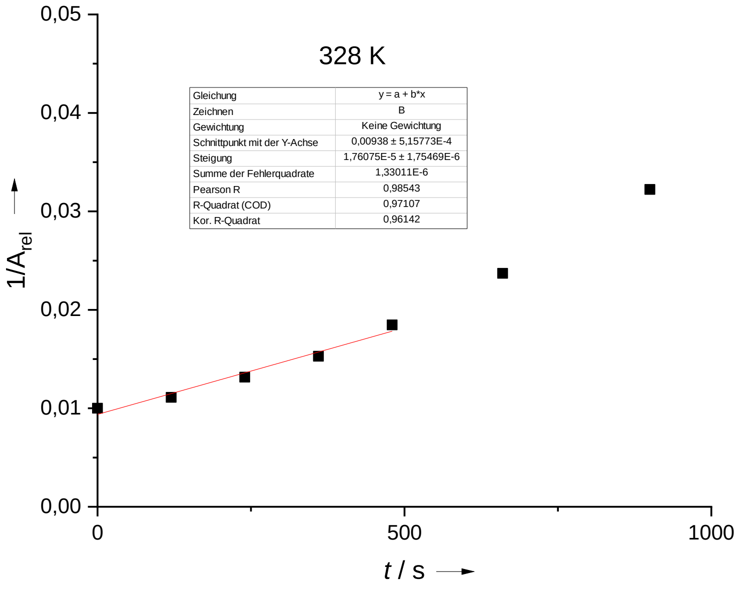

Arrhenius plot to estimate the activation energy from a linear fit

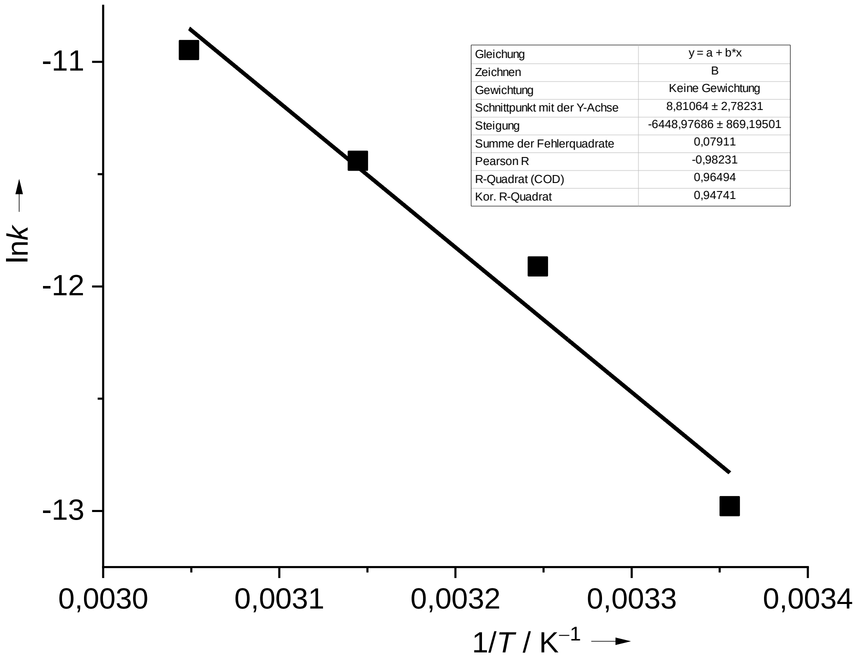

NMR experiments with different ratios of the two reactants

Protocol: First a stock solution of 6.305 mg (1.06 mmol) (**3**+2H)(PF_6)$_2$ in 1.06 ml CD_3CN (c = 1.0·10^{-2} mol·l^{-1}) was prepared (solution I). Compound **2**(PF_6)$_2$ was directly weighed in an NMR tube and dissolved in 0.25 ml CD3CN. Then the stock solution I was added and the conversion followed by ^{1}H NMR spectroscopy.

entry	A	B	C
solution I [ml/µmol]	0.25/2.54	0.25/2.54	0.25/2.54
**2**(PF_6)$_2$ [mg/µmol]	1.512/2.54	14.114/23.75	29.378/49.43
CD_3CN [ml]	0.25	0.25	0.25
ratio **2**$^{2+}$/(**3**+2H)$^{2+}$	1/1	9.5/1	20/1
reaction time (temperature)	90 min (298 K)	5 min (298 K)	2 min (298 K)
conversion to **3**(PF_6)$_2$ [%]	93	95	99

^{1}H NMR spectrum (199.87 MHz, 298 K, CD_3CN) for entry B: first measurement after 1.6 min at 298 K

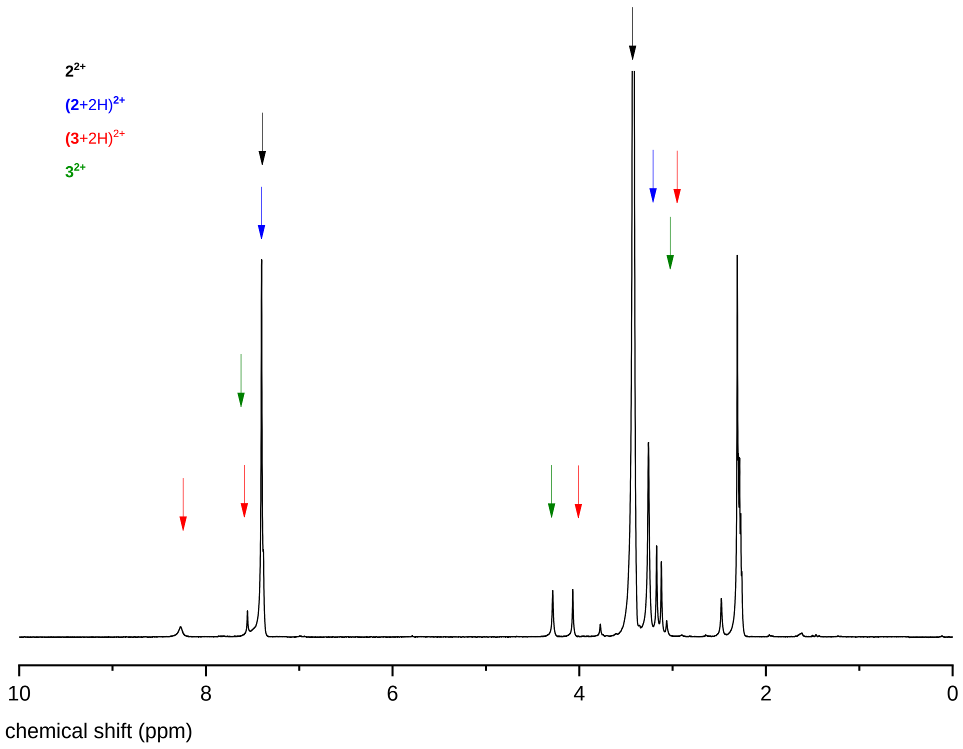

^{1}H NMR spectrum (199.87 MHz, 298 K, CD_3CN) for entry B: last measurement after 5 min at 298 K

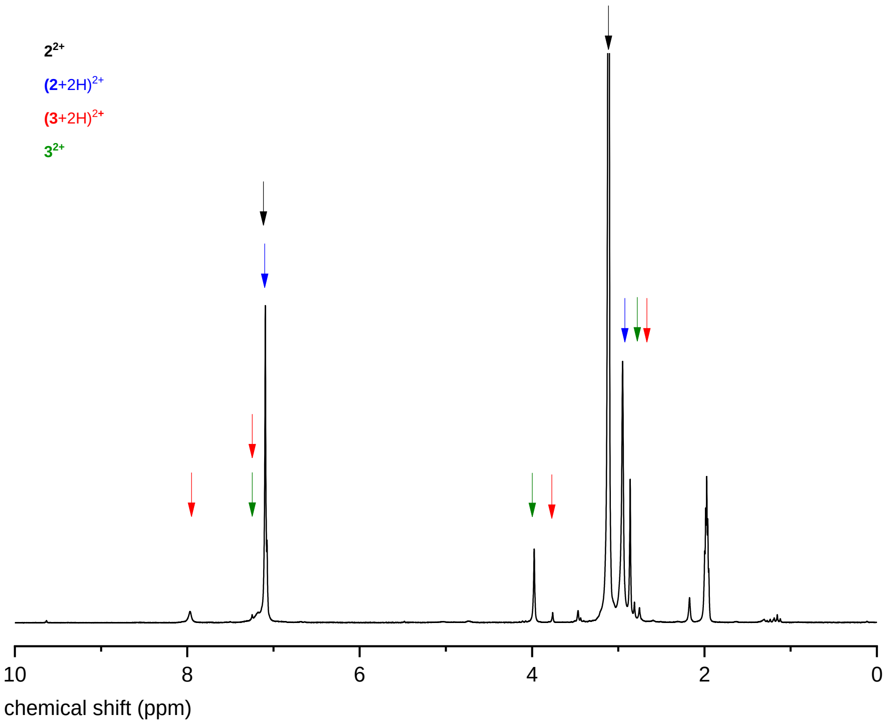

Plot showing the decrease of (**3**+2H)$(PF_6)_2$ (%) with time

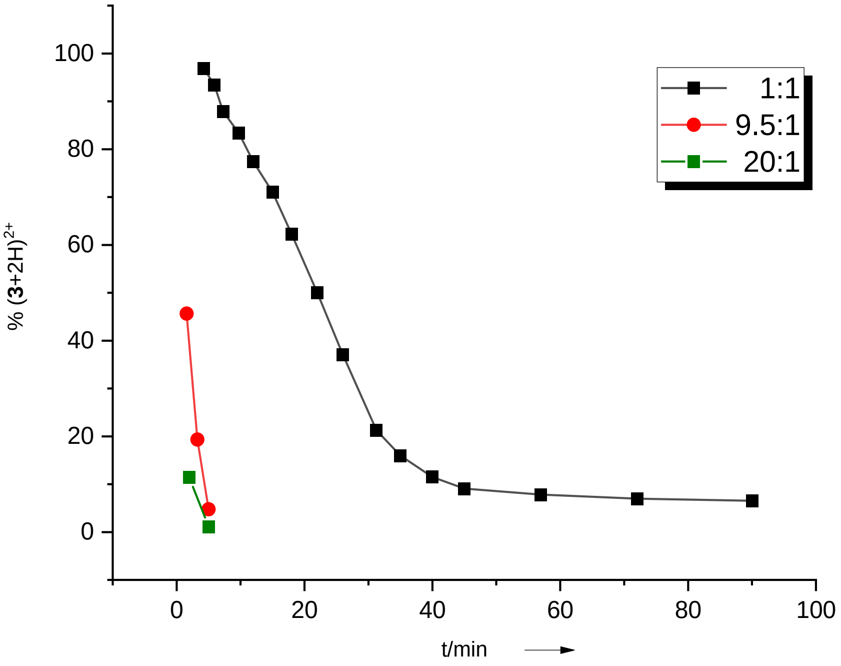

UV-vis experiments

1) Titration of **3** with **3**$(PF_6)_2$

	solution I	solution II
	**3**	**3**$(PF_6)_2$
[mg/mmol]	0.116/3.86·10^{-4}	3.176/5.38·10^{-3}
CH_3CN [ml]	10	0.466
c [mol l^{-1}]	3.86·10^{-5}	1.15·10^{-2}

Protocol: A 1 cm quartz cuvette equipped with a silicon septum was filled with 3.0 ml of stock solution I (1.15·10^{-4} mmol). Then portions of 5 µl of stock solution II (5.75·10^{-5} mmol/0.5 eq.) were added, the mixture stirred for 1 min, and an UV-vis spectrum recorded.

UV-vis spectra

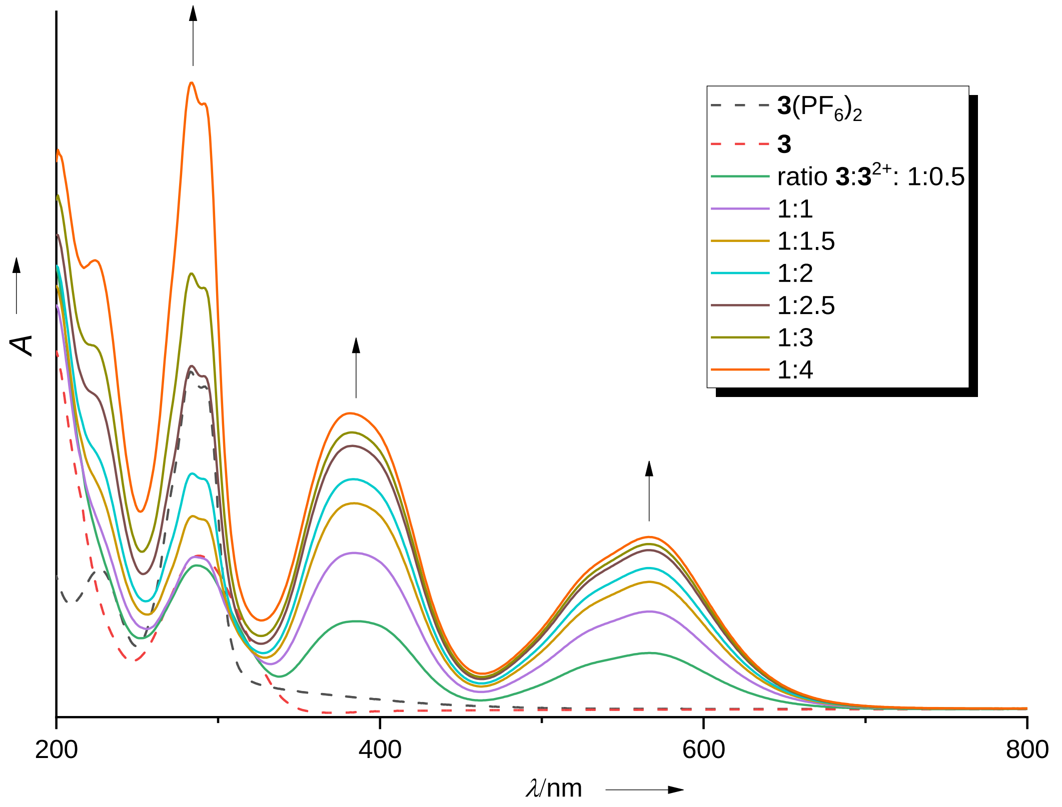

2) Degradation of a mixture of **2** + **2**($(PF_6)_2$)

	solution I	solution II
	**2**	**2**$(PF_6)_2$
[mg/mmol]	0.282/9.25·10^{-4}	0.463/7.8·10^{-4}
CH_3CN [ml]	10	10
c [mol·l^{-1}]	9.26·10^{-5}	7.8·10^{-5}

Protocol: 1.25 ml of solution I (1.16·10^{-4} mmol), 0.2 ml CH_3CN and 1.5 ml solution II (1.17·10^{-4} mmol) were mixed under stirring in a 1 cm quartz cuvette equipped with a silicon septum. UV-vis spectra were taken after constant time intervals.

UV-vis spectra

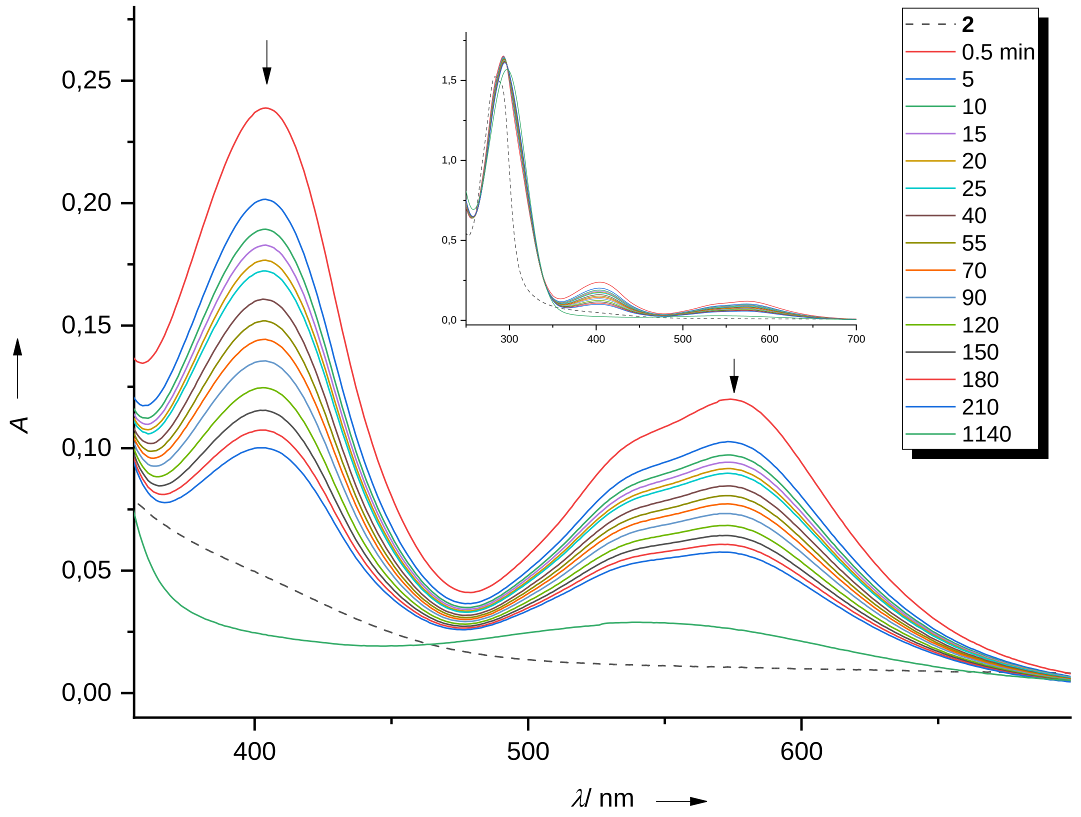

3) Degradation of a mixture of **3** + **3**(PF_6)$_2$

	solution I	solution II
	**3**	**3**(PF_6)$_2$
[mg/mmol]	0.116/3.86·10^{-4}	3.176/5.38·10^{-3}
CH_3CN [ml]	10	0.466
c [mol·l^{-1}]	3.86·10^{-5}	1.15·10^{-2}

Protocol: Under stirring a 1 cm quartz cuvette equipped with a silicon septum was filled with 3.0 ml of solution I (1.16·10^{-4} mmol), and 10 µl solution II (1.15·10^{-4} mmol). UV-vis spectra were taken after constant time intervals.

UV-vis spectra

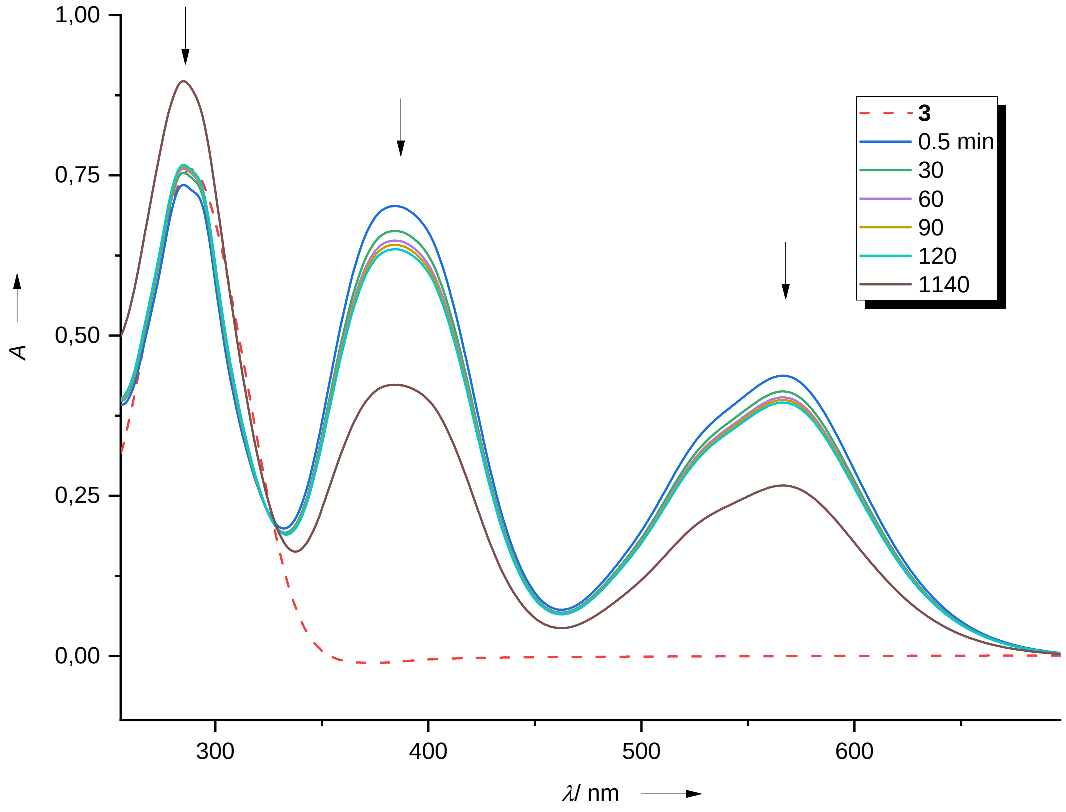

20 Details of the recycling of 1(PF_6)$_2$

1. Separation from the reaction product

Separation of (**1**+2H)X_2 and traces of unreacted (**1**)X_2 (X^- = ClO_4^-, BF_4^- or PF_6^-) from the reaction mixtures was carried out for the oxidative coupling of *N*-ethylcarbazole to *N,N'*-diethyl-3,3'-bicarbazole (see Scheme 1 in the paper), oxidative coupling of 3,3''-dimethoxy-3',4'-dimethyl-*o*-terphenyl to 3,10-dimethoxy-6,7-dimethyltriphenylene (Scheme 2 in the paper) and reaction of **1**(PF_6)$_2$ with 9,10-dihydroanthracene (AnH_2, Scheme 4 in the paper). In all cases the residue, containing the reaction products, was collected in diethylether and filtrated, allowing the isolation of the coupling product or anthracene in the filtrate. The residue in the filter, consisting of (**1**+2H)X_2 (X^- = ClO_4^-, BF_4^- or PF_6^-) together with traces of unreacted PCET reagent (**1**)X_2, was dissolved in CH_3CN for catalytic re-oxidation to the guanidine PCET reagent (**1**)X_2.

2. Catalytic re-oxidation to give the PCET reagent

In an O_2 atmosphere, the salt (**1**+2H)X_2 (X^- = PF_6^-) and the dioxygen-activating catalyst (3 mol% of a 1:1 mixture of $CuCl_2$ and $Cu(H_2O)_6(BF_4)_2$) were dissolved in acetonitrile. Quantitative conversion of $(\mathbf{1}+2H)^{2+}$ into $\mathbf{1}^{2+}$ according to the reaction equation given below is obtained at room temperature in 22 min.

$$(\mathbf{1}+2H)X_2 \xrightarrow[\text{CH}_3\text{CN, r.t., 22 min}]{\text{O}_2,\ 3\text{ mol\% }[\text{CuCl}_2/\text{Cu(H}_2\text{O)}_6(\text{BF}_4)_2]} (\mathbf{1})X_2$$

>99% yield

For further details (e.g. catalyst screening), see: U. Wild, F. Schön, H.-J. Himmel, *Angew. Chem.* **2017**, *129*, 16630–16633; *Angew. Chem. Int. Ed.* **2017**, *56*, 16410–16413.

21 Details of the quantum chemical calculations

Density functional calculations are performed with the program TURBOMOLE.[1-3] The B3LYP functional [4,5] is used in connection with the def2-SV(P) and def2-TZVP basis sets.[6] For the calculation of the two-electron integrals, the resolution-of-the-identity (RI) approximation [7] is used with the appropriate def2-SV(P) and def2-TZVP auxiliary basis set.[8] Structure optimizations are performed with the def2-SV(P) and def2-TZVP basis sets, the determination of the harmonic vibrational frequencies with the def2-SV(P) basis set only. The electronic excitation energies are obtained by time-dependent density functional calculations.[9,10]

[1] TURBOMOLE V7.2 2017, a development of University of Karlsruhe and Forschungszentrum Karlsruhe GmbH, 1989-2007, TURBOMOLE GmbH, since 2007; available from http://www.turbomole.com.

[2] R. Ahlrichs, M. Bär, M. Häser, H. Horn, C. Kölmel, *Chem. Phys. Lett.* **1989**, *162*, 165–169.

[3] O. Treutler, R. Ahlrichs, *J. Chem. Phys.* **1995**, *102*, 346–354.

[4] A. D. Becke, *J. Chem. Phys.* **1993**, *98*, 5648–5652.

[5] P. J. Stephens, F. J. Devlin, C. F. Chabalowski, M. J. Frisch, *J. Phys. Chem.* **1994**, *98*, 11623–11627.

[6] F. Weigend, R. Ahlrichs, *Phys. Chem. Chem. Phys.* **2005**, *7*, 3297–3305.

[7] K. Eichkorn, O. Treutler, H. Öhm, M. Häser, R. Ahlrichs, *Chem. Phys. Lett.* **1995**, *242*, 652–660.

[8] F. Weigend, *Phys. Chem. Chem. Phys.* **2006**, *8*, 1057–1065.

[9] R. Bauernschmitt, R. Ahlrichs, *Chem. Phys. Lett.* **1996**, *256*, 454–464.

[10] R. Bauernschmitt, M. Häser, O. Treuler, R. Ahlrichs, *Chem. Phys. Lett.* **1997**, *264*, 573–578.

Thermodynamics of the reactions

Reactions with 9,10-dihydroanthracene

a) Without inclusion of anions

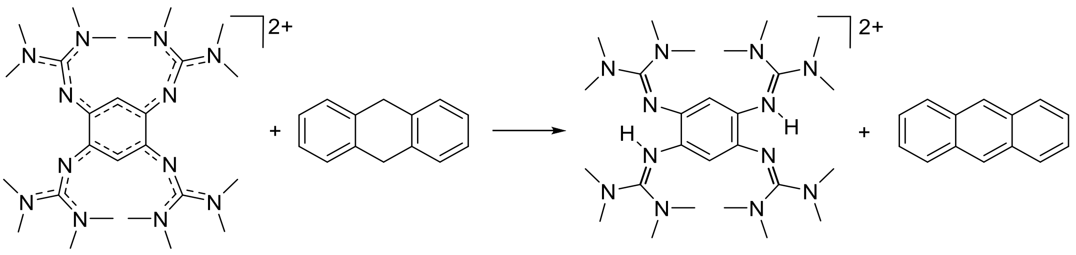

	SV(P)	TZVP[a] (ε_r = 1)
$\Delta_r E$ / kJ mol^{-1}	-94.0	-96.0
$\Delta_r H$ (0 K) / kJ mol^{-1}	-90.2	-92.1
$\Delta_r G$ (298 K) / kJ mol^{-1}	-84.5	-86.5

[a] Thermodynamic contributions with the SV(P) basis set.

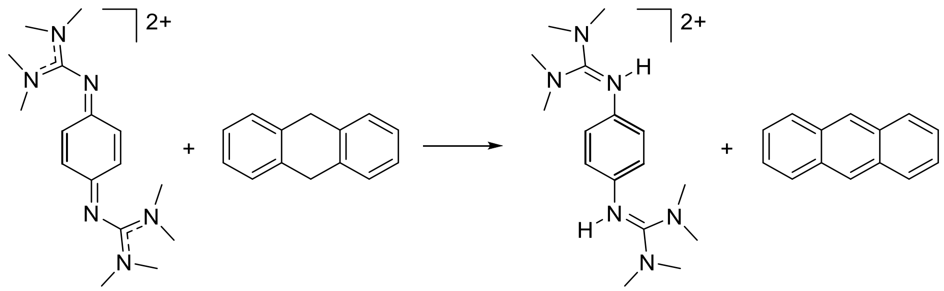

	SV(P)	TZVP[a] (ε_r = 1)
$\Delta_r E$ / kJ mol^{-1}	-192.2	-187.8
$\Delta_r H$ (0 K) / kJ mol^{-1}	-184.2	-179.8
$\Delta_r G$ (298 K) / kJ mol^{-1}	-176.3	-171.9

[a] Thermodynamic contributions with the SV(P) basis set.

b) With inclusion of anions and solvent-effect (COSMO)

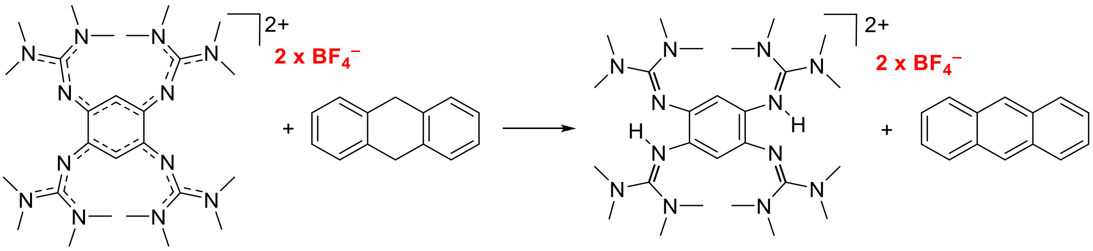

	SV(P)	TZVP[a] (ε_r = 1)	TZVP[a] (ε_r = 37.5)
$\Delta_r E$ / kJ mol^{-1}	-89.3	-90.3	-107.1
$\Delta_r H$ (0 K) / kJ mol^{-1}	-90.5	-91.6	-108.3
$\Delta_r G$ (298 K) / kJ mol^{-1}	-86.7	-87.7	-104.5

[a] Thermodynamic contributions with the SV(P) basis set.

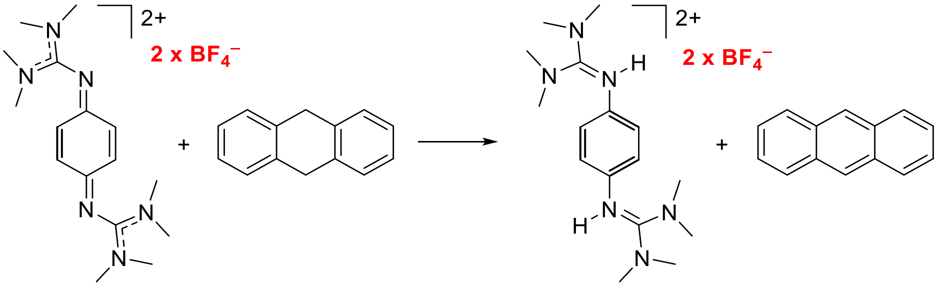

	SV(P)	TZVP[a] (ε_r = 1)	TZVP[a] (ε_r = 37.5)
$\Delta_r E$ / kJ mol^{-1}	-216.8	-207.9	-203.5
$\Delta_r H$ (0 K) / kJ mol^{-1}	-210.9	-202.0	-197.6
$\Delta_r G$ (298 K) / kJ mol^{-1}	-202.3	-193.4	-189.0

[a] Thermodynamic contributions with the SV(P) basis set.

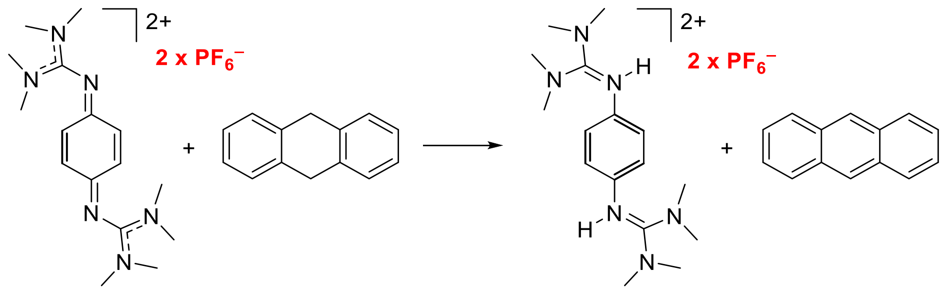

	SV(P)	TZVP[a] (ε_r = 1)
$\Delta_r E$ / kJ mol^{-1}	-202.7	-204.2
$\Delta_r H$ (0 K) / kJ mol^{-1}	-193.0	-194.5
$\Delta_r G$ (298 K) / kJ mol^{-1}	-182.8	-184.3

[a] Thermodynamic contributions with the SV(P) basis set.

Reactions with *p*-dihydrobenzoquinone

a) Without inclusion of anions

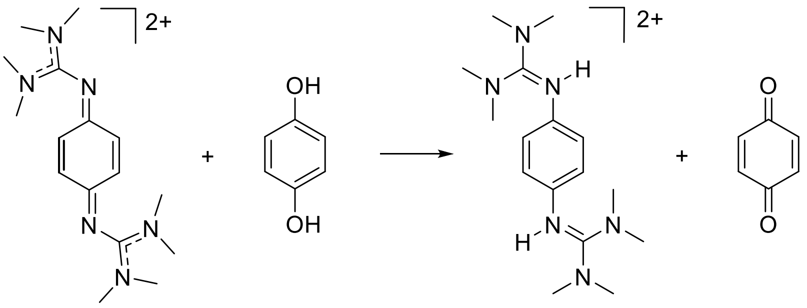

	SV(P)	TZVP[a] (ε_r = 1)
$\Delta_r E$ / kJ mol^{-1}	-137.6	-98.1
$\Delta_r H$ (0 K) / kJ mol^{-1}	-131.1	-91.6
$\Delta_r G$ (298 K) / kJ mol^{-1}	-125.9	-86.4

[a] Thermodynamic contributions with the SV(P) basis set.

b) With inclusion of anions and solvent-effect (COSMO)

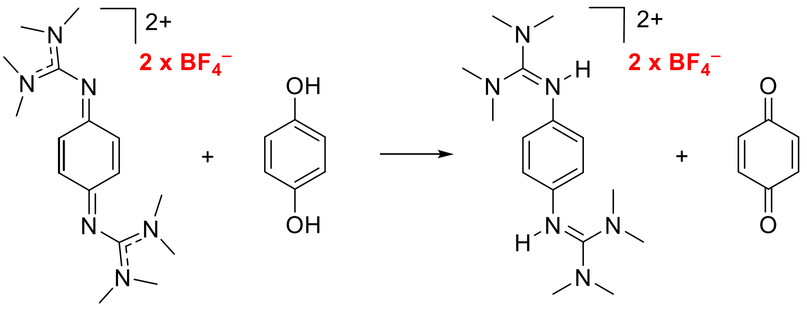

	SV(P)	TZVP[a] (ε_r = 1)	TZVP[a] (ε_r = 37.5)
$\Delta_r E$ / kJ mol^{-1}	-162.2	-118.2	-106.5
$\Delta_r H$ (0 K) / kJ mol^{-1}	-157.8	-113.7	-102.0
$\Delta_r G$ (298 K) / kJ mol^{-1}	-151.9	-107.8	-96.1

[a] Thermodynamic contributions with the SV(P) basis set.

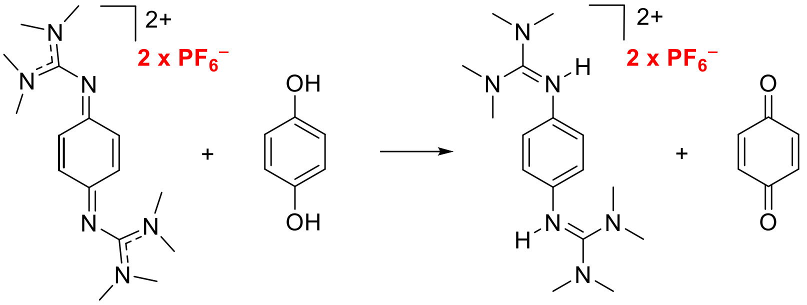

	SV(P)	TZVP[a] (ε_r = 1)
$\Delta_r E$ / kJ mol^{-1}	-148.1	-114.5
$\Delta_r H$ (0 K) / kJ mol^{-1}	-139.9	-106.3
$\Delta_r G$ (298 K) / kJ mol^{-1}	-132.3	-98.7

[a] Thermodynamic contributions with the SV(P) basis set.

PCET reaction between 2^{2+} and $(1{+}2H)^{2+}$

a) Without inclusion of anions

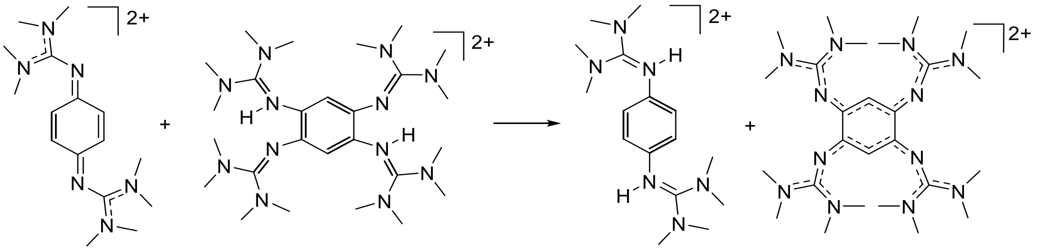

	SV(P)	TZVP[a] (ε_r = 1)
$\Delta_r E$ / kJ mol^{-1}	-98.2	-91.9
$\Delta_r H$ (0 K) / kJ mol^{-1}	-94.0	-87.6
$\Delta_r G$ (298 K) / kJ mol^{-1}	-91.8	-85.4

[a] Thermodynamic contributions with the SV(P) basis set.

b) With inclusion of anions and solvent-effect (COSMO)

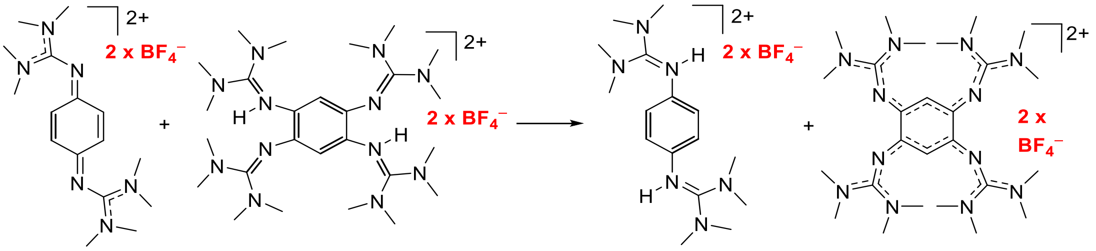

	SV(P)	TZVP[a] (ε_r = 1)	TZVP[a] (ε_r = 37.5)
$\Delta_r E$ / kJ mol^{-1}	-127.5	-117.6	-96.4
$\Delta_r H$ (0 K) / kJ mol^{-1}	-120.3	-110.4	-89.2
$\Delta_r G$ (298 K) / kJ mol^{-1}	-115.6	-105.7	-84.5

[a] Thermodynamic contributions with the SV(P) basis set.

PCET reaction between 2^{2+} and $(3+2H)^{2+}$

a) Without inclusion of anions

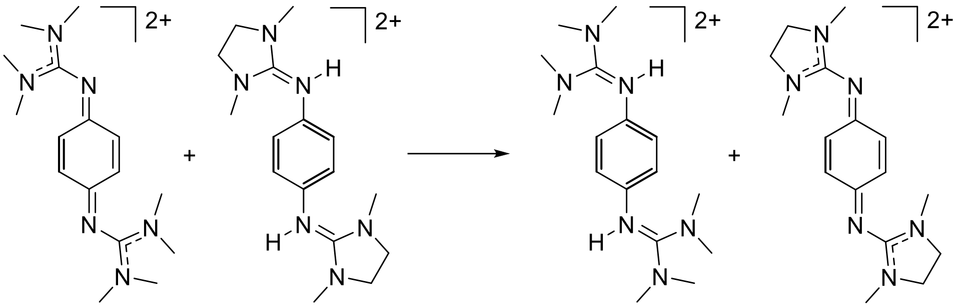

	SV(P)	TZVP[a] (ε_r = 1)
$\Delta_r E$ / kJ mol^{-1}	42.5	-40.7
$\Delta_r H$ (0 K) / kJ mol^{-1}	41.8	-39.9
$\Delta_r G$ (298 K) / kJ mol^{-1}	42.0	-40.2

[a] Thermodynamic contributions with the SV(P) basis set.

b) With inclusion of anions

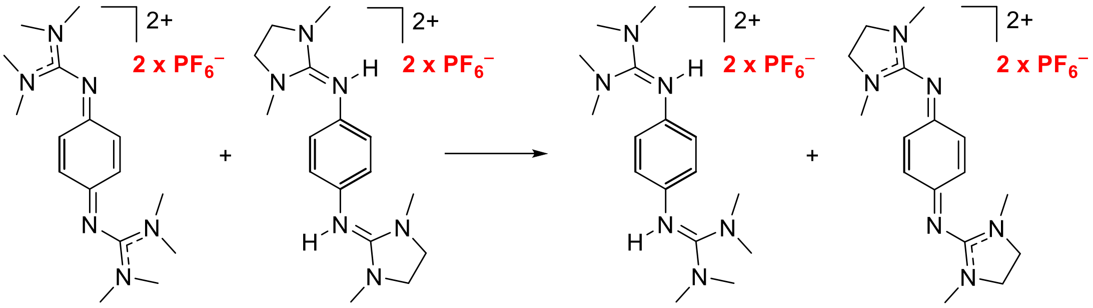

	SV(P)	TZVP[a] (ε_r = 1)
$\Delta_r E$ / kJ mol^{-1}	-82.9	-74.5
$\Delta_r H$ (0 K) / kJ mol^{-1}	-77.4	-69.0
$\Delta_r G$ (298 K) / kJ mol^{-1}	-66.7	-58.2

[a] Thermodynamic contributions with the SV(P) basis set.

Coordinates for calculated structures at ε_r = 1
B3LYP/def2-TZVP

1^{2+}

Illustration of the structure (H atoms omitted)

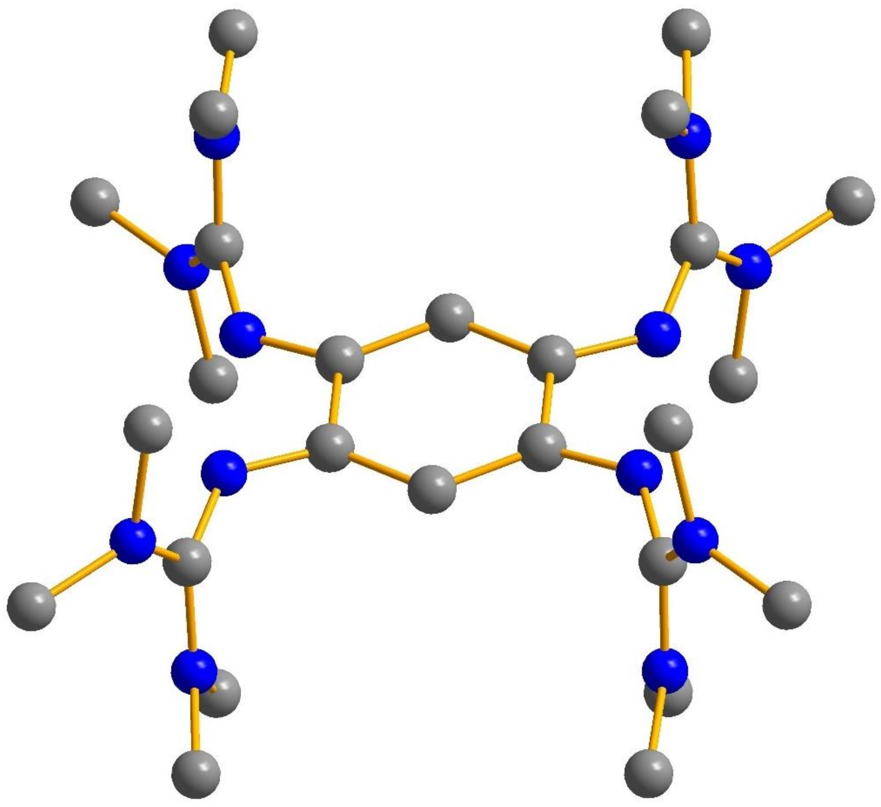

Energy = -1676.926440383 Hartree

Coordinates in Å

N	5.3772867	7.3699338	8.3767889
N	3.8360846	8.2844509	9.8727273
N	3.3813576	8.2312167	7.5758568
N	8.7624617	10.7760595	8.7475595
N	7.9670280	12.8458069	8.0760325
N	7.6756392	12.1474345	10.2925420
C	6.6000361	7.7991042	8.5803999
C	8.3286301	9.5383382	8.7697759
C	6.9867568	9.1375096	8.7281761
H	6.2198912	9.9021776	8.7056338
C	4.2199469	7.9978313	8.6049172
C	4.4995917	7.6597256	11.0139421
H	4.9329797	6.7089529	10.7165890
H	3.7574466	7.4829469	11.7930043
H	5.2857942	8.3005971	11.4235906
C	2.8851651	9.3425836	10.2102493
H	2.6712117	9.9574383	9.3413948
H	3.3340720	9.9766461	10.9781302

H	1.9501614	8.9422016	10.6075390
C	1.9250117	8.0850933	7.6759011
H	1.6435133	7.6954791	8.6488934
H	1.5985088	7.3701700	6.9183523
H	1.4104028	9.0309588	7.4993198
C	3.8979275	8.2097120	6.2085378
H	4.9332924	8.5357859	6.1873498
H	3.3027603	8.8921227	5.6015026
H	3.8293986	7.2071652	5.7775804
C	8.0997767	11.9019550	9.0289925
C	8.1396190	12.4785407	6.6716632
H	9.1813962	12.5930345	6.3599974
H	7.5186215	13.1351014	6.0620028
H	7.8296796	11.4513046	6.5057112
C	8.0846814	14.2831234	8.3455254
H	7.1568533	14.8134093	8.1251011
H	8.8718680	14.6890755	7.7074293
H	8.3659323	14.4589089	9.3788646
C	8.1835715	11.3655129	11.4166072
H	7.5120408	10.5398027	11.6694026
H	8.2681127	12.0198585	12.2847455
H	9.1654918	10.9666860	11.1777893
C	6.5764179	13.0561884	10.6145665
H	6.9212564	13.9434837	11.1495097
H	5.8699822	12.5272726	11.2582974
H	6.0545488	13.3615917	9.7128265
N	10.6269274	8.9009081	8.9527135
N	12.1702708	7.9875887	7.4582929
N	12.6214544	8.0384407	9.7559549
N	7.2420440	5.4947937	8.5807887
N	8.0380073	3.4252841	9.2524588
N	8.3297127	4.1238089	7.0360806
C	9.4042676	8.4717478	8.7484222
C	7.6757310	6.7325305	8.5587029
C	9.0175827	7.1333645	8.6003422
H	9.7844725	6.3687209	8.6226970
C	11.7845444	8.2729784	8.7257641
C	11.5084849	8.6134905	6.3167161
H	11.0743083	9.5637831	6.6145073
H	12.2518769	8.7914105	5.5391041
H	10.7231625	7.9728694	5.9050032
C	13.1219331	6.9300083	7.1211565
H	13.3343834	6.3140825	7.9896245
H	12.6744884	6.2968766	6.3516711
H	14.0576025	7.3310040	6.7260654
C	14.0779892	8.1844980	9.6584933
H	14.3611538	8.5752506	8.6864347
H	14.4033089	8.8984820	10.4174472
H	14.5921892	7.2383561	9.8347996
C	12.1026165	8.0584519	11.1224450
H	11.0671178	7.7326115	11.1415182
H	12.6965964	7.3751629	11.7296679
H	12.1706916	9.0604825	11.5547205
C	7.9050693	4.3691075	8.2994672
C	7.8649391	3.7923720	10.6568230
H	6.8232169	3.6770587	10.9683467

H	8.4863679	3.1362728	11.2665465
H	8.1740561	4.8198360	10.8228452
C	7.9215508	1.9878783	8.9828714
H	8.8497364	1.4583223	9.2035467
H	7.1344987	1.5812730	9.6207142
H	7.6407565	1.8119207	7.9494429
C	7.8219835	4.9056185	5.9118530
H	8.4933377	5.7315434	5.6593521
H	7.7380009	4.2512805	5.0436695
H	6.8398599	5.3041051	6.1502832
C	9.4292764	3.2153370	6.7144548
H	9.0848762	2.3279704	6.1793595
H	10.1358341	3.7444352	6.0710213
H	9.9508618	2.9100352	7.6163909

(**1**+2H)$^{2+}$

Illustration of the structure (C-H atoms omitted)

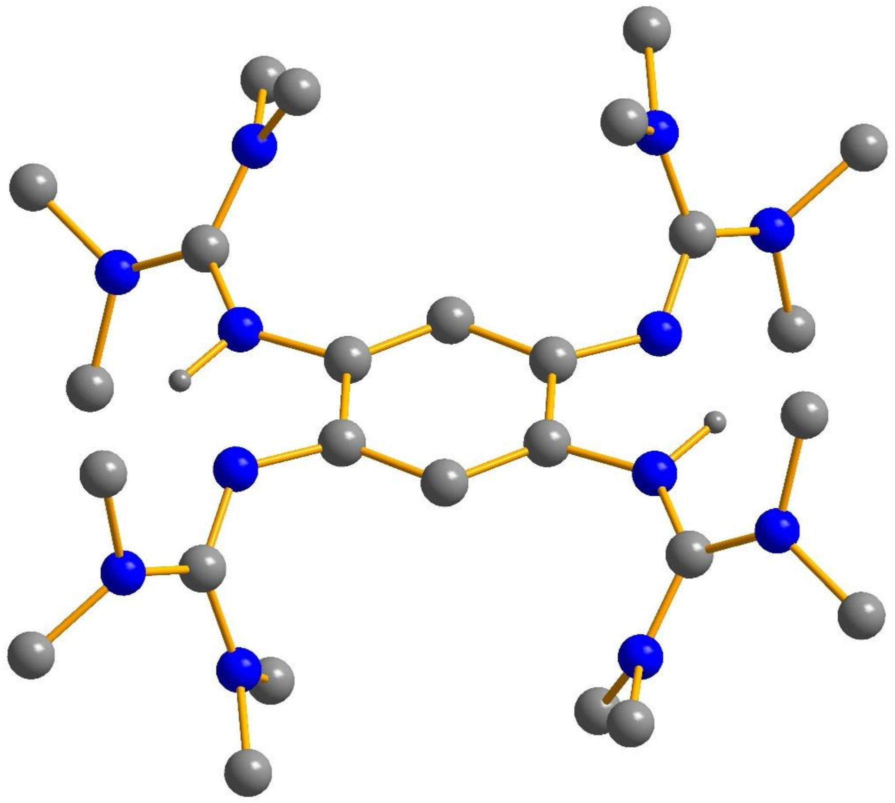

Energy = -1678.160534386 Hartree

<u>Coordinates in Å</u>

N	5.3391681	7.2848772	8.5215870
H	5.3636149	6.2796399	8.3509642
N	3.8944039	8.8683377	9.4865823

N	3.1374786	7.3865319	7.8457314
N	8.8184659	10.8247261	8.6132347
N	8.3846587	13.0802667	8.5490453
N	7.6255744	11.8649292	10.3923618
C	6.6650064	7.7760856	8.6391532
C	8.3608038	9.5206928	8.7001601
C	7.0165860	9.1205637	8.6674215
H	6.2529765	9.8842596	8.6167859
C	4.1290536	7.8628438	8.6222641
C	4.5406255	8.9182850	10.7953884
H	5.1207585	8.0163174	10.9652877
H	3.7706014	8.9941218	11.5661798
H	5.1974520	9.7845405	10.8733657
C	2.9419437	9.9434081	9.2095293
H	2.6489497	9.9285914	8.1637429
H	3.4257737	10.8992979	9.4190051
H	2.0510863	9.8689278	9.8363842
C	1.7437422	7.3259830	8.2971835
H	1.6754832	7.5653023	9.3538583
H	1.3759859	6.3089821	8.1515531
H	1.1091272	8.0064168	7.7268965
C	3.4033847	6.7136005	6.5730546
H	4.3644029	7.0275726	6.1747716
H	2.6232007	6.9914505	5.8638404
H	3.3906514	5.6260286	6.6846970
C	8.2703656	11.8766525	9.1759110
C	8.7060722	13.1296606	7.1274841
H	9.7870388	13.1635077	6.9594636
H	8.2623932	14.0299602	6.6996500
H	8.3062103	12.2552676	6.6235237
C	8.6365065	14.3276675	9.2689834
H	7.8559477	15.0672859	9.0775523
H	9.5890364	14.7480035	8.9357941
H	8.7024298	14.1494833	10.3378441
C	8.0251494	10.9425467	11.4422022
H	7.3339753	10.1022714	11.5543007
H	8.0684919	11.4780334	12.3936779
H	9.0126393	10.5415986	11.2289785
C	6.5221714	12.7569949	10.7231900
H	6.7955190	13.4926592	11.4850220
H	5.6862023	12.1721338	11.1185066
H	6.1837305	13.2825677	9.8344022
N	10.6659056	8.9859542	8.8101853
H	10.6418630	9.9908997	8.9824320
N	12.1099196	7.4046247	7.8407230
N	12.8677115	8.8817755	9.4854061
N	7.1866318	5.4463826	8.7177562
N	7.6206558	3.1908376	8.7812936
N	8.3794171	4.4067468	6.9382518
C	9.3400092	8.4950507	8.6919351
C	7.6442511	6.7504531	8.6308161
C	8.9884278	7.1505513	8.6634788
H	9.7520978	6.3868906	8.7140477
C	11.8758569	8.4078520	8.7078027
C	11.4628140	7.3581304	6.5322132
H	10.8826473	8.2605923	6.3650495

H	12.2323110	7.2842050	5.7607097
H	10.8058535	6.4921328	6.4524662
C	13.0622245	6.3285719	8.1144651
H	13.3554306	6.3402899	9.1602339
H	12.5781376	5.3734079	7.9023064
H	13.9529675	6.4046763	7.4876447
C	14.2614150	8.9429502	9.0339561
H	14.3293561	8.7071195	7.9764708
H	14.6297969	9.9592379	9.1829181
H	14.8957303	8.2602536	9.6018690
C	12.6022907	9.5509860	10.7601517
H	11.6409338	9.2366679	11.1573485
H	13.3821506	9.2702280	11.4685857
H	12.6160312	10.6388846	10.6518718
C	7.7347686	4.3946558	8.1547878
C	7.2992194	3.1409093	10.2028337
H	6.2182633	3.1066246	10.3708038
H	7.7431996	2.2406374	10.6304155
H	7.6987450	4.0153018	10.7070493
C	7.3692977	1.9435598	8.0609801
H	8.1500159	1.2041072	8.2524206
H	6.4168060	1.5228736	8.3938383
H	7.3036027	2.1220093	6.9921503
C	7.9794895	5.3292513	5.8886680
H	8.6700622	6.1700840	5.7770609
H	7.9367805	4.7940815	4.9369881
H	6.9916551	5.7293919	6.1017789
C	9.4834216	3.5154652	6.6073728
H	9.2107606	2.7801544	5.8449515
H	10.3193070	4.1009776	6.2128055
H	9.8217271	2.9894823	7.4959700

2^{2+}

Illustration of the structure (H atoms omitted)

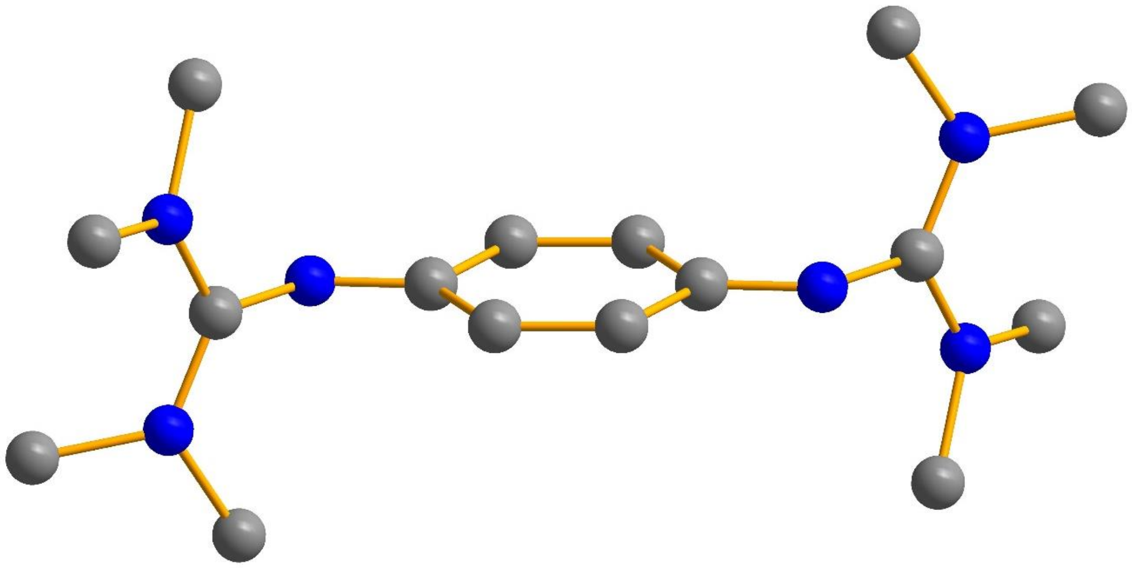

Energy = -954.2570088263 Hartree

Coordinates in Å

N	-0.7063698	-2.0952223	12.6924688
C	0.4534723	-2.4619936	13.2431629
C	-0.7807956	-1.6313667	11.2988135
C	-1.9686378	-1.9986718	13.4395347
N	1.6080522	-2.1596044	12.5509123
N	0.5427902	-3.2195847	14.3391338
H	-0.7831850	-0.5405186	11.2493468
H	-1.7110746	-2.0002701	10.8688296
H	0.0510444	-2.0252361	10.7225680
H	-2.6593277	-2.7970962	13.1665834
H	-2.4291640	-1.0435675	13.1857630
H	-1.7863496	-2.0186992	14.5091189
C	2.3497210	-1.1165276	12.6634388
C	1.8147883	-3.3686241	15.0601402
C	-0.4601459	-4.2450101	14.6862928
C	2.1307136	-0.0169416	13.6056960
C	3.5162480	-1.0290760	11.7764245
H	2.4151898	-4.1720102	14.6274673
H	1.5864412	-3.6168318	16.0949382
H	2.3839102	-2.4446321	15.0559862
H	-0.9990494	-3.9826605	15.5965393
H	0.0705037	-5.1826897	14.8522852
H	-1.1588029	-4.3927958	13.8701329
H	1.2720004	-0.0606855	14.2643226
C	2.9693031	1.0279707	13.6481956
C	4.3547910	0.0158696	11.8188757
H	3.6676859	-1.8541380	11.0921254
C	4.1355922	1.1156192	12.7608907

H	2.8179421	1.8529697	14.3325848
H	5.2136015	0.0595410	11.1603720
N	4.8768375	2.1589933	12.8728682
C	6.0308072	2.4618653	12.1798594
N	7.1912255	2.0963393	12.7301414
N	5.9403702	3.2188097	11.0835271
C	7.2667756	1.6333095	14.1240111
C	8.4531435	2.0003621	11.9824082
C	4.6679599	3.3665984	10.3630098
C	6.9424390	4.2447236	10.7352996
H	7.2699130	0.5424923	14.1741020
H	8.1970650	2.0030734	14.5532260
H	6.4350133	2.0269495	14.7005251
H	9.1433463	2.7995041	12.2544864
H	8.9145880	1.0457765	12.2364613
H	8.2702196	2.0196174	10.9129197
H	4.0675043	4.1702383	10.7951352
H	4.8956713	3.6137795	9.3278210
H	4.0991621	2.4424245	10.3684607
H	7.4812099	3.9821588	9.8250369
H	6.4110447	5.1818987	10.5688540
H	7.6412792	4.3935695	11.5511145

$(\mathbf{2}+2H)^{2+}$

Illustration of the structure (C-H atoms omitted)

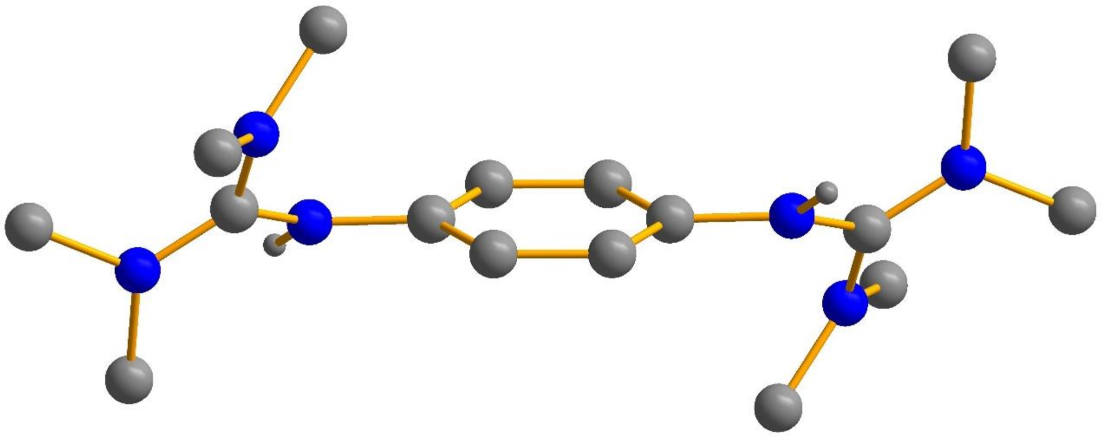

Energy = -955.5276406660 Hartree

<u>Coordinates in Å</u>

N	-0.2997228	-2.0642835	13.0222334
C	0.6975157	-2.7325301	13.6168141
C	-0.1883796	-1.5312197	11.6612663
C	-1.5704292	-1.7774722	13.6983580
N	1.9947079	-2.4587309	13.3034304
N	0.4608517	-3.7047322	14.5064708
H	-0.0911803	-0.4440495	11.6701145
H	-1.0949517	-1.7952383	11.1157213

H	0.6638248	-1.9676810	11.1493305
H	-2.3909357	-2.3566059	13.2727197
H	-1.7950498	-0.7178187	13.5666343
H	-1.4915467	-1.9853986	14.7612344
H	2.6356060	-3.2337242	13.3895373
C	2.5908306	-1.2039400	12.9978796
C	1.4418746	-4.0891895	15.5292596
C	-0.7214900	-4.5746301	14.4299628
C	2.1310086	-0.0095846	13.5499060
C	3.7069681	-1.1841037	12.1620075
H	1.9998692	-4.9796529	15.2285290
H	0.9063356	-4.3198646	16.4498899
H	2.1299235	-3.2714848	15.7271994
H	-1.4125961	-4.3795755	15.2507495
H	-0.3861602	-5.6100386	14.4985662
H	-1.2310606	-4.4406398	13.4809429
H	1.2862313	-0.0020181	14.2254961
C	2.7784427	1.1836454	13.2638361
C	4.3544524	0.0091251	11.8759909
H	4.0641749	-2.1020842	11.7113843
C	3.8945039	1.2035061	12.4278563
H	2.4212098	2.1016270	13.7144273
H	5.1992587	0.0015184	11.2004466
N	4.4901741	2.4583966	12.1220048
H	3.8490265	3.2332728	12.0368118
C	5.7868112	2.7324715	11.8065294
N	6.7851548	2.0646373	12.3996843
N	6.0217655	3.7045594	10.9162902
C	6.6760160	1.5316572	13.7608697
C	8.0549913	1.7782911	11.7217210
C	5.0390492	4.0883509	9.8948662
C	7.2039231	4.5748999	10.9906884
H	6.5793397	0.4444391	13.7522488
H	7.5832386	1.7961643	14.3050923
H	5.8243204	1.9677373	14.2739669
H	8.8758408	2.3580260	12.1458760
H	8.2803982	0.7188137	11.8534961
H	7.9743456	1.9857808	10.6588888
H	4.4812952	4.9787880	10.1961141
H	5.5730932	4.3187968	8.9733068
H	4.3509119	3.2703870	9.6983385
H	7.8938582	4.3797758	10.1689317
H	6.8681134	5.6101592	10.9221899
H	7.7149688	4.4414646	11.9389938

3^{2+}

Illustration of the structure (H atoms omitted)

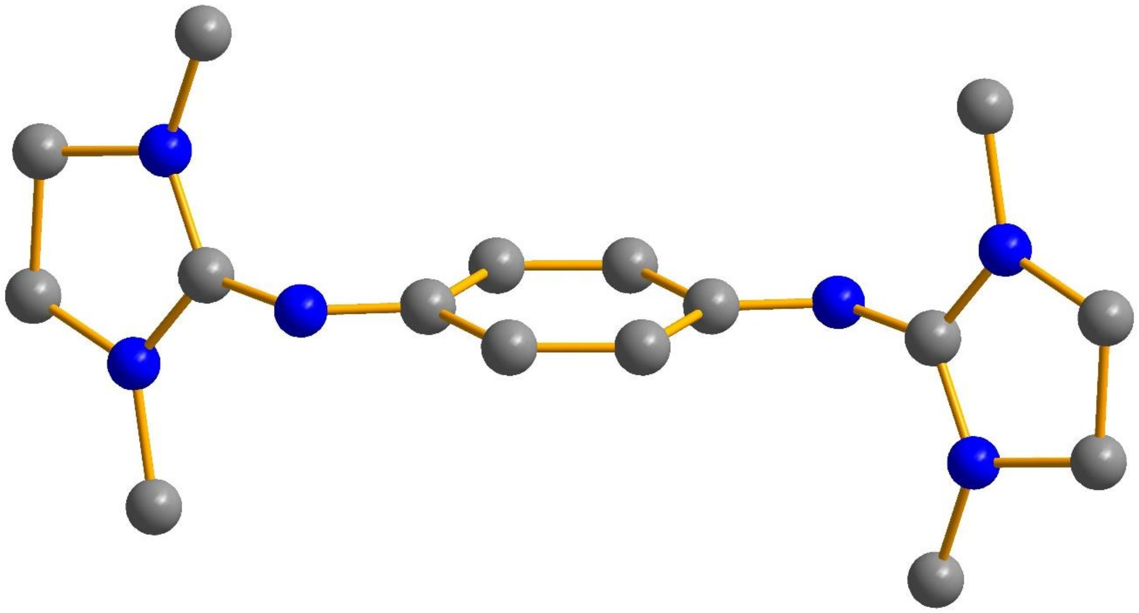

Energy = -951.8854033714 Hartree

<u>Coordinates in Å</u>

N	-1.3780068	9.0570172	6.0944071
C	-2.4165962	8.2043387	6.3281864
C	-1.3103464	9.9742513	5.1938992
N	-2.3839569	6.9234249	5.9836960
N	-3.4925160	8.5154429	7.0542878
C	-0.0892282	10.7874522	5.1662608
C	-2.3447292	10.2619560	4.1988273
C	-3.5994552	6.2367310	6.4571222
C	-1.3883845	6.2599634	5.1547409
C	-4.2338757	7.2822816	7.3901847
C	-3.7080364	9.7373695	7.8181542
H	0.6619087	10.5600726	5.9119026
C	0.0822937	11.7639512	4.2641287
C	-2.1734421	11.2388285	3.2970491
H	-3.2511473	9.6696465	4.2052937
H	-3.3350263	5.3139107	6.9716401
H	-4.2380173	5.9901520	5.6061167
H	-1.7892920	6.0471993	4.1614083
H	-1.1011547	5.3204983	5.6266753
H	-0.5064823	6.8894425	5.0667972
H	-5.2991807	7.4205212	7.2132703
H	-4.0824692	7.0456657	8.4461172
H	-3.5067891	9.5621923	8.8777221
H	-4.7433052	10.0570096	7.7028705
H	-3.0522391	10.5261721	7.4610833
C	-0.9519674	12.0514760	3.2688884
H	0.9887558	12.3561892	4.2575852

H	-2.9247346	11.4664539	2.5516466
N	-0.8836430	12.9678341	2.3675811
C	0.1560462	13.8187920	2.1324973
N	0.1271359	15.0994817	2.4780873
N	1.2295050	13.5058181	1.4035727
C	1.3429556	15.7838543	2.0020247
C	-0.8646316	15.7645491	3.3103155
C	1.9728181	14.7375320	1.0667546
C	1.4400246	12.2840977	0.6379687
H	1.0792589	16.7075707	1.4887467
H	1.9841450	16.0285055	2.8516110
H	-0.4603264	15.9759465	4.3025616
H	-1.1513163	16.7048638	2.8397476
H	-1.7475998	15.1368968	3.4005566
H	3.0382304	14.5967990	1.2410227
H	1.8194106	14.9752243	0.0113515
H	1.2359841	12.4606663	-0.4208344
H	2.4749146	11.9620558	0.7498761
H	0.7835532	11.4964598	0.9963423

(**3**+2H)$^{2+}$

Illustration of the structure (C-H atoms omitted)

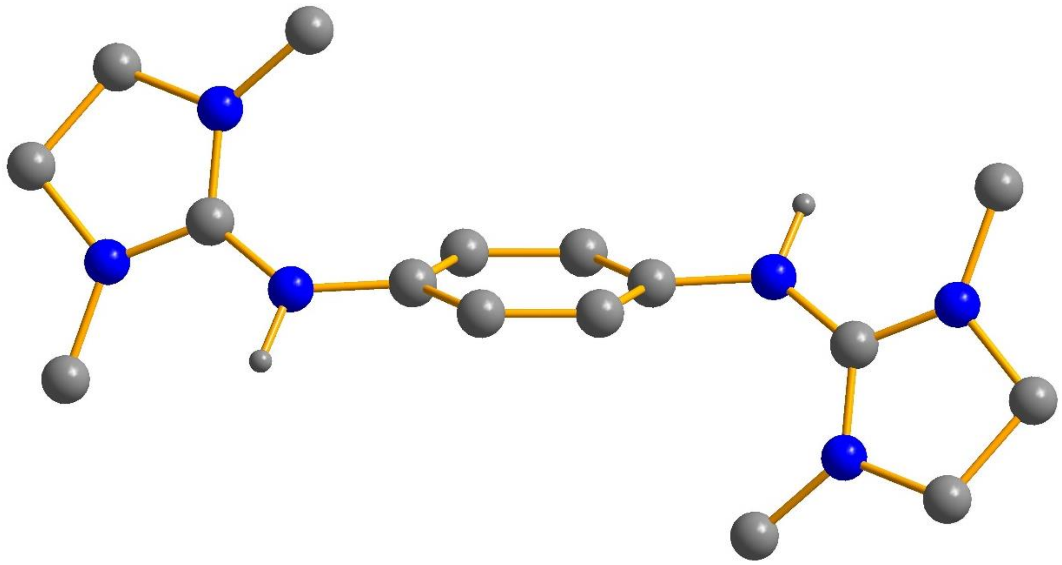

Energy = -953.1405316858 Hartree

<u>Coordinates in Å</u>

N	2.6553347	0.8101912	-0.4942124
C	1.3234575	0.3652408	-0.2389467
C	3.8197527	0.1950616	-0.1838485
C	0.3252287	0.6889878	-1.1560288
C	0.9915698	-0.3215970	0.9270841
N	4.9191423	0.8796029	0.1460725
H	0.5777838	1.2054950	-2.0736655

C	-0.9920601	0.3238565	-0.9161913
C	-0.3257107	-0.6867489	1.1669071
C	-1.3238973	-0.3631202	0.2497431
H	-1.7485402	0.5517690	-1.6556577
H	-0.5782298	-1.2032356	2.0845427
N	-2.6556355	-0.8080781	0.5050685
C	-3.8198383	-0.1949077	0.1895919
H	-2.7432062	-1.7667069	0.8071610
N	-4.0370881	1.1218642	0.2004079
N	-4.9173503	-0.8806320	-0.1436649
C	-6.0753837	0.0260920	-0.2183909
C	-5.0551847	-2.3261541	-0.2327345
C	-5.4012852	1.4042501	-0.2882017
C	-3.1792819	2.1742067	0.7245409
C	6.0767936	-0.0280929	0.2141989
C	5.0579903	2.3252235	0.2326486
C	5.4016827	-1.4055135	0.2869912
H	2.7431029	1.7697877	-0.7932991
N	4.0359774	-1.1218741	-0.1967199
H	1.7481025	-0.5493279	1.6665932
H	-6.7060971	-0.0907839	0.6665696
H	-6.6690416	-0.1964900	-1.1030355
H	-4.1227350	-2.7764211	-0.5717698
H	-5.8207683	-2.5585565	-0.9711225
H	-5.3515943	-2.7640101	0.7241939
H	-5.3638510	1.8006898	-1.3053423
H	-5.8803379	2.1399409	0.3550281
H	-2.4371121	1.7561935	1.3986852
H	-3.7971512	2.8746563	1.2858088
H	-2.6752204	2.7171749	-0.0776293
H	6.7027582	0.0884105	-0.6742009
H	6.6754555	0.1934683	1.0957002
H	4.1310311	2.7753057	0.5868780
H	5.8352912	2.5575455	0.9586186
H	5.3391950	2.7634054	-0.7287721
H	5.3675267	-1.8014002	1.3044787
H	5.8776267	-2.1419599	-0.3576648
C	3.1746230	-2.1737030	-0.7160066
H	2.4313929	-1.7558475	-1.3891134
H	3.7896695	-2.8765032	-1.2774029
H	2.6717616	-2.7140237	0.0887180

9,10-Dihydroanthracene (AnH$_2$)

Illustration of the structure

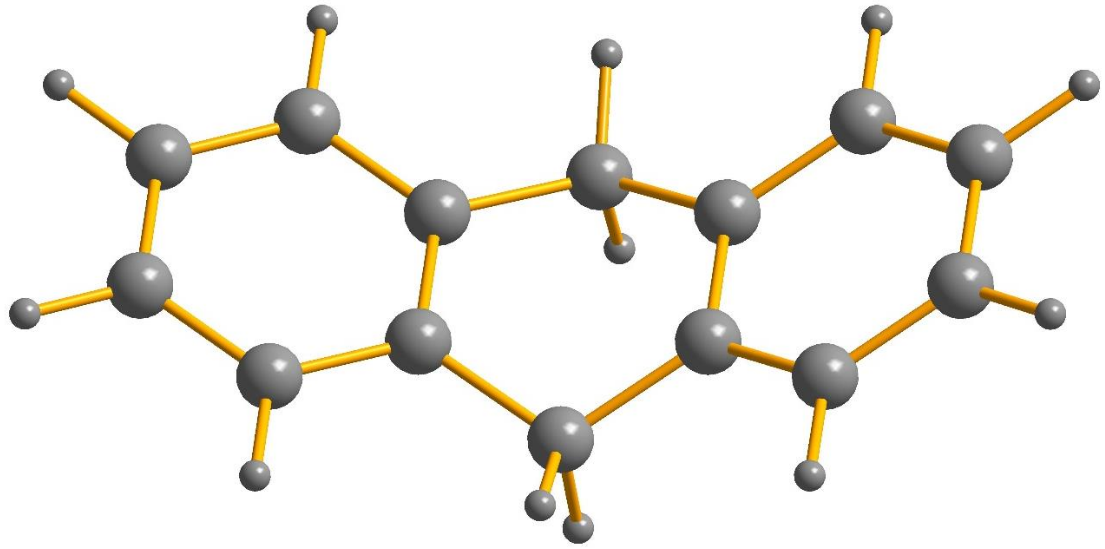

Energy = -540.5756602807 Hartree

<u>Coordinates in Å</u>

C	-3.5517567	-0.6950504	0.5093367
C	-3.5516430	0.6952968	0.5095286
C	-2.4048958	-1.3847239	0.1333931
C	-2.4047453	1.3848866	0.1335425
C	-1.2547342	-0.6995146	-0.2521256
C	-1.2546816	0.6995940	-0.2521224
C	-0.0000456	-1.4257459	-0.6786289
C	0.0000518	1.4257388	-0.6786426
C	1.2546978	-0.6995967	-0.2521457
C	1.2547499	0.6995115	-0.2521609
C	2.4047397	-1.3848860	0.1335907
C	2.4048842	1.3847251	0.1334323
C	3.5516583	-0.6952925	0.5095064
C	3.5517643	0.6950553	0.5093242
H	-4.4382069	-1.2407360	0.8082978
H	-4.4378866	1.2410446	0.8089893
H	-2.4009959	-2.4691637	0.1429655
H	-2.4007389	2.4693260	0.1431965
H	-0.0000534	-1.5174611	-1.7746783
H	-0.0000755	-2.4466647	-0.2920560
H	0.0000445	1.5174423	-1.7746929
H	0.0000890	2.4466616	-0.2920804
H	2.4006904	-2.4693241	0.1433832
H	2.4009384	2.4691636	0.1431306
H	4.4379420	-1.2410376	0.8088531
H	4.4382329	1.2407437	0.8082254

Anthracene (An)

Illustration of the structure

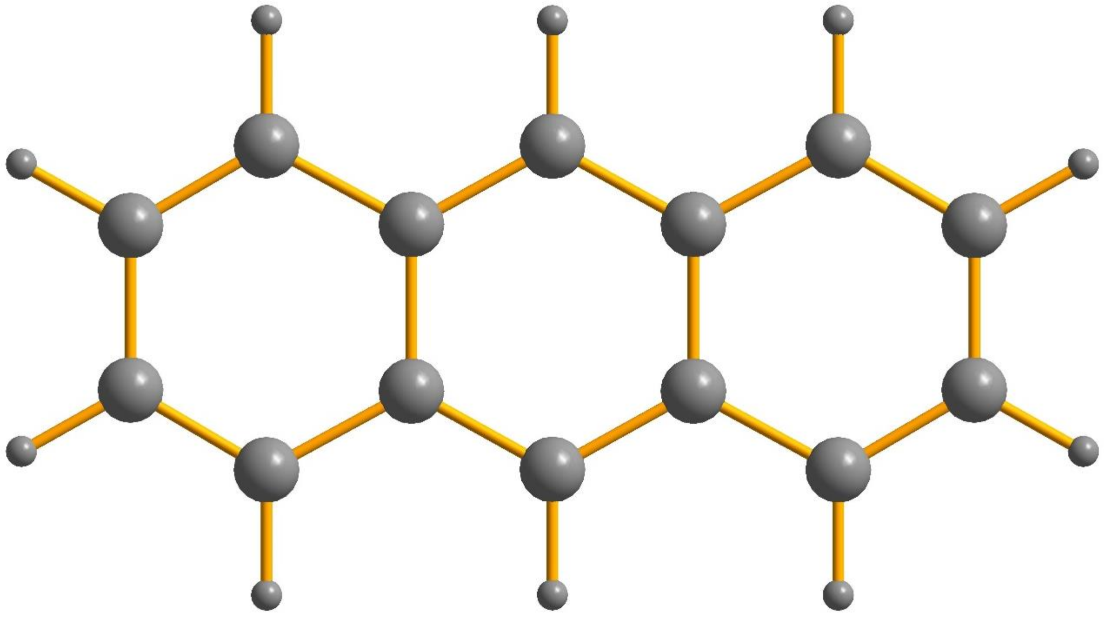

Energy = -539.3765823014 Hartree

Coordinates in Å

C	-3.6481434	-0.7109626	-0.0000216
C	-3.6481435	0.7109624	-0.0001442
C	-2.4721345	-1.4020408	0.0000805
C	-2.4721347	1.4020406	-0.0001289
C	-1.2195522	-0.7201705	0.0000941
C	-1.2195524	0.7201702	0.0000272
C	0.0000002	-1.3988299	0.0001660
C	-0.0000003	1.3988302	0.0001538
C	1.2195524	-0.7201699	0.0000568
C	1.2195523	0.7201708	0.0001142
C	2.4721344	-1.4020405	-0.0001016
C	2.4721349	1.4020408	0.0001057
C	3.6481433	-0.7109627	-0.0001600
C	3.6481436	0.7109624	-0.0000189
H	-4.5915081	-1.2427992	-0.0000030
H	-4.5915082	1.2427990	-0.0002437
H	-2.4716422	-2.4859510	0.0001640
H	-2.4716423	2.4859508	-0.0002238
H	0.0000005	-2.4836567	0.0002142
H	-0.0000005	2.4836570	0.0002054
H	2.4716417	-2.4859507	-0.0002148
H	2.4716428	2.4859510	0.0001599
H	4.5915079	-1.2427994	-0.0002882
H	4.5915083	1.2427988	0.0000072

p-Dihydro-benzoquinone

Illustration of the structure

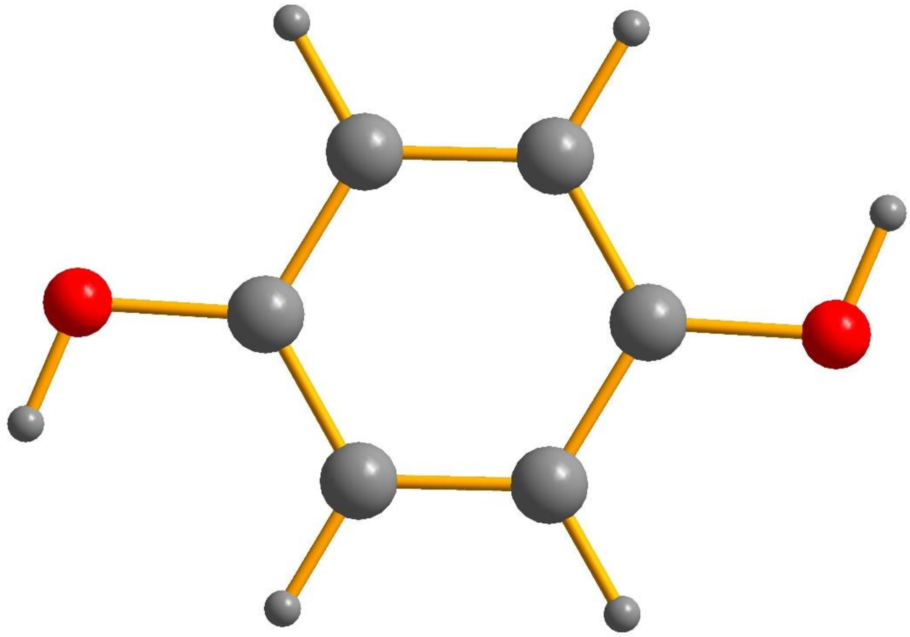

Energy = -382.6309021723 Hartree

Coordinates in Å

C	1.3959206	-0.0293616	0.0000988
C	0.7162670	1.1861318	0.0002096
C	-0.6727162	1.2169398	0.0001333
C	-1.3959207	0.0293617	0.0000212
C	-0.7162671	-1.1861319	0.0000233
C	0.6727162	-1.2169396	0.0001504
O	2.7650095	-0.1165930	-0.0002520
H	1.2716448	2.1181840	0.0001779
H	-1.2048008	2.1591512	-0.0000759
O	-2.7650095	0.1165929	-0.0000579
H	-1.2716449	-2.1181843	-0.0001015
H	1.2048009	-2.1591508	0.0002536
H	3.1445676	0.7689073	-0.0004212
H	-3.1445675	-0.7689075	-0.0001595

p-Benzoquinone

Illustration of the structure

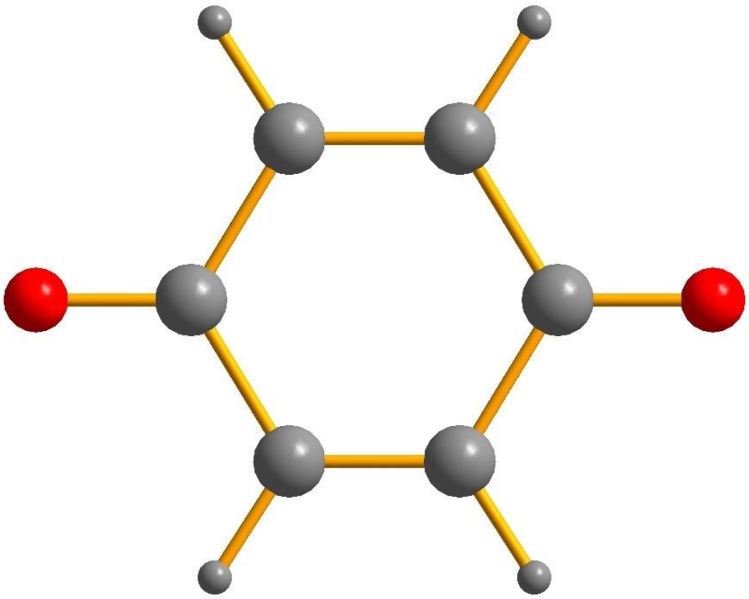

Energy = -381.3976586665 Hartree

<u>Coordinates in Å</u>

C	1.4394615	-0.0000004	-0.0002648
C	0.6681633	1.2661677	0.0003196
C	-0.6681607	1.2661685	0.0003945
C	-1.4394562	0.0000005	-0.0001927
C	-0.6681624	-1.2661682	0.0003165
C	0.6681617	-1.2661680	0.0003486
O	2.6573346	-0.0000015	-0.0014168
H	1.2539444	2.1774916	0.0004059
H	-1.2539457	2.1774944	0.0005065
O	-2.6573381	0.0000017	-0.0012447
H	-1.2539460	-2.1774949	0.0003697
H	1.2539435	-2.1774914	0.0004575

Calculations in the context of the TD-DFT calculations for 2, $2^{\cdot+}$ and 2^{2+}

$2^{\cdot+}$

Illustration of the structure (C-H atoms omitted)

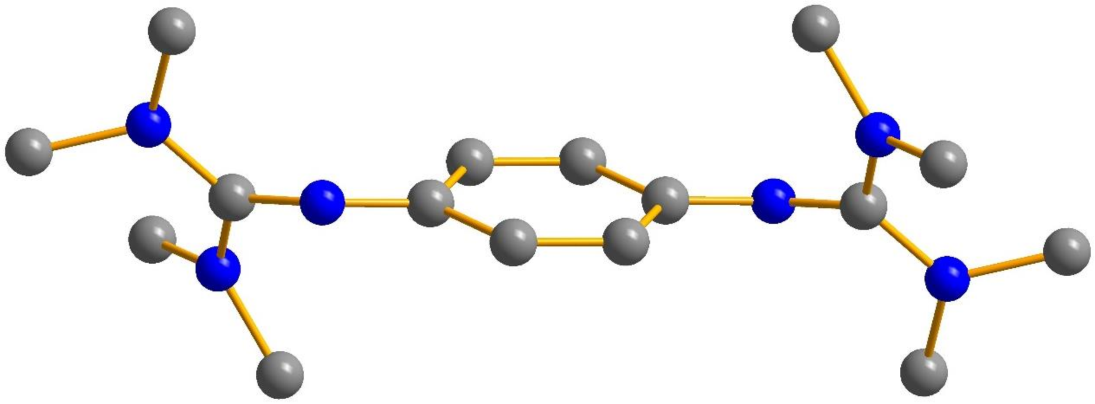

Energy = -954.5791788698

Coordinates in Å

C	-0.9746466	-0.5134201	-0.8648086
C	0.3440485	-0.7304163	-1.1381192
C	1.3802165	-0.2223415	-0.2937311
C	0.9746466	0.5134201	0.8648086
C	-0.3440485	0.7304163	1.1381192
C	-1.3802165	0.2223415	0.2937311
H	-1.7319530	-0.8896220	-1.5410543
H	0.6452856	-1.2900397	-2.0144217
H	1.7319530	0.8896220	1.5410543
H	-0.6452856	1.2900397	2.0144217
N	2.6475415	-0.5226535	-0.6047154
N	-2.6475415	0.5226535	0.6047154
C	3.7357685	0.1117220	-0.1720375
C	-3.7357685	-0.1117220	0.1720375
N	3.8725660	1.4632918	-0.2597121
N	4.7753553	-0.6262578	0.2759694
N	-3.8725660	-1.4632918	0.2597121
N	-4.7753553	0.6262578	-0.2759694
C	3.0564517	2.2504430	-1.1784019
H	2.2135848	2.7259818	-0.6690736
H	3.6805068	3.0318984	-1.6153650
H	2.6781253	1.6196941	-1.9777773
C	4.7011968	2.2447556	0.6548349
H	5.5558168	2.6995584	0.1480740
H	4.0917151	3.0478072	1.0769235
H	5.0586375	1.6236350	1.4708388
C	4.5524011	-2.0150561	0.6713515
H	4.6753587	-2.6920498	-0.1786441

H	5.2792462	-2.2783400	1.4405039
H	3.5520989	-2.1381875	1.0742492
C	6.1721218	-0.2844695	0.0067022
H	6.7273544	-0.0935373	0.9272856
H	6.6426669	-1.1244944	-0.5089638
H	6.2362330	0.5859825	-0.6387859
C	-4.7011968	-2.2447556	-0.6548349
H	-4.0917151	-3.0478072	-1.0769235
H	-5.5558168	-2.6995584	-0.1480740
H	-5.0586375	-1.6236350	-1.4708388
C	-3.0564517	-2.2504430	1.1784019
H	-3.6805068	-3.0318984	1.6153650
H	-2.2135848	-2.7259818	0.6690736
H	-2.6781253	-1.6196941	1.9777773
C	-4.5524011	2.0150561	-0.6713515
H	-5.2792462	2.2783400	-1.4405039
H	-4.6753587	2.6920498	0.1786441
H	-3.5520989	2.1381875	-1.0742492
C	-6.1721218	0.2844695	-0.0067022
H	-6.6426669	1.1244944	0.5089638
H	-6.7273544	0.0935373	-0.9272856
H	-6.2362330	-0.5859825	0.6387859

Electronic transitions of **2**$^{\cdot+}$ (^2A_g) found in the TD-DFT calculations (B3LYP/TZVP), IRREP a_u

Only electronic transitions above 300 nm are listed

λ/nm	oscillator strength	leading contribution	
542.6	0.0451	$41a_u$ (β) → $42a_g$ (β)	54.6%
501.3	0.186	$40a_u$ (β) → $42a_g$ (β)	68.7%
467.9	0.00425	$39a_u$ (β) → $42a_g$ (β)	58.3%
364.9	0.495	$42a_g$ (α) → $42a_u$ (α)	82.0%
346.3	0.141	$42a_g$ (α) → $43a_u$ (α)	92.5%

Simulation of the electronic excitation spectrum of **2**$^{\cdot+}$ (^2A_g)

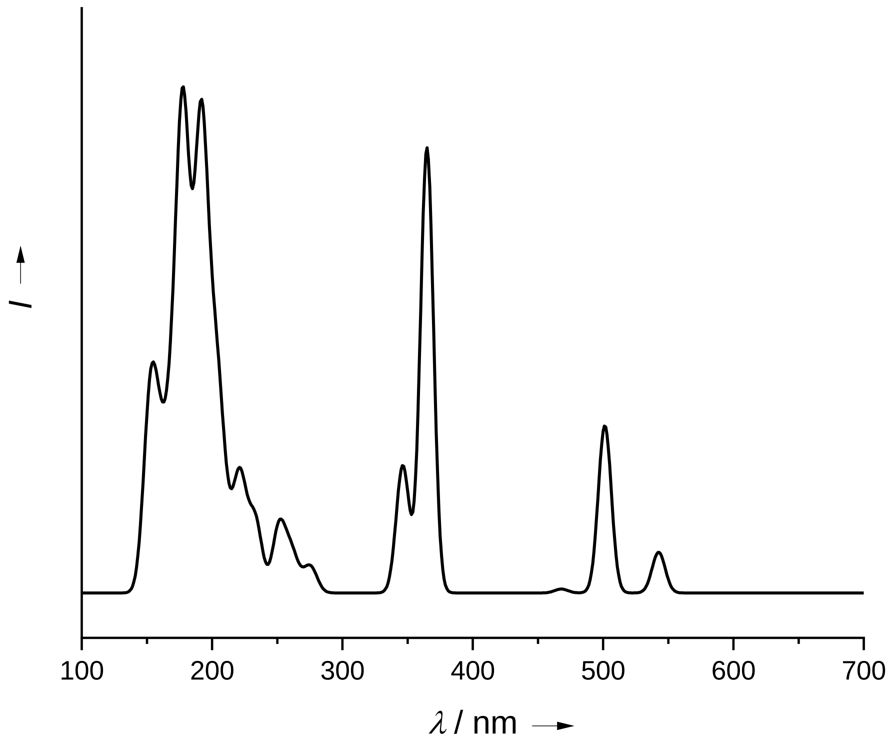

Isodensity plots for the relavant orbitals of **2**$^{\cdot+}$ (^2A_g). Contour values for the isodensity plots are ±0.02 Bohr$^{(-3/2)}$.

<table>
<tr><td colspan="2">λ = 542.6 nm (54.6% 41a$_u$ (β) $\rightarrow$ 42a$_g$ (β))</td></tr>
<tr><td>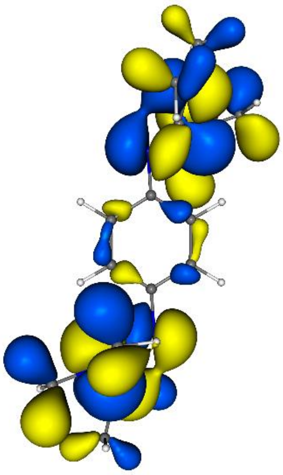
41a$_u$ (β)</td><td>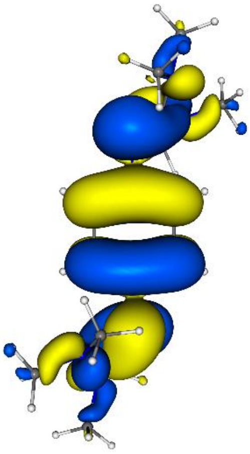
42a$_g$ (β)</td></tr>
<tr><td colspan="2">λ = 501.3nm (68.7% 40a$_u$ (β) $\rightarrow$ 42a$_g$ (β))</td></tr>
<tr><td>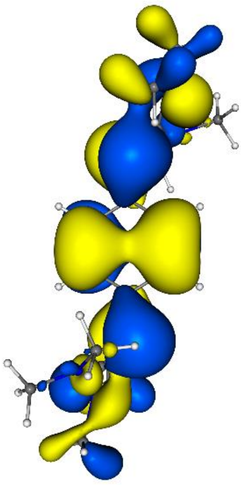
40a$_u$ (β)</td><td>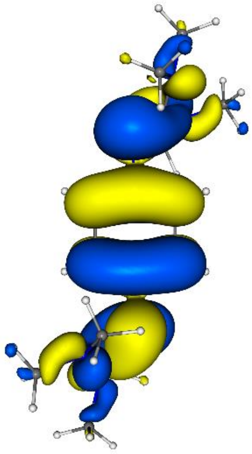
42a$_g$ (β)</td></tr>
<tr><td colspan="2">λ = 467.9 nm (58.3% 39a$_u$ (β) $\rightarrow$ 42a$_g$ (β))</td></tr>
<tr><td>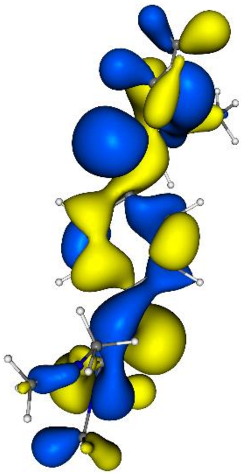
39a$_u$ (β)</td><td>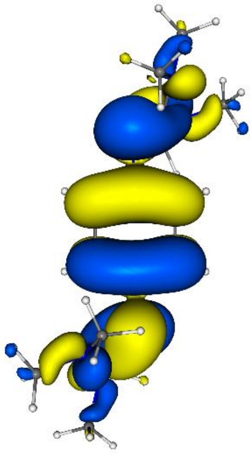
42a$_g$ (β)</td></tr>
</table>

λ = 364.9 nm (82.0% $42a_g$ (α) $\rightarrow$ $42a_u$ (α))

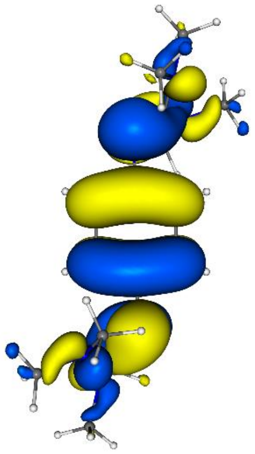

$42a_g$ (α)

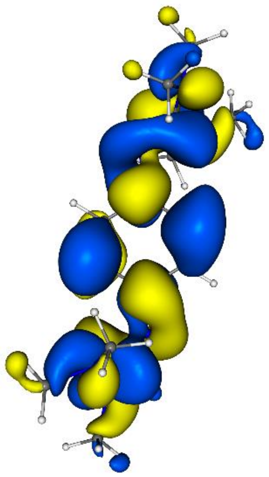

$42a_u$ (α)

λ = 346.3 nm (92.5% $42a_g$ (α) $\rightarrow$ $43a_u$ (α))

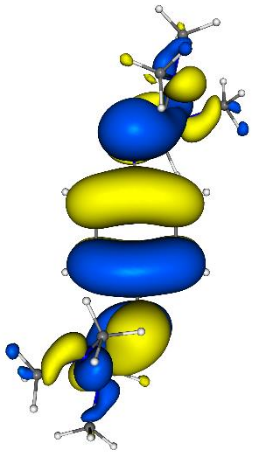

$42a_g$ (α)

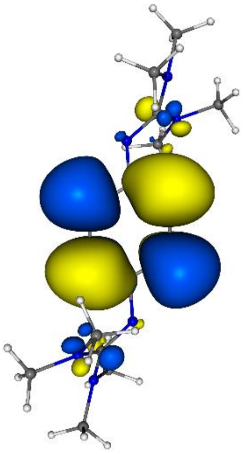

$43a_u$ (α)

The neutral compound exhibits no electronic transitions in the visible region.

First two lowest energetic electronic transitions of **2** (^1A_g) found in the TD-DFT calculations (B3LYP/TZVP), IRREP a_u

λ/nm	oscillator strength
298.6	0.673
292.2	0.0228

Simulation of the electronic excitation spectrum of **2** (^1A_g)

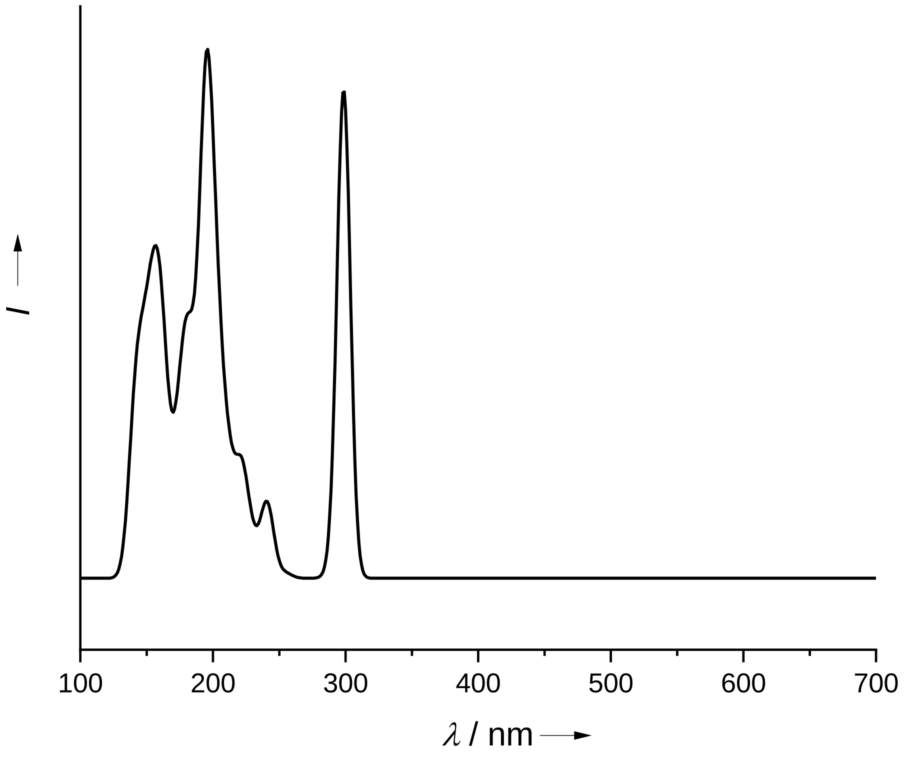

Electronic transitions of **2**$^{2+}$ ($^{1}A_{g}$) found in the TD-DFT calculations (B3LYP/TZVP), IRREP a_u

Only electronic transitions above 300 nm are listed

λ/nm	oscillator strength	leading contribution		
522.3	0.00359	$41a_u \rightarrow 42a_g$	HOMO-1 → LUMO	98.8%
398.5	0.0121	$40a_u \rightarrow 42a_g$	HOMO-2 → LUMO	59.9%

Simulation of the electronic excitation spectrum of **2**$^{2+}$ ($^{1}A_{g}$)

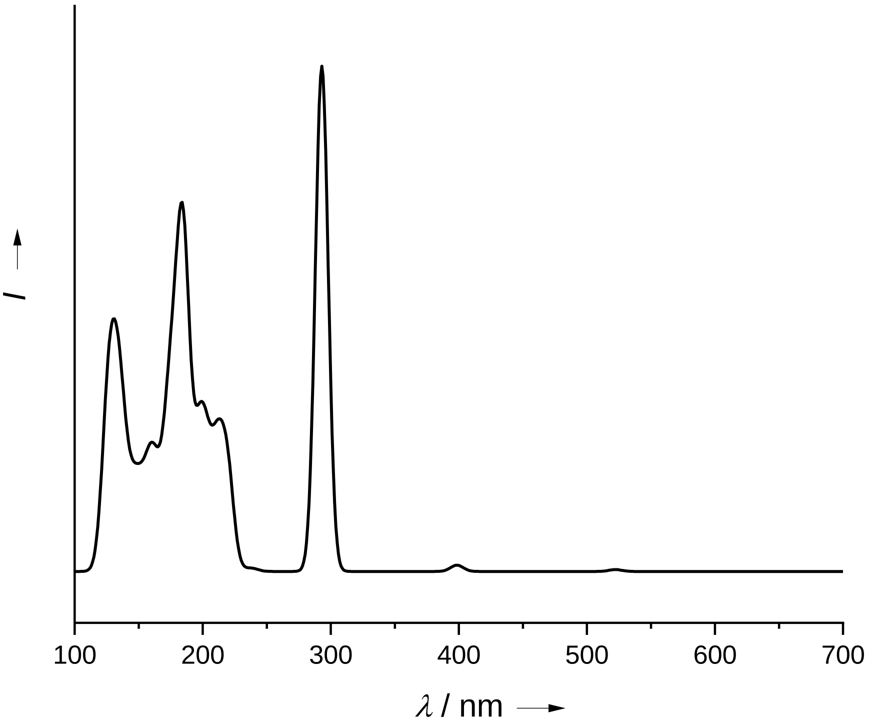

Supplement: Supplementary file 1 — Supplementary [file CHEM-26-16504-s001.pdf]
